# Supplementary material for: Survival of dental implants in irradiated head and neck cancer patients compared to non-irradiated patients: An umbrella review
Source: PLoS One. 2025 Sep 3;20(9):e0324388. doi: 10.1371/journal.pone.0324388 (PMC12407548; doi:10.1371/journal.pone.0324388)
Supplement: S1 Text — (PDF) [file pone.0324388.s001.pdf]

SUPPORTING INFORMATION

S1 – Search strategy in electronic databases

Electronic databases	Search strategy
PubMed	("dental implants"[MeSH Terms] OR ("dental"[All Fields] AND "implants"[All Fields]) OR "dental implants"[All Fields] OR ("dental implants"[MeSH Terms] OR ("dental"[All Fields] AND "implants"[All Fields]) OR "dental implants"[All Fields] OR ("dental"[All Fields] AND "implant"[All Fields]) OR "dental implant"[All Fields]) OR ("dental implantation"[MeSH Terms] OR ("dental"[All Fields] AND "implantation"[All Fields]) OR "dental implantation"[All Fields]) OR ("dental prosthesis, implant supported"[MeSH Terms] OR ("dental"[All Fields] AND "prosthesis"[All Fields] AND "implant supported"[All Fields]) OR "implant-supported dental prosthesis"[All Fields] OR ("implant"[All Fields] AND "supported"[All Fields] AND "dental"[All Fields] AND "prosthesis"[All Fields]) OR "implant supported dental prosthesis"[All Fields]) OR ("dental implantation, endosseous"[MeSH Terms] OR ("dental"[All Fields] AND "implantation"[All Fields] AND "endosseous"[All Fields]) OR "endosseous dental implantation"[All Fields] OR ("endosseous"[All Fields] AND "dental"[All Fields] AND "implantation"[All Fields])) OR (("dental implants"[MeSH Terms] OR ("dental"[All Fields] AND "implants"[All Fields]) OR "dental implants"[All Fields] OR ("dental"[All Fields] AND "implant"[All Fields]) OR "dental implant"[All Fields]) AND ("mortality"[MeSH Subheading] OR "mortality"[All Fields] OR "survival"[All Fields] OR "survival"[MeSH Terms] OR "survivability"[All Fields] OR "survivable"[All Fields] OR "survivals"[All Fields] OR "survive"[All Fields] OR "survived"[All Fields] OR "survives"[All Fields] OR "surviving"[All Fields])) OR ("dental implants"[MeSH Terms] OR ("dental"[All Fields] AND "implants"[All Fields]) OR "dental implants"[All Fields] OR ("surgical"[All Fields] AND "dental"[All Fields] AND "prostheses"[All Fields])) OR ("dental implants"[MeSH Terms] OR ("dental"[All Fields] AND "implants"[All Fields]) OR "dental implants"[All Fields] OR ("surgical"[All Fields] AND "dental"[All Fields] AND "prosthesis"[All Fields]) OR "surgical dental prosthesis"[All Fields]) OR (("teeth s"[All Fields] OR "teeths"[All Fields] OR "tooth"[MeSH Terms] OR "tooth"[All Fields] OR "teeth"[All Fields] OR "tooth s"[All Fields] OR "tooths"[All Fields]) AND ("drug implants"[MeSH Terms] OR ("drug"[All Fields] AND "implants"[All Fields]) OR "drug implants"[All Fields] OR "implant"[All Fields] OR "embryo implantation"[MeSH Terms] OR ("embryo"[All Fields] AND "implantation"[All Fields]) OR "embryo implantation"[All Fields] OR "implantation"[All Fields] OR "implant s"[All Fields] OR "implantability"[All Fields] OR "implantable"[All Fields] OR "implantables"[All Fields] OR "implantate"[All Fields] OR "implantated"[All Fields] OR "implantates"[All Fields] OR "implantations"[All Fields] OR "implanted"[All Fields] OR "implanter"[All Fields] OR "implanters"[All Fields] OR "implanting"[All Fields] OR "implantion"[All Fields] OR "implantitis"[All Fields] OR "implants"[All Fields]))) AND ("head and neck neoplasms"[MeSH Terms] OR ("head"[All Fields] AND "neck"[All Fields] AND "neoplasms"[All Fields]) OR "head and neck neoplasms"[All Fields] OR ("head"[All Fields] AND "neck"[All Fields] AND "cancer"[All Fields]) OR "head and neck cancer"[All Fields] OR (("head neck"[Journal] OR ("head"[All Fields] AND "and"[All Fields] AND "neck"[All Fields]) OR "head and neck"[All Fields]) AND ("cysts"[MeSH Terms] OR "cysts"[All Fields] OR "cyst"[All Fields] OR "neurofibroma"[MeSH Terms] OR "neurofibroma"[All Fields] OR "neurofibromas"[All Fields] OR "tumor s"[All Fields] OR "tumoral"[All Fields] OR "tumorous"[All Fields] OR "tumour"[All Fields] OR "neoplasms"[MeSH Terms] OR "neoplasms"[All Fields] OR "tumor"[All Fields] OR "tumour s"[All Fields] OR "tumoural"[All Fields] OR "tumourous"[All Fields] OR "tumours"[All Fields] OR "tumors"[All Fields])) OR ("head and neck neoplasms"[MeSH Terms] OR ("head"[All Fields] AND "neck"[All Fields] AND "neoplasms"[All Fields]) OR "head and neck neoplasms"[All Fields]) OR ("head neck oncol"[Journal] OR ("head"[All Fields] AND "and"[All Fields] AND "neck"[All Fields] AND "oncology"[All Fields]) OR "head and neck oncology"[All Fields]) OR ("mouth neoplasms"[MeSH Terms] OR ("mouth"[All Fields] AND "neoplasms"[All Fields]) OR "mouth neoplasms"[All Fields]) OR ("mouth neoplasms"[MeSH Terms] OR ("mouth"[All Fields] AND "neoplasms"[All Fields]) OR "mouth neoplasms"[All Fields] OR ("oral"[All Fields] AND "cancer"[All Fields]) OR "oral cancer"[All Fields])) AND ("radiotherapy"[MeSH Subheading] OR "radiotherapy"[All Fields] OR ("radiation"[All Fields] AND "therapy"[All Fields]) OR "radiation therapy"[All Fields] OR "radiotherapy"[MeSH Terms] OR ("radiation"[All Fields] AND "therapy"[All Fields]) OR "radiation therapy"[All

	Fields] OR ("radiotherapy"[MeSH Terms] OR "radiotherapy"[All Fields] OR ("radiation"[All Fields] AND "therapies"[All Fields]) OR "radiation therapies"[All Fields]) OR ("radiotherapy"[MeSH Terms] OR "radiotherapy"[All Fields] OR "radiotherapies"[All Fields] OR "radiotherapy"[MeSH Subheading] OR "radiotherapy s"[All Fields]) OR ("radiotherapy"[MeSH Terms] OR "radiotherapy"[All Fields] OR "radiotherapies"[All Fields] OR "radiotherapy"[MeSH Subheading] OR "radiotherapy s"[All Fields]) OR ("irradiance"[All Fields] OR "irradiances"[All Fields] OR "irradiant"[All Fields] OR "irradiates"[All Fields] OR "irradiating"[All Fields] OR "irradiations"[All Fields] OR "irradiative"[All Fields] OR "irradiator"[All Fields] OR "irradiators"[All Fields] OR "radiotherapy"[MeSH Terms] OR "radiotherapy"[All Fields] OR "irradiate"[All Fields] OR "irradiated"[All Fields] OR "irradiation"[All Fields]) OR (("irradiance"[All Fields] OR "irradiances"[All Fields] OR "irradiant"[All Fields] OR "irradiates"[All Fields] OR "irradiating"[All Fields] OR "irradiations"[All Fields] OR "irradiative"[All Fields] OR "irradiator"[All Fields] OR "irradiators"[All Fields] OR "radiotherapy"[MeSH Terms] OR "radiotherapy"[All Fields] OR "irradiate"[All Fields] OR "irradiated"[All Fields] OR "irradiation"[All Fields]) AND ("patient s"[All Fields] OR "patients"[MeSH Terms] OR "patients"[All Fields] OR "patient"[All Fields] OR "patients s"[All Fields])))
Embase	((('dental'/exp OR dental) AND ('implants'/exp OR implants)) OR (('dental'/exp OR dental) AND ('implant'/exp OR implant)) OR (('dental'/exp OR dental) AND ('implantation'/exp OR implantation)) OR ('implant supported' AND ('dental'/exp OR dental) AND ('prosthesis'/exp OR prosthesis)) OR (endosseous AND ('dental'/exp OR dental) AND ('implantation'/exp OR implantation)) OR (('dental'/exp OR dental) AND ('implant'/exp OR implant) AND ('survival'/exp OR survival)) OR (surgical AND ('dental'/exp OR dental) AND ('prostheses'/exp OR prostheses)) OR (surgical AND ('dental'/exp OR dental) AND ('prosthesis'/exp OR prosthesis)) OR (('tooth'/exp OR tooth) AND ('implant'/exp OR implant))) AND ((('head'/exp OR head) AND ('neck'/exp OR neck) AND ('cancer'/exp OR cancer)) OR (('head'/exp OR head) AND ('neck'/exp OR neck) AND ('tumors'/exp OR tumors)) OR (('head'/exp OR head) AND ('neck'/exp OR neck) AND ('neoplasms'/exp OR neoplasms)) OR (('head'/exp OR head) AND ('neck'/exp OR neck) AND ('oncology'/exp OR oncology)) OR (('mouth'/exp OR mouth) AND ('neoplasms'/exp OR neoplasms)) OR (oral AND ('cancer'/exp OR cancer))) AND ((('radiation'/exp OR radiation) AND ('therapy'/exp OR therapy)) OR (('radiation'/exp OR radiation) AND therapies) OR ('radiotherapy'/exp OR radiotherapy) OR radiotherapies OR ('irradiation'/exp OR irradiation) OR (('irradiation'/exp OR irradiation) AND ('patients'/exp OR patients)))
Cochrane Library	dental implants OR dental implant OR dental implantation OR implant-supported dental prosthesis OR endosseous dental implantation OR dental implant survival OR surgical dental prostheses OR surgical dental prosthesis OR tooth implant AND head and neck cancer OR head and neck tumors OR head and neck neoplasms OR head and neck Oncology OR mouth neoplasms OR oral cancer AND radiation therapy OR radiation therapies OR radiotherapy OR radiotherapies OR irradiation OR irradiation patients
Web of Science	((((((ALL=(dental implants OR dental implant OR dental implantation OR implant-supported dental prosthesis OR endosseous dental implantation OR dental implant survival OR surgical dental prostheses OR surgical dental prosthesis OR tooth implant AND head and neck cancer OR head and neck tumors OR head and neck neoplasms OR head and neck Oncology OR mouth neoplasms OR oral cancer AND radiation therapy OR radiation therapies OR radiotherapy OR radiotherapies OR irradiation OR irradiation patients)) AND ALL=(dental implant)) AND ALL=(radiotherapy)) AND ALL=(head and neck cancer)) AND ALL=(dental implants)) AND ALL=(radiotherapy)) AND ALL=(head and neck cancer)
Google Scholar	dental implants OR dental implant OR dental implantation OR implant-supported dental prosthesis OR endosseous dental implantation OR dental implant survival OR surgical dental prostheses OR surgical dental prosthesis OR tooth implant AND head and neck cancer OR head and neck tumors OR head and neck neoplasms OR head and neck Oncology OR mouth neoplasms OR oral cancer AND radiation therapy OR radiation therapies OR radiotherapy OR radiotherapies OR irradiation OR irradiation patients

S 2 – Primary studies included in systematic reviews

Study	Study design	Number of implants placed	Number of implants failed	Fallow-up, months	Implant Survival rate

Pieralli et al. (2021)	RS	Overall 322<br>Mx 128<br>Md 194<br>HCN 217<br>RT 113<br>NRT 104<br>GB 47<br>NB 170	NM	81.2 ± 50.3	Control group 100%<br>HCN 98.2%<br>RT 98.2%<br>NRT 98.1%<br>GB 97.9%<br>Irrad. GB 95.2%<br>N-irrad.NB 100%<br>NB 98.2%<br>Irrad NB 98.9%<br>N-irrad NB 97.4%
Neckel et al. (2020)	PS	Irrad NB 81<br>Mx 26<br>Md 55	NM	36	Overvall 97.5
Ettl et al. (2020) -results of previus publication Ettl et al. (2016)	PS	Overall 234<br>RT 177<br>NRT 57<br>Mx 92<br>Md 142<br>GB 42<br>NB 192	NM	24	Overall<br>2 years 92.3%<br>1 year 94.4%<br>Implant success overall<br>2 years 78.6%<br>1 year 86.3%<br>Mx 73.9%<br>Md 81.7%<br>NB 81.2%<br>GB 69.1%<br>RT 76.7%<br>Inside PTV 76.2%<br>Outside PTV 77%<br>NRT 86%
Patel et al. (2020)	RS	Overall 376<br>RT 132<br>NRT 244<br>Mx 99<br>Md 277<br>GB 43<br>NB 333<br>14 Zygoma implantS	NM	46,92 (1,32 – 152,12)	Without Zigoma implants<br>Overal 97.5%<br>NB 98.1%<br>Irrad NB 97.3%<br>N-irrad NB 98.6%<br>GB 93%<br>Irrad GB 88.9%<br>N-irrad GB 94.1%<br>RT 96.7%<br>NRT 97.9%
Albrega et al. (2020)	PS	58<br>RT 42<br>NRT 16	NM	Median 18,5	Overall: 93.1%<br>RT 90.5%<br>NRT 100%
Sandoval et al. (2020)	RS	29<br>All implants placed in irradiated fibula free flaps (postop RT)	NM	From surgery 7 (3–14)<br>From RT completion 3.5 (0–11)	93.1%
Di Carlo et al. (2019)	RS	84<br>Mx 36<br>Md 48<br>All in irradiated residual bone	NM	After implantation 22.9 (SD 15.5)<br>After RT 39.5 (SD 22.8)	Overall: 90.5%<br>Mx 94.4%<br>Ant 92.3%<br>Post 95.7%<br>Md 87.5%<br>Ant 100%<br>Post 82.4%
Woods et al. (2019)	RS	102<br>RT 51<br>NRT 51<br>GB 10<br>NB 92<br>Mx and md<br>Immediate 39<br>Delayed 63	NM	23 (range 2–140)	Overall 93.1%<br>RT 90.2%<br>NRT 96.1%<br>GB 100%<br>NB 92.4%

Laverty et al. (2019)	RS	779<br>Mx 373<br>Md 406<br>GB 112<br>NB 650<br>NB+ALT e radial 667<br>RT 382<br>RCT: 143<br>RT/RCT 525<br>NRT 254<br>Placement primary 26<br>Delayed 753	NM	Mean 43 (range 1–142)<br>Median 38	Overall 95.6%<br>RT/RCT 95%<br>RT 96.1%<br>RCT 92.3%<br>NRT 96.9%<br>NB 98.2%<br>NB+ALT and radial flap 97.8%<br>GB 83%<br>scapula 100%<br>fíbula 83.1%<br>DCIA 76.0%<br>Iliac crest (non-vascular) 80%
Moore et al. (2019)	RS	Dental implant 78<br>GB 38<br>NB 40<br>RT 63<br>NRT 15	NM	25.7 (6–89)	Dental implant<br>Overall 85.9%<br>NB 87.5%<br>GB 84.2%<br>RT 82.5%<br>NRT 100%
Papi et al. (2019)	PS	113<br>Mx 35<br>Md 78<br>Irradiated residual bone	NM	Range 24–30 after prosthetic rehabilitati on	Overall 94.7%<br>Mx 91.4%<br>Md 96.2%
Curi et al. (2018)	RS	169<br>Mx 79<br>Md 90<br>All in irradiated bone	NM	89,6 (3.6 – 176.4)	Overall: 92.9%<br>Mx 91.1%<br>Md 94.4%<br>5 years success rate<br>Mx 92.4%<br>Md 90.9%
Flores-Ruiz et al. (2018)	RS	106<br>Mx 43<br>Md 63<br>GB 15 (Mx 7, Md 8)<br>NB 91 (36 Mx, Md 55)<br>RT 78<br>NRT 28	NM<br>13	60	Overall 87.7%<br>Mx 79.1%<br>Md 93.7%<br>GB 73.3%<br>Irrad GB 71.4%<br>N-irrad GB 75%<br>NB 90.1%<br>Irrad NB 87.3%<br>N-irrad NB 100%<br>RT/RCT 85.9%<br>NRT 92.9%
Menapace et al. (2018)	RS	121	NM	NM	NM
Pellagrino et al. (2018)	RS	108	NM	NM	NM
Sozzi et al. (2017)	RS	FFF 100<br>RT 26<br>NRT 74	NM	NM	General Sucess Rate<br>97,9% (60 months)<br>72,8% (120 months)
Burgess et al. (2017)	RS	199<br>FF 96<br>DCIA 64<br>Scapula 37<br>Radius 2<br>RT 45<br>NRT 154<br>All in GB	NM	24 (6-60) from surgery	Overall 94.5%<br>RT 84.4%<br>NRT 97.4%<br>FF 91.7%<br>DCIA 96.9%<br>Scapula 97.3%<br>Radius 100%
Woods et al. (2017)	RS	156	NM	NM	NM
Wu et al. (2016)	RS	198	NM	NM	93.6% after 10 years

Ettl et al. (2016)	PS	309<br>Implants irrad bone	NM	12	95.2% (irrad bone)
Rana et al. (2016)	RS	162<br>Mx 70 (ant 35, post 35)<br>Md 92 (ant 52, post 40) Irrad bone	NM	60	Overall 67.9%<br>Mx 71.4%<br>Ant 65.7%<br>Post 77.1%<br>Md 65.2%<br>Ant 65.4%<br>Post 65%
Ernest et al. (2016)	RS	194<br>Mx 73<br>Md 121<br>GB 12<br>NB 182<br>RT 88<br>NRT 106	NM	52,92 (24 – 117)	Overall 97.9%<br>Mx:100%<br>Md 96.7%<br>GB (n-irrad) 100%<br>NB 97.8%<br>Irrad NB 96.6%<br>N-irrad NB 98.9%<br>RT: 96.6%<br>NRT: 99.1%
Barber et al. (2016)	RS	82<br>FFF 35 (RT 12, NRT 23)<br>BIFFF 47 (RT 13, NRT 34)<br>RT 25<br>NRT 57<br>All GB	NM	60	Overall 87.8%<br>RT 84%<br>NRT 89.5%<br>FFF 77.1%<br>RT FFF 83.3%<br>NRT FFF 73.9%<br>BIFFF 95.7%<br>RT BIFFF 84.6%<br>NRT BIFFF 100%<br>>10 packyears smokers 93.1%
Ch'ng et al. (2016)	RS	1132<br>NB 889<br>Mx 271<br>Md 618<br>GB (FFF) 243<br>RT 795<br>preop 100<br>postop 695<br>NRT 337	NM	Median 33,7 (0,9 – 92,7)	Overall 96.3%<br>3 years 92.8%, 5 years 92.2%)<br>Mx 97.8%<br>Md 97.4%<br>GB (FFF) 91.8%<br>Irrad GB 83.3%<br>Non-irrad GB 94.9%<br>NB 97.5%<br>Irrad NB 97.4%<br>N-irrad NB 98.1%<br>RT 96.2%<br>preop RT 92%<br>postop RT 96.8%<br>NRT 96.4%
Jackson et al. (2016)	RS	15	NM	NM	NM
Kobayashi et al. (2016)	RS	134	NM	NM	NM
Wetzels et al. (2016)	PS	NR	NM	60	NM
Wetzels et al. (2017)	RS	NR	NM	60	NM
Pompa et al. (2015) – patient data matches partly with Di Carlo et al. (2019)	RS	168<br>Mx 72 (26 ant, 46 post)<br>Md 96 (28 ant, 68 post)<br>RT 51<br>NRT 117	NM	After RT 39.5 SD 22.8<br>After implant placement 22.9 SD 15.5	Overall 90.5%<br>Mx 94.4%<br>Md 87.5%<br>RCT 76.5%<br>NRT 96.6%<br>Mx (ant 92.3%, post 95.7%)<br>Md (ant 100%, post 82.4%)

Hessling et al. (2015)	RS	272<br>Mx 83<br>Md 189<br>NB 179<br>GB 93<br>RCT 223<br>(neoadjuvant 95, adjuvante 128)<br>NRT 49	NM	30.9 (3 – 82)	Overall<br>2 years 98.9%,<br>5 years 97.1%<br>During observation time (≤82 meses) 96.3%<br>After 5 years<br>GB 94,6%<br>NB 98.3%<br>RCT 96.4%<br>Neoadjuvant 97.9%<br>Adjuvant 95.3%<br>NRT 100%
Nack et al. (2015) – 5 years follow-up of a previus publication Heberer et al. (2011)	RS	97 (SLA 48, SLActive 49)<br>(102 at beginning, 50 SLA, 52 SLActive)<br>Mx 47<br>Md 35<br>Irrad bone<br>(Mx 55; Md 47 at beginning)	NM	60 (36 – 72)	Overall (after 5 years)<br>79.4%<br>SLA 79.2%<br>SLActive 79.6%
Doll et al. (2015) – based partly on data published by Nelson et al. (2007)	RS	830<br>Mx 450<br>Md 380<br>RT 292 (Mx 74, Md 118)<br>NRT 538	NM	121 (37 – 240)	Overall 92.2%<br>Mx 92%<br>Md 92.4%<br>NRT 93.5%<br>RT 89.7%
Hakim et al. (2015)	RS	119<br>Mx and Md<br>All in GB (FFF)<br>RT 48<br>NRT 71	NM	94,5 ± 37,3 (3 – 172)	Overall 92.4%<br>RT 89.6%<br>NRT 94.4%
Saracoglu et al. (2015)	PS	Irrad 40<br>N-irrad 40<br>Mx 40<br>Md 40	NM	12	Mx p<0.002<br>Md p<0.001
Jacobsen et al. (2014)	RS	140<br>RT 47<br>NRT 93<br>GB 99 (Irrad 13, N-irrad 86)<br>NB 41 (Irrad 34, N-irrad 7)	RT 14<br>NRT 13<br>GB 13<br>NB 6<br>12 N-irrad GB<br>1 N-irrad NB	67	Overall 80.7%<br>1 year 93.6%<br>5 years 83.3%<br>RT 70.2%<br>NRT 86%<br>GB 79.8%<br>Irrad GB 38.5%<br>N-irrad GB 86%<br>NB 85.4%<br>Irra NB 82.4%<br>N-irrad NB 85.7%
Korfage et al. (2014)	PS	524<br>RT 318<br>NRT 206<br>Native md	NM	45.6 (0 – 174)	Overall 93.1%<br>RT 90.3%<br>NRT 97.6%
Gander et al. (2014)	RS	136<br>GB 48<br>NB 88<br>RT 84<br>NRT 52<br>All in md	NM	20	Overall<br>After 20 months 87.5%<br>After 12 months 92.7%<br>GB 87.5%<br>NB 87.5%<br>RT 85.7%<br>NRT 90.4%

Dholam et al. (2013)	PS	85<br>GB 40<br>NB 45<br>RT 59<br>NRT 26	NM	60	Survival rate NM<br>Rate of osseointegration<br>Overall 88%<br>GB 93%<br>NB 85%<br>RT 83%<br>NRT 100%<br>Success rate<br>Overall 76.5%<br>GB 72.5%<br>NB 80%<br>RT 71.2%<br>NRT 88.5%
Buurman et al. (2013)	RS	73<br>Md irrad	NM	48.6 (14 – 132)	97.3%<br>Success rate 95.9%
Fierz et al. (2013)	RS	104<br>Mx 28<br>Md 76<br>GB 46<br>NB 58<br>RT 62<br>NRT 42	NM	36 – 72	Overall 82.7%<br>GB 82.6%<br>Irrad GB 70%<br>N-irrad GB 92.3%<br>NB 82.8%<br>Irrad NB 81%<br>N-irrad NB 87.5%<br>RT 77.4%<br>NRT 90.5%
Katsoulis et al. (2013)	RS	104<br>RT 64<br>NRT 42<br>Mx 24<br>Md 80<br>Irrad GB 20<br>Irrad NB 42<br>N-irrad GB 26<br>N-irrad NB 16	18<br>RT 14<br>NRT 4<br>Mx 4<br>Md 10<br>Irrad GB 6<br>Irrad NB 8<br>N-irrad GB 2<br>N-irrad NB 2	24 - 60	Overall 86.53%<br>RT 77.42%<br>NRT 90.48%<br>Mx 83.33%<br>Md 87.5%<br>Irrad GB 70%<br>Irrad NB 80.95%<br>N-irrad NB 92.31%<br>N-irrad NB 87.5%
Mizbah et al. (2013)	RS	NM	NM	60	NM
Buddula et al. (2012)	RS	271	NM	60	Overall 89.9%<br>Mx 80.5%<br>Md 93.6%<br>NB 93.4%<br>GB 83.3%
Mancha de La Plata et al. (2012)	RS	355<br>RT 225<br>NRT 130<br>Irrad Mx 94<br>Irrad Md 131	28<br>RT 22<br>NRT 6<br>Irrad Mx 7<br>Irrad Md 15	45 (6 – 96)	*Overall 91.64<br>RT 90.22%<br>NRT 95.38%<br>Irrad Mx 92.55%<br>Irrad Md 88.55%
Linsen et al. (2012)	RS	262<br>RT 127<br>NRT 135	14<br>RT 8<br>NRT 6	42	Overall 86.9% (10 years)<br>RT 95.6% (10 years)<br>RT and chemotherapy 91.5% (5 years)<br>NRT 84.7% (10 years)
Fenlon et al. (2012)	**RS/TS	145<br>RT 35<br>NRT 110	18<br>RT 15<br>NRT 3	36	(1) Overall 87.58%<br>RT 57%<br>NRT 97%<br>Immediate placement 35%<br>Non-immediate 96%
Al-Nawas et al. (2012)	RS	516<br>RT 87<br>NRT 429	RT 2<br>NRT 7	120	NM

Buddula et al. (2011)	RS	271<br>GB 118<br>NB 212	NM	23 (5 – 203)<br>NB/GB 36	Turned implants Mx 72.6% (5 years)<br>Turned implant Md 91.7% (5 years)<br>Roughened implants Mx 87.5% (5 years)<br>Roughened implants Md 100% (5 years)<br>GB Mx 82.3%<br>GB Md 98.1%,<br>NB Mx 79.8%<br>NB Md 100%
Buddula et al. (2011)	RS	Irrad Mx 62<br>Irrad Md 209<br>Irrad GB 59<br>Irrad NB 212	Irrad Mx 20<br>Irrad Md 13<br>Irrad GB 8<br>Irrad NB 25	144	Irrad Mx 67.64%<br>Irrad Md 93.78%<br>Irrad GB 86.14%<br>Irrad NB 88.21%
Bodard et al. (2011)	RS	Irrad GB 75	Irrad GB 6	27,5	Irrad. GB 80%
Sammartino et al. (2011)	**CCT/OS	188<br>Irrad Mx 42<br>Irrad Md 130<br><12 127<br>≥12 61	NR<br>Irrad Mx 18<br>Irrad Md 2<br><12 12<br>≥12 8	36	Overall 89.4%<br>Irrad Mx 57.1%<br>Irrad Md 98.4%<br>>50 Gy 78.6%<br>< 50 Gy 93.6%<br><12 months 91%<br>≥12 months 87%
Heberer et al. (2011) – final results reported by Nack et al. (2015)	**PS/RCT /CCT	102<br>SLA 50<br>SLActive 52<br>Irrad Mx 55<br>Irrad Md 42	Irrad Mx 0<br>Irrad Md 2	14.4 (12 – 26)	SLA 96%<br>SLActive 100%<br>Irrad Mx 100%<br>Irrad Md 95.24%
Salinas et al. (2011)	RS	206<br>RT 90<br>NRT 116	31<br>RT 23<br>NRT 8	41.1 (4 – 108)	Overall 85%<br>RT 74.4%<br>NRT 93.1%
					Irrad GB 72.5%<br>N-irrad GB 90.4%<br>Irrad. NB 76.9%<br>N-irrad NB 96.2%
Barrowman et al. (2011)	RS	*115<br>RT 48<br>NRT 67<br>Mx 35<br>Md 80<br>N-irrad Mx NB 9	*5<br>RT 5<br>NRT 0<br>Mx 0<br>Md 5<br>N-irrad NB Mx 0	192	*95.65% Overall<br>89.58% RT<br>100% NRT<br>100% Mx<br>93.75% Md
Korfage et al. (2010)	**CCT/PS	195<br>RT 123<br>NRT 72	14<br>RT 13<br>NRT 1	60	*Overall 92.82%<br>RT 89.4%<br>NRT 98.6%
Klein et al. (2009)	RS	190<br>RT 116<br>NRT 74<br><50 Gy 55<br>≥50 Gy 61	25<br>RT 13<br>NRT 12<br><50 Gy 4<br>≥50 Gy 9	60	Overall 86.8%<br>RT 88.8%<br>≥50 Gy 85.2%<br><50 Gy 92.7%<br>NRT 83.8%<br>Irrad GB 89.2%<br>N-irrad GB 68.6%<br>Irrad NB 97.4%<br>N-irrad NB 91.3%<br><50 Gy 92.73%<br>≥50 Gy 85.25%
Cuesta-Gil et al. (2009)	RS	706<br>RT 395<br>NRT 311	81<br>RT 75<br>NRT 6	72 - 108	92.9%<br>RT 81.01%<br>NRT 98.07%

Schoen et al. (2008)	PS	186<br>RT 124<br>NRT 62	NM	18 - 24	RT NB 97%<br>NRT NB 97%
Alsaadi et al. (2008)	RS	RT 15<br>NRT 1499	RT 3<br>NRT 98		RT 80%<br>NRT 93.46%
Nelson et al. (2007)	RS	435<br>RT 124<br>NRT 311	11<br>RT 7<br>NRT 4	120 (5 – 161)	*Overall 97.47%<br>RT 94.35%<br>NRT 98.71%<br>Overall 70% (8 years)<br>Overall 69% (13 years)<br>RT 84% (3.8 years)<br>RT 54% (13.5 years)
Schoen et al. (2007)	**CCT/RCT	103<br>HBO 54<br>N-HBO 49	11<br>HBO 8<br>N-HBO 3	12	*Overall 89.32%<br>HBO 85.19%<br>N-HBO 93.88%
Yerit et al. (2006)	RS	316<br>RT 154<br>NRT 162<br>Irrad NB 154<br>N- irrad GB 78<br>N-irrad NB 84<br><12 months 143<br>>12 months 173	44<br>RT 29<br>NRT 15<br>Irrad NB 29<br>N- irrad GB 13<br>N-irrad NB 2<br><12 months 29<br>>12 months 15	60 (4 – 151)	RT 81.17%<br>NRT 90.70%<br>Irrad NB 81.17%<br>N- irrad GB 83.33%<br>N-irrad NB 97.62%<br><12 months 79.72%<br>>12 months 91.33%
Schepers et al. (2006)	RS	139<br>RT 61<br>NRT 78	2<br>RT 2<br>NRT 0	96	*Overall 98.56%<br>RT 97%<br>NRT 100%
Landés and Kovács (2006)	PS	114<br>72 RT 72<br>42 NRT 4	1<br>1 RT 1<br>0 NRT 0	Mean 36 (24 – 46)	*Overall 99,12%<br>98.61% RT<br>100% NRT
Bodard et al. (2006)	RS	68<br>6 Irrad Mx 6<br>62 Irrad Md 62	0 Irrad Mx 0<br>0 Irrad Md 0	Mean 31.9	*Overall 100%<br>100% Irrad Mx<br>100% Irrad Md
Chispasco et al. (2006)	RS	71	NM	NM	NM
Teoh et al. (2005)	RS	102<br>RT 30<br>NRT 72<br>HBO 15<br>N-HBO 15	6<br>RT 5<br>NRT 1<br>HBO 5<br>N-HBO 0	Mean 51.7 (1.3 – 138)	*Overall 94.11%<br>RT 83.33%<br>NRT 98.61%<br>HBO 66.67%<br>N-HBO 100%
Shaw et al. (2005)	RS	364<br>RT 172<br>NRT 192<br>GB 123<br>NB 241<br>HBO 77<br>N-HBO 95<br>Irrad Md 98<br>N-irrad Md 110<br><50Gy 44<br>=50Gy 78<br>>50Gy 33<br>Mx 42<br>Md 199	56<br>RT 31<br>NRT 25<br>GB 32<br>NB 24<br>HBO 15<br>N-HBO 17<br>Irrad Md 1<br>N-irrad Md 15<br><50 Gy 11<br>=50 Gy 12<br>>50 Gy 8<br>Mx 8<br>Md 16	Mean 3.5 years (0.3 – 14)	*Overall 84.61%<br>RT 81.98%<br>NRT 86.98%<br>GB 73.98%<br>NB 90.04%<br>HBO 85.71%<br>N-HBO 82.11%<br>Md 98.88%<br>N-irrad Md 86.36%<br><50 Gy 75%<br>=50 Gy 84.62%<br>>50 Gy 75.76%<br>Mx 80.95%<br>Md 91.96%
Granström (2005)	RS	1245<br>RT 631<br>RT 614<br>HBO 340<br>N-HBO 291	223<br>RT 147<br>NRT 76<br>HBO 29<br>N-HBO 117	72 (6 – 276)	*Overall 82.08%<br>RT 75%<br>NRT 87%<br>HBO 91.47%<br>N-HBO 59.79%

Granström (2003)	RS	206<br>HBO 133<br>N-HBO 73<br>Irrad Mx 109<br>Irrad Md 97<br>Irrad GB 40<br>Irrad NB 14	22<br>HBO 5<br>N-HBO 17<br>Irrad Mx 14<br>Irrad Md 8<br>Irrad GB 0<br>Irrad NB 2	Mean 9.8 years (2.2 – 22)	*Geral 89.32%<br>HBO 96.2%<br>N-HBO 76.7%<br>Irrad Mx 87.2%<br>Irrad Md 91.8%<br>Irrad GB 100%<br>Irrad NB 100%
Cao et al. (2003)	RS	131<br>RT 53<br>NRT 78	29<br>RT 18<br>NRT 11	60	Overall 65%<br>RT 49.44%<br>NRT 77.8%
Schoen et al. (2003)	PS	NM	NM	13 - 40	NM
Visch et al. (2002)	PS	446<br>Irrad Mx 108<br>Irrad Md 338<br><50 Gy 207<br>≥50 Gy 239<br><12 months 175<br>≥12 months 271	64<br>Irrad Mx 33<br>Irrad Md 31<br><50 Gy 19<br>≥50 Gy 45<br><12 months 29<br>≥12 months 35	120	Overall 78%<br>Irrad Mx 88.04%<br>Irrad Md 95.45%<br><50 Gy 90,82%<br>≥50 Gy 81.18%<br><12 months 94.12%<br>≥12 months 90.18%
Van Steen-berghe et al. (2002)	CCT	1266<br>RT 33<br>NRT 1230	27<br>RT 2<br>NRT 25	Mean 1881 days (72 – 3901)	*Overall 97,86%<br>RT 93.94%<br>NRT 97.97%
Goto et al. (2002)	RS	180<br>RT 92<br>NRT 88<br>Mx 52<br>Md 128<br>Irrad GB 68<br>Irrad NB 112	15<br>RT 11<br>NRT 4<br>Mx 11<br>Md 4<br>Irrad GB 4<br>Irrad NB 11	2 - 130	*Overall 91.66%<br>RT 88.04%<br>NRT 95.45%<br>Mx 78.85%<br>Md 96.87%<br>Irrad GB 94.14%<br>Irrad NB 90.18%
Grötz et al. (1999)	RS	197	NM	72	72%
Betz et al. (1999)	PS	261	NM	NM	77,8%
Weischer and Mohr (1999)	RS	175<br>RT 83<br>NRT 92	15<br>10 (GRT)<br>5 (GNRT)	37	Overall 91% (3 years)<br>GRT 87.95%<br>GNRT 94.57%<br>RT 75% (7 years)<br>NRT 86% (10 years)
Schliephake et al. (1999)	RS	409<br>RT 145<br>NRT 264	NM	120	Overall 56,6%<br>RT 49.8%<br>NRT 57.7%
Werkmeister et al. (1999)	RS	109<br>RT 30<br>NRT 79<br>N-irrad GB 45<br>Irrad NB 30<br>N-irrad NB 34<br>>54 Gy 16<br><54 Gy 14	27<br>RT 8<br>NRT 19<br>N-irrad GB 14<br>Irrad NB 8<br>N-irrad NB 5<br>>54 Gy5<br><54 Gy 3	36	*Overall 75.22%<br>RT 73%<br>NRT 75.94%<br>N-irrad GB 68.89%<br>Irrad NB 73.33%<br>N-irrad NB 85.29%<br>>54 Gy) 68.75%<br><54 Gy 78.57%
Mericske-Stem et al. (1999)	CCT	53<br>RT 33<br>NRT 20<br>Mx 12<br>Md 41	8<br>RT 8<br>NRT 0<br>Mx 0<br>Md 8	37 (12 – 84)	*Overall 84.90%<br>RT 7.76%<br>NRT 100%<br>Mx 100%<br>Md 80.49%
Keller et al. (1999)	RS	248<br>RT 11<br>NRT 237	33<br>RT 0<br>NRT 33	120	*Overall 86.69%<br>RT 100%<br>NRT 86.08

Granström et al. (1999)	RS	335<br>RT 246<br>NRT 89<br>HBO 99<br>N-HBO 147	99<br>RT 87<br>NRT 12<br>HBO 8<br>N-HBO 79	44.2 – 88.8	*Overall 70.44%<br>RT64.63%<br>NRT 86.52%<br>HBO 91.92%<br>N-HBO 46.26%
Foster et al. (1999)	RS	104<br>Irrad GB 15<br>N-irrad GB 89	7<br>Irrad GB 0<br>N-irrad GB 7	132	*Overall 93.26<br>Irrad GB100%<br>N-irrad GB 92.13%
Esser et al. (1999)	RS	276<br>RT 148<br>NRT 128	12<br>RT 9<br>NRT 3	58.2	*Overall 95.65%<br>RT 93.92%<br>NRT 97.66%
Niimi et al. (1998)	RS	RT 228<br>Mx 59<br>Md 169	RT 20<br>Mx 17<br>Md 3	49	*Overall 91.22<br>Japan 88.9%<br>USA 86%
Ihara et al. (1998)	RS	74<br>RT39<br>NRT 35<br>HBO 19<br>N-HBO 20	15<br>RT 6<br>NRT 9<br>HBO 3<br>N-HBO 3	27.6	*Overall 79.711%<br>RT 84.62%<br>NRT 74.29%<br>HBO 84.21%<br>N-HBO 85%
Brogniez et al. (1998)	RS	53<br>Irrad Mx 3<br>Irrad Md 50	2<br>Irrad Mx 0<br>Irrad Md 2	38 (6 – 68)	*Overall 96.22%<br>Irrad Mx 100%<br>Irrad Md 96%
Andersson et al. (1998)	RS	90<br>Irrad Mx 12<br>Irrad Md 78	2<br>Irrad Mx 0<br>Irrad Md 2	96	*Overall 97.77%<br>Irrad Mx 100%<br>Irrad Md 97.44%
August et al. (1998)	RS	40<br>RT 38<br>NRT 2	0	16.4	100%
Wagner et al. (1998)	RS	275<br>RT 145<br>NRT 130	NM	65	Overall 97.9% (5 years)<br>Overall 72.8% (10 years)
Niimi et al. (1997)	RS	110<br>Irrad Mx 39<br>Irrad Md 71<br>HBO 31<br>N-HBO 79<br>≤45 Gy 67<br>>45 Gy 51	12<br>Irrad Mx 9<br>Irrad Md 3<br>HBO 4<br>N-HBO 8<br>≤45 Gy 7<br>>45 Gy 5	2 72	*Overall 89.09%<br>Irrad Mx 76.92%<br>Irrad Md 95.77%<br>HBO 87.1%<br>N-HBO 89.87%<br>≤45 Gy 89.55%<br>>45 Gy 90.2%
Keller et al. (1997)	RS	98<br>Irrad GB 26<br>Irrad NB 72	1<br>Irrad GB 1<br>Irrad NB 0	120	*Overall 98.97<br>Irrad GB 96.15%<br>Irrad NB 100%
Marker et al. (1997)	RS	RT 19<br>NRT 19	RT 0<br>NRT 0	Mean 14<br>(7 – 47)	RT 100%<br>NRT 100%
Chan et al. (1997)	RS	69<br>RT 23<br>NRT 46	4<br>RT 4<br>NRT 0	Mean 32<br>(6 – 84)	*Overall 94.20<br>RT 82.61%<br>NRT 100%
Ali et al. (1997)	RS	42<br>Irrad Mx 10<br>Irrad Md 32	6<br>Irrad Mx 6<br>Irrad Md 0	Mean 33<br>(11 – 64)	*Overall 85.71%<br>Irrad Mx 40<br>Irrad Md 100%
Esser and Wagner (1997)	RS	320<br>RT 249<br>NRT 71	46<br>RT 39<br>NRT 7	60	*Overall 85.62%<br>RT 84.34%<br>NRT 90.14%
Jisander et al. (1997)	**CCT/RS	103<br>Irrad Mx 38<br>Irrad Md 65	5<br>Irrad Mx 3<br>Irrad Md 2	21 (1 – 62)	*Overall 95.14%<br>Mx 92% (1 year)<br>Mn 97% (1 year)

Roumanas et al. (1997)	RS	80<br>RT 45<br>NRT 35<br>Irrad GB 39<br>N-irrad GB 32<br>Irrad NB 5<br>N-irrad NB 4	1<br>RT 0<br>NRT 1<br>Irrad GB 0<br>N-irrad GB 1<br>Irrad NB 0<br>N-irrad NB 0	1 – 49	*Overall 98.75%<br>RT 100%<br>NRT 90.1%<br>Irrad GB 100%<br>N-irrad GB 96.9%<br>Irrad NB 100%<br>N-irrad NB 100%
McGhee et al. (1997)	RS	26<br>RT 21<br>NRT 5<br>Irrad GB 9<br>Irrad NB 12<br>N-irrad GB 5	2<br>RT 3<br>NRT 0<br>Irrad GB 0<br>Irrad NB 2<br>N-irrad GB 0	3 years	*Overall 92.30%<br>RT 90.48%<br>NRT 100%<br>Irrad GB 100%<br>Irrad NB 83.33%<br>N-irrad GB 100%
Watzinger et al. (1996)	RS	138	NM	NM	GB 58.3%<br>NB 87.8%
Weischer et al. (1996)	**CCT/PS	105<br>RT 57<br>NRT 48	7<br>RT 4<br>NRT3	26	*Overall 93.33%<br>RT 92.98%<br>NRT 93.75
Eckert et al. (1996)	RS	111<br>Irrad Mx 22<br>Irrad Md 89	9<br>Irrad Mx 8<br>Irrad Md 1	12 years	*Overall 91.89%<br>Irrad Mx 63.64%<br>Irrad Md 98.88%
Aldegheri et al. (1996)	RS	19<br>Irrad Mx 6<br>Irrad Md 13	0<br>Irrad Mx 0<br>Irrad Md 0	Mean 2,7 years (1 – 4)	*Overall 100%<br>Irrad Mx 100%<br>Irrad Md 100%
Franzén et al. (1995)	RS	20<br><50Gy 13<br>≥50Gy 7	1<br><50Gy 0<br>≥50Gy 1	36 – 72	*Overall 95%<br><50Gy 100%<br>≥50Gy 85.71%
Sclaroff et al. (1994)	RS	114<br>RT 80<br>NRT 34	2<br>RT 0<br>NRT 2	3 years 2 months	*Overall 98,24%<br>RT 100%<br>NRT 94.1%
Albrektsson et al. (1988)	RS	8045<br>RT 49<br>NRT 7996<br>Irrad Mx 16<br>Irrad Md 33<br>N-irrad Mx 3089<br>N- irrad Md 4907	273<br>RT 3<br>NRT 270<br>Irrad Mx 3<br>Irrad RMd 0<br>N-irrad Mx 218<br>N-irrad Md 52	3 – 8 years	*Overall 96.60%<br>RT 93.88 %<br>NRT 93.88 %<br>Irrad Mx 81.25%<br>Irrad Md 100%<br>N-irrad Mx 92.94%<br>N-irrad Md 98.94%

Abbreviations: ALT: anterolateral thigh flap, ant: anterior; BIFFF: bone-impacted fibula free flap, CCT: clinical controlled trial, DCIA: deep circumflex iliac artery, FFF: fibula free flap,iado, GB: grafted bone, HBO: group hyperbaric oxygen therapy, HCN: head and neck cancer, Irrad GB: irradiated grafted bone group, Irrad NB: irradiated native bone group, Irrad Md: mandibular irradiated group, Irrad Mx: maxillary irradiated group, Md: mandible, Mx: maxila, NB: native bone, N-HBO:group without hyperbaric oxygen therapy, N-irrad GB: non-irradiated grafted bone group, N_irrad NB: non-irradiated native bone group, N-irrad Md: mandibular non-irradiated group, N-irrad Mx: maxillary non-irradiated group, NRT: non-irradiated group, post: posterior, PS:prospective study, RS: retrospective study, RCT: randomized controlled trial, RT: group of irradiated patients, SLA: sandblasted and acid etched and SLActive: modified sandblasted and acid etched.
* Data obtained by arithmetic calculation carried out by the authors.
** Different classification in systematic reviews.

Reference

Pieralli S, Spies BC, Schweppe F, Preissner S, Nelson K, Heiland M, Nahles S. Retrospective long-term clinical evaluation of implant-prosthetic rehabilitations after head and neck cancer therapy. Clin Oral Implants Res. 2021 Apr;32(4):470-486. doi: 10.1111/clr.13716. Epub 2021 Feb 7. PMID: 33501694. https://doi.org/10.1111/clr.13716

Neckel N, Wagendorf P, Sachse C, Stromberger C, Vach K, Heiland M, Nahles S.

Influence of implant-specific radiation doses on peri-implant hard and soft tissue: An observational pilot study. Clin Oral Implants Res. 2021 Feb;32(2):249-261. doi: 10.1111/clr.13696. Epub 2020 Dec 21. PMID: 33278849. https://doi.org/10.1111/clr.13696

Ettl T, Junold N, Zeman F, Hautmann M, Hahnel S, Kolbeck C, Müller S, Klingelhöffer C, Reichert TE, Meier JK. Implant survival or implant success? Evaluation of implant-based prosthetic rehabilitation in head and neck cancer patients-a prospective

observational study. Clin Oral Investig. 2020 Sep;24(9):3039-3047. doi: 10.1007/s00784-019-03172-9. Epub 2019 Dec 12. PMID: 31832793.

Patel J, Antov H, Nixon P. Implant-supported oral rehabilitation in oncology patients: a retrospective cohort study. Br J Oral Maxillofac Surg. 2020 Oct;58(8):1003-1007. doi: 10.1016/j.bjoms.2020.05.016. Epub 2020 May 27. PMID: 32474015. https://doi.org/10.1016/j.bjoms.2020.05.016

Alberga JM, Korfage A, Bonnema I, Witjes MJH, Vissink A, Raghoebar GM. Mandibular dental implant placement immediately after teeth removal in head and neck cancer patients. Support Care Cancer. 2020 Dec;28(12):5911-5918. doi: 10.1007/s00520-020-05431-y. Epub 2020 Apr 11. PMID: 32279135; PMCID: PMC7686200. https://doi.org/10.1007/s00520-020-05431-y

Sandoval ML, Rosen EB, Robert AJ, Nelson JA, Matros E, Gelblum DY. Immediate dental implants in fibula free flaps to reconstruct the mandible: A pilot study of the short-term effects on radiotherapy for patients with head and neck cancer. Clin Implant Dent Relat Res. 2020 Feb;22(1):91-95. doi: 10.1111/cid.12870. Epub 2019 Nov 28. PMID: 31777154; PMCID: PMC9348905.

Di Carlo S, De Angelis F, Ciolfi A, Quarato A, Piccoli L, Pompa G, Brauner E. Timing for implant placement in patients treated with radiotherapy of head and neck. Clin Ter. 2019 Sep-Oct;170(5):e345-e351. doi: 10.7417/CT.2019.2153. PMID: 31612191. https://doi.org/10.7417/CT.2019.2153

Woods B, Schenberg M, Chandu A. A Comparison of Immediate and Delayed Dental Implant Placement in Head and Neck Surgery Patients. J Oral Maxillofac Surg. 2019 Jun;77(6):1156-1164. doi: 10.1016/j.joms.2019.02.007. Epub 2019 Feb 13. PMID: 30851250.

Laverty DP, Addison O, Wubie BA, Heo G, Parmar S, Martin T, Praveen P, Pearson D, Newsum D, Murphy M, Bateman G. Outcomes of implant-based oral rehabilitation in head and neck oncology patients-a retrospective evaluation of a large, single regional service cohort. Int J Implant Dent. 2019 Mar 5;5(1):8. doi: 10.1186/s40729-019-0161-y. PMID: 30834461; PMCID: PMC6399356.

Moore P, Grinsell D, Lyons B, Hewson I. Outcomes of dental and craniofacial osseointegrated implantation in head and neck cancer patients. Head Neck. 2019 Sep;41(9):3290-3298. doi: 10.1002/hed.25845. Epub 2019 Jun 19. PMID: 31215724.

Papi P, Brauner E, Di Carlo S, Musio D, Tombolini M, De Angelis F, Valentini V, Tombolini V, Polimeni A, Pompa G. Crestal bone loss around dental implants placed in head and neck cancer patients treated with different radiotherapy techniques: a prospective cohort study. Int J Oral Maxillofac Surg. 2019 May;48(5):691-696. doi: 10.1016/j.ijom.2018.10.021. Epub 2018 Nov 22. PMID: 30471831. https://doi.org/10.1016/j.ijom.2018.10.021

Curi MM, Condezo AFB, Ribeiro KDCB, Cardoso CL. Long-term success of dental implants in patients with head and neck cancer after radiation therapy. Int J Oral Maxillofac Surg. 2018 Jun;47(6):783-788. doi: 10.1016/j.ijom.2018.01.012. Epub 2018 Feb 6. PMID: 29426738.

Flores-Ruiz R, Castellanos-Cosano L, Serrera-Figallo MA, Cano-Díaz E, Torres-Lagares D, Gutiérrez-Pérez JL. Implant survival in patients with oral cancer: A 5-year follow-up.

J Clin Exp Dent. 2018 Jun 1;10(6):e603-e609. doi: 10.4317/jced.54937. PMID: 29930780; PMCID: PMC6005093.

Menapace DC, Van Abel KM, Jackson RS, Moore EJ. Primary vs Secondary Endosseous Implantation After Fibular Free Tissue Reconstruction of the Mandible for Osteoradionecrosis. JAMA Facial Plast Surg. 2018 Sep 1;20(5):401-408. doi: 10.1001/jamafacial.2018.0263. PMID: 29801119; PMCID: PMC6233614.

Pellegrino G, Tarsitano A, Ferri A, Corinaldesi G, Bianchi A, Marchetti C. Long-term results of osseointegrated implant-based dental rehabilitation in oncology patients reconstructed with a fibula free flap. Clin Implant Dent Relat Res. 2018 Oct;20(5):852-859. doi: 10.1111/cid.12658. Epub 2018 Aug 24. PMID: 30144257.

Sozzi D, Novelli G, Silva R, Connelly ST, Tartaglia GM. Implant rehabilitation in fibula-free flap reconstruction: A retrospective study of cases at 1-18 years following surgery. J Craniomaxillofac Surg. 2017 Oct;45(10):1655-1661. doi: 10.1016/j.jcms.2017.06.021. Epub 2017 Jul 8. PMID: 28823690. https:// doi. org/ 10. 1016/j. jcms. 2017. 06. 021

Burgess M, Leung M, Chellapah A, Clark JR, Batstone MD. Osseointegrated implants into a variety of composite free flaps: A comparative analysis. Head Neck. 2017 Mar;39(3):443-447. doi: 10.1002/hed.24609. Epub 2016 Nov 23. PMID: 27880030.

Woods BJ, Chandu A. Retrospective Study of Survival of Extraoral Endosseous Craniofacial Implants in Head and Neck Surgery Patients. Int J Oral Maxillofac Implants. 2017 Nov/Dec;32(6):1405-1411. doi: 10.11607/jomi.4734. PMID: 29140386.

Wetzels JGH, Meijer GJ, Koole R, Adang EM, Merkx MAW, Speksnijder CM. Costs and clinical outcomes of implant placement during ablative surgery and postponed implant placement in curative oral oncology: a five-year retrospective cohort study. Clin Oral Implants Res. 2017 Nov;28(11):1433-1442. doi: 10.1111/clr.13008. Epub 2017 Mar 2. PMID: 28251678.

Ettl T, Weindler J, Gosau M, Müller S, Hautmann M, Zeman F, Koller M, Papavasileiou D, Bürgers R, Driemel O, Schneider I, Klingelhöffer C, Meier J, Wahlmann U, Reichert TE. Impact of radiotherapy on implant-based prosthetic rehabilitation in patients with head and neck cancer: A prospective observational study on implant survival and quality of life-Preliminary results. J Craniomaxillofac Surg. 2016 Sep;44(9):1453-62. doi: 10.1016/j.jcms.2016.07.016. Epub 2016 Jul 21. PMID: 27519660.

Wu Y, Huang W, Zhang Z, Zhang Z, Zou D. Long-term success of dental implant-supported dentures in postirradiated patients treated for neoplasms of the maxillofacial skeleton: a retrospective study. Clin Oral Investig. 2016 Dec;20(9):2457-2465. doi: 10.1007/s00784-016-1753-z. Epub 2016 Feb 24. Erratum in: Clin Oral Investig. 2017 Jun 21;: PMID: 26907545. https:// doi. org/ 10. 1007/ s00784- 016- 1753-z

Rana MC, Solanki S, Pujari SC, Shaw E, Sharma S, Anand A, Singh HP. Assessment of the Survival of Dental Implants in Irradiated Jaws Following Treatment of Oral Cancer: A Retrospective Study. Niger J Surg. 2016 Jul-Dec;22(2):81-85. doi: 10.4103/1117-6806.182741. PMID: 27843270; PMCID: PMC5013747.

Ernst N, Sachse C, Raguse JD, Stromberger C, Nelson K, Nahles S. Changes in Peri-Implant Bone Level and Effect of Potential Influential Factors on Dental Implants in Irradiated and Nonirradiated Patients Following Multimodal Therapy Due to Head and Neck Cancer: A Retrospective Study. J Oral Maxillofac Surg. 2016 Oct;74(10):1965-73.

doi: 10.1016/j.joms.2016.06.005. Epub 2016 Jun 12. PMID: 27376183. https://doi.org/10.1016/j.joms.2016.06.005

Barber BR, Dziegelewski PT, Chuka R, O'Connell D, Harris JR, Seikaly H. Bone-impacted fibular free flap: Long-term dental implant success and complications compared to traditional fibular free tissue transfer. Head Neck. 2016 Apr;38 Suppl 1:E1783-7. doi: 10.1002/hed.24315. Epub 2015 Dec 17. PMID: 26681661.

Ch'ng S, Skoracki RJ, Selber JC, Yu P, Martin JW, Hofstede TM, Chambers MS, Liu J, Hanasono MM. Osseointegrated implant-based dental rehabilitation in head and neck reconstruction patients. Head Neck. 2016 Apr;38 Suppl 1:E321-7. doi: 10.1002/hed.23993. Epub 2015 Jun 29. PMID: 25546139.

Jackson RS, Price DL, Arce K, Moore EJ. Evaluation of Clinical Outcomes of Osseointegrated Dental Implantation of Fibula Free Flaps for Mandibular Reconstruction. JAMA Facial Plast Surg. 2016 May 1;18(3):201-6. doi: 10.1001/jamafacial.2015.2271. PMID: 26868226.

Kobayashi Y, Sumida T, Ishikawa A, Mori Y. The Contribution of Dental Implants to Functional Artificial Restoration After Treatment of Oral Cancer. Anticancer Res. 2016 Jun;36(6):3053-6. PMID: 27272826.

Wetzels JW, Koole R, Meijer GJ, de Haan AF, Merkx MA, Speksnijder CM. Functional benefits of implants placed during ablative surgery: A 5-year prospective study on the prosthodontic rehabilitation of 56 edentulous oral cancer patients. Head Neck. 2016 Apr;38 Suppl 1:E2103-11. doi: 10.1002/hed.24389. Epub 2016 Feb 13. PMID: 26873437.

Pompa G, Saccucci M, Di Carlo G, Brauner E, Valentini V, Di Carlo S, Gentile T, Guarino G, Polimeni A. Survival of dental implants in patients with oral cancer treated by surgery and radiotherapy: a retrospective study. BMC Oral Health. 2015 Jan 20;15:5. doi: 10.1186/1472-6831-15-5. PMID: 25599761; PMCID: PMC4324417.

Hessling SA, Wehrhan F, Schmitt CM, Weber M, Schlittenbauer T, Scheer M. Implant-based rehabilitation in oncology patients can be performed with high long-term success. J Oral Maxillofac Surg. 2015 May;73(5):889-96. doi: 10.1016/j.joms.2014.11.009. Epub 2014 Dec 6. PMID: 25773649.

Nack C, Raguse JD, Stricker A, Nelson K, Nahles S. Rehabilitation of irradiated patients with chemically modified and conventional SLA implants: five-year follow-up. J Oral Rehabil. 2015 Jan;42(1):57-64. doi: 10.1111/joor.12231. Epub 2014 Sep 18. PMID: 25231029.

Doll C, Nack C, Raguse JD, Stricker A, Duttenhoefer F, Nelson K, Nahles S. Survival analysis of dental implants and implant-retained prostheses in oral cancer patients up to 20 years. Clin Oral Investig. 2015 Jul;19(6):1347-52. doi: 10.1007/s00784-014-1359-2. Epub 2014 Nov 23. PMID: 25416636.

Hakim SG, Kimmerle H, Trenkle T, Sieg P, Jacobsen HC. Masticatory rehabilitation following upper and lower jaw reconstruction using vascularised free fibula flap and enossal implants-19 years of experience with a comprehensive concept. Clin Oral Investig. 2015 Mar;19(2):525-34. doi: 10.1007/s00784-014-1247-9. Epub 2014 May 7. PMID: 24802630.

Karayazgan-Saracoglu B, Atay A, Zulfikar H, Erpardo Y. Assessment of implant stability of patients with and without radiotherapy using resonance frequency analysis. J Oral

Implantol. 2015 Feb;41(1):30-5. doi: 10.1563/AAID-JOI-D-12-00107. Epub 2012 Dec 27. PMID: 23270595.

Jacobsen C, Kruse A, Lübbers HT, Zwahlen R, Studer S, Zemann W, Seifert B, Grätz KW. Is mandibular reconstruction using vascularized fibula flaps and dental implants a reasonable treatment? Clin Implant Dent Relat Res. 2014 Jun;16(3):419-28. doi: 10.1111/cid.12004. Epub 2012 Sep 21. PMID: 22998581.

Korfage A, Raghoebar GM, Slater JJ, Roodenburg JL, Witjes MJ, Vissink A, Reintsema H. Overdentures on primary mandibular implants in patients with oral cancer: a follow-up study over 14 years. Br J Oral Maxillofac Surg. 2014 Nov;52(9):798-805. doi: 10.1016/j.bjoms.2014.05.013. Epub 2014 Jun 17. Erratum in: Br J Oral Maxillofac Surg. 2015 Apr;53(4):e23-5. PMID: 24951245.

Gander T, Studer S, Studer G, Grätz KW, Bredell M. Medium-term outcome of Astra Tech implants in head and neck oncology patients. Int J Oral Maxillofac Surg. 2014 Nov;43(11):1381-5. doi: 10.1016/j.ijom.2014.05.005. Epub 2014 Jun 3. PMID: 24907130.

Dholam KP, Pusalkar HA, Yadav PS, Quazi GA, Somani PP. Implant-retained dental rehabilitation in head and neck cancer patients: an assessment of success and failure. Implant Dent. 2013 Dec;22(6):604-9. doi: 10.1097/ID.0b013e3182a4d7bc. PMID: 24149001.

Buurman DJ, Vaassen LA, Bockmann R, Kessler P. Prosthetic rehabilitation of head and neck cancer patients focusing on mandibular dentures in irradiated patients. Int J Prosthodont. 2013 Nov-Dec;26(6):557-62. doi: 10.11607/ijp.3496. PMID: 24179970.

Fierz J, Hallermann W, Mericske-Stern R. Patients with oral tumors. Part 1: Prosthetic rehabilitation following tumor resection. Schweiz Monatsschr Zahnmed. 2013;123(2):91-105. English, German. PMID: 23512240.

Katsoulis J, Fierz J, IIzuka T, Mericske-Stern R. Prosthetic rehabilitation, implant survival and quality of life 2 to 5 years after resection of oral tumors. Clin Implant Dent Relat Res. 2013 Feb;15(1):64-72. doi: 10.1111/j.1708-8208.2010.00328.x. Epub 2011 Mar 22. PMID: 21435159.

Mizbah K, Dings JP, Kaanders JH, van den Hoogen FJ, Koole R, Meijer GJ, Merkx MA. Interforaminal implant placement in oral cancer patients: during ablative surgery or delayed? A 5-year retrospective study. Int J Oral Maxillofac Surg. 2013 May;42(5):651-5. doi: 10.1016/j.ijom.2012.09.013. Epub 2012 Oct 24. PMID: 23102901.

Buddula A, Assad DA, Salinas TJ, Garces YI, Volz JE, Weaver AL. Survival of dental implants in irradiated head and neck cancer patients: a retrospective analysis. Clin Implant Dent Relat Res. 2012 Oct;14(5):716-22. doi: 10.1111/j.1708-8208.2010.00307.x. Epub 2010 Oct 26. PMID: 20977609.

Mancha de la Plata M, Gías LN, Díez PM, Muñoz-Guerra M, González-García R, Lee GY, Castrejón-Castrejón S, Rodríguez-Campo FJ. Osseointegrated implant rehabilitation of irradiated oral cancer patients. J Oral Maxillofac Surg. 2012 May;70(5):1052-63. doi: 10.1016/j.joms.2011.03.032. Epub 2011 Jul 22. PMID: 21778009.

Linsen SS, Martini M, Stark H. Long-term results of endosteal implants following radical oral cancer surgery with and without adjuvant radiation therapy. Clin Implant Dent Relat

Res. 2012 Apr;14(2):250-8. doi: 10.1111/j.1708-8208.2009.00248.x. Epub 2009 Oct 16. PMID: 19843104.

Fenlon MR, Lyons A, Farrell S, Bavisha K, Banerjee A, Palmer RM. Factors affecting survival and usefulness of implants placed in vascularized free composite grafts used in post-head and neck cancer reconstruction. Clin Implant Dent Relat Res. 2012 Apr;14(2):266-72. doi: 10.1111/j.1708-8208.2009.00250.x. Epub 2009 Oct 16. PMID: 19843102.

Al-Nawas B, Kämmerer PW, Morbach T, Ladwein C, Wegener J, Wagner W. Ten-year retrospective follow-up study of the TiOblast dental implant. Clin Implant Dent Relat Res. 2012 Mar;14(1):127-34. doi: 10.1111/j.1708-8208.2009.00237.x. Epub 2010 Feb 11. PMID: 20156231.

Buddula A, Assad DA, Salinas TJ, Garces YI, Volz JE, Weaver AL. Survival of turned and roughened dental implants in irradiated head and neck cancer patients: a retrospective analysis. J Prosthet Dent. 2011 Nov;106(5):290-6. doi: 10.1016/S0022-3913(11)60133-9. PMID: 22024178.

Buddula A, Assad DA, Salinas TJ, Garces YI. Survival of dental implants in native and grafted bone in irradiated head and neck cancer patients: a retrospective analysis. Indian J Dent Res. 2011 Sep-Oct;22(5):644-8. doi: 10.4103/0970-9290.93449. PMID: 22406706.

Bodard AG, Bémer J, Gourmet R, Lucas R, Coroller J, Salino S, Breton P. Dental implants and free fibula flap: 23 patients. Rev Stomatol Chir Maxillofac. 2011 Apr;112(2):e1-4. doi: 10.1016/j.stomax.2011.01.008. Epub 2011 Mar 2. PMID: 21371725.

Sammartino G, Marenzi G, Cioffi I, Teté S, Mortellaro C. Implant therapy in irradiated patients. J Craniofac Surg. 2011 Mar;22(2):443-5. doi: 10.1097/SCS.0b013e318207b59b. PMID: 21403560.

Heberer S, Kilic S, Hossamo J, Raguse JD, Nelson K. Rehabilitation of irradiated patients with modified and conventional sandblasted acid-etched implants: preliminary results of a split-mouth study. Clin Oral Implants Res. 2011 May;22(5):546-51. doi: 10.1111/j.1600-0501.2010.02050.x. Epub 2010 Dec 2. PMID: 21121960.

Barrowman RA, Wilson PR, Wiesenfeld D. Oral rehabilitation with dental implants after cancer treatment. Aust Dent J. 2011 Jun;56(2):160-5. doi: 10.1111/j.1834-7819.2011.01318.x. PMID: 21623807.

Salinas TJ, Desa VP, Katsnelson A, Miloro M. Clinical evaluation of implants in radiated fibula flaps. J Oral Maxillofac Surg. 2010 Mar;68(3):524-9. doi: 10.1016/j.joms.2009.09.104. PMID: 20171471.

Korfage A, Schoen PJ, Raghoebar GM, Roodenburg JL, Vissink A, Reintsema H. Benefits of dental implants installed during ablative tumour surgery in oral cancer patients: a prospective 5-year clinical trial. Clin Oral Implants Res. 2010 Sep;21(9):971-9. doi: 10.1111/j.1600-0501.2010.01930.x. PMID: 20701621.

Klein MO, Grötz KA, Walter C, Wegener J, Wagner W, Al-Nawas B. Functional rehabilitation of mandibular continuity defects using autologous bone and dental implants - prognostic value of bone origin, radiation therapy and implant dimensions. Eur Surg Res. 2009;43(3):269-75. doi: 10.1159/000229027. Epub 2009 Jul 23. PMID: 19628944.

Cuesta-Gil M, Ochandiano Caicoya S, Riba-García F, Duarte Ruiz B, Navarro Cuéllar C, Navarro Vila C. Oral rehabilitation with osseointegrated implants in oncologic patients. J Oral Maxillofac Surg. 2009 Nov;67(11):2485-96. doi: 10.1016/j.joms.2008.03.001. PMID: 19837322.

Schoen PJ, Raghoebar GM, Bouma J, Reintsema H, Burlage FR, Roodenburg JL, Vissink A. Prosthodontic rehabilitation of oral function in head-neck cancer patients with dental implants placed simultaneously during ablative tumour surgery: an assessment of treatment outcomes and quality of life. Int J Oral Maxillofac Surg. 2008 Jan;37(1):8-16. doi: 10.1016/j.ijom.2007.07.015. Epub 2007 Sep 4. PMID: 17766084.

Alsaadi G, Quirynen M, Komárek A, van Steenberghe D. Impact of local and systemic factors on the incidence of late oral implant loss. Clin Oral Implants Res. 2008 Jul;19(7):670-6. doi: 10.1111/j.1600-0501.2008.01534.x. Epub 2008 May 19. PMID: 18492080.

Nelson K, Heberer S, Glatzer C. Survival analysis and clinical evaluation of implant-retained prostheses in oral cancer resection patients over a mean follow-up period of 10 years. J Prosthet Dent. 2007 Nov;98(5):405-10. doi: 10.1016/S0022-3913(07)60125-5. PMID: 18021829.

Schoen PJ, Raghoebar GM, Bouma J, Reintsema H, Vissink A, Sterk W, Roodenburg JL. Rehabilitation of oral function in head and neck cancer patients after radiotherapy with implant-retained dentures: effects of hyperbaric oxygen therapy. Oral Oncol. 2007 Apr;43(4):379-88. doi: 10.1016/j.oraloncology.2006.04.009. Epub 2006 Sep 25. PMID: 16996783.

Yerit KC, Posch M, Seemann M, Hainich S, Dörtbudak O, Turhani D, Ozyuvaci H, Watzinger F, Ewers R. Implant survival in mandibles of irradiated oral cancer patients. Clin Oral Implants Res. 2006 Jun;17(3):337-44. doi: 10.1111/j.1600-0501.2005.01160.x. PMID: 16672031.

Schepers RH, Slagter AP, Kaanders JH, van den Hoogen FJ, Merkx MA. Effect of postoperative radiotherapy on the functional result of implants placed during ablative surgery for oral cancer. Int J Oral Maxillofac Surg. 2006 Sep;35(9):803-8. doi: 10.1016/j.ijom.2006.03.007. Epub 2006 May 11. PMID: 16697146.

Landes CA, Kovács AF. Comparison of early telescope loading of non-submerged ITI implants in irradiated and non-irradiated oral cancer patients. Clin Oral Implants Res. 2006 Aug;17(4):367-74. doi: 10.1111/j.1600-0501.2005.01227.x. PMID: 16907766.

Bodard AG, Gourmet R, Lucas R, Bonnet E, Breton P. Implants dentaires en territoire irradié: série de 33 patients [Dental implants in irradiated areas: a series of 33 patients]. Rev Stomatol Chir Maxillofac. 2006 Jun;107(3):137-42; discussion 143-4. French. doi: 10.1016/s0035-1768(06)77007-3. PMID: 16804478.

Chiapasco M, Biglioli F, Autelitano L, Romeo E, Brusati R. Clinical outcome of dental implants placed in fibula-free flaps used for the reconstruction of maxillo-mandibular defects following ablation for tumors or osteoradionecrosis. Clin Oral Implants Res. 2006 Apr;17(2):220-8. doi: 10.1111/j.1600-0501.2005.01212.x. PMID: 16584419.

Teoh KH, Huryn JM, Patel S, Halpern J, Tunick S, Wong HB, Zlotolow IM. Implant prosthodontic rehabilitation of fibula free-flap reconstructed mandibles: a Memorial

Sloan-Kettering Cancer Center review of prognostic factors and implant outcomes. Int J Oral Maxillofac Implants. 2005 Sep-Oct;20(5):738-46. PMID: 16274148.

Shaw RJ, Sutton AF, Cawood JI, Howell RA, Lowe D, Brown JS, Rogers SN, Vaughan ED. Oral rehabilitation after treatment for head and neck malignancy. Head Neck. 2005 Jun;27(6):459-70. doi: 10.1002/hed.20176. PMID: 15880417.

Granström G. Osseointegration in irradiated cancer patients: an analysis with respect to implant failures. J Oral Maxillofac Surg. 2005 May;63(5):579-85. doi: 10.1016/j.joms.2005.01.008. PMID: 15883929.

Granström G. Radiotherapy, osseointegration and hyperbaric oxygen therapy. Periodontol 2000. 2003;33:145-62. doi: 10.1046/j.0906-6713.2002.03312.x. PMID: 12950848.

Cao Y, Weischer T. Comparison of maxillary implant-supported prosthesis in irradiated and non-irradiated patients. J Huazhong Univ Sci Technolog Med Sci. 2003;23(2):209-12. doi: 10.1007/BF02859961. PMID: 12973954.

Schoen PJ, Raghoebar GM, Vissink A, Roodenburg JL. Mandibulotomy and implant insertion. Head Neck. 2003 Sep;25(9):748-53. doi: 10.1002/hed.10284. PMID: 12953310.

Visch LL, van Waas MA, Schmitz PI, Levendag PC. A clinical evaluation of implants in irradiated oral cancer patients. J Dent Res. 2002 Dec;81(12):856-9. doi: 10.1177/154405910208101212. PMID: 12454102.

Van Steenberghe D, Jacobs R, Desnyder M, Maffei G, Quirynen M. The relative impact of local and endogenous patient-related factors on implant failure up to the abutment stage. Clin Oral Implants Res 2002;13:617–622.

Goto M, Jin-Nouchi S, Ihara K, Katsuki T. Longitudinal follow-up of osseointegrated implants in patients with resected jaws. Int J Oral Maxillofac Implants. 2002 Mar-Apr;17(2):225-30. PMID: 11958405.

Grötz KA, Wahlmann UW, Krummenauer F, Wegener J, al-Nawas B, Kuffner HD, Wagner W. Prognose und Prognosefaktoren enossaler Implantate im bestrahlten Kiefer [Prognosis and prognostic factors of endosseous implants in the irradiated jaw]. Mund Kiefer Gesichtschir. 1999 May;3 Suppl 1:S117-24. German. doi: 10.1007/PL00014497. PMID: 10414097.

Betz T, Purps S, Pistner H, Bill J, Reuther J. Orale Rehabilitation von Tumorpatienten mit enossalen Implantaten. Implantationserfolg unter besonderer Berücksichtigung der periimplantären Gewebe [Oral rehabilitation of tumor patients with endosseous implants. Implant success with special reference to peri-implant tissue]. Mund Kiefer Gesichtschir. 1999 May;3 Suppl 1:S99-105. German. doi: 10.1007/PL00014529. PMID: 10414094.

Weischer T, Mohr C. Ten-year experience in oral implant rehabilitation of cancer patients: treatment concept and proposed criteria for success. Int J Oral Maxillofac Implants. 1999 Jul-Aug;14(4):521-8. PMID: 10453667.

Schliephake H, Neukam FW, Schmelzeisen R, Wichmann M. Long-term results of endosteal implants used for restoration of oral function after oncologic surgery. Int J Oral Maxillofac Surg. 1999 Aug;28(4):260-5. PMID: 10416892.

Werkmeister R, Szulczewski D, Walteros-Benz P, Joos U. Rehabilitation with dental implants of oral cancer patients. J Craniomaxillofac Surg. 1999 Feb;27(1):38-41. doi: 10.1016/s1010-5182(99)80008-0. PMID: 10188126.

Mericske-Stern R, Perren R, Raveh J. Life table analysis and clinical evaluation of oral implants supporting prostheses after resection of malignant tumors. Int J Oral Maxillofac Implants. 1999 Sep-Oct;14(5):673-80. PMID: 10531739.

Keller EE, Tolman DE, Eckert SE. Maxillary antral-nasal inlay autogenous bone graft reconstruction of compromised maxilla: a 12-year retrospective study. Int J Oral Maxillofac Implants. 1999 Sep-Oct;14(5):707-21. PMID: 10531743.

Granström G, Tjellström A, Brånemark PI. Osseointegrated implants in irradiated bone: a case-controlled study using adjunctive hyperbaric oxygen therapy. J Oral Maxillofac Surg. 1999 May;57(5):493-9. doi: 10.1016/s0278-2391(99)90059-9. PMID: 10319821.

Foster RD, Anthony JP, Sharma A, Pogrel MA. Vascularized bone flaps versus nonvascularized bone grafts for mandibular reconstruction: an outcome analysis of primary bony union and endosseous implant success. Head Neck. 1999 Jan;21(1):66-71. doi: 10.1002/(sici)1097-0347(199901)21:1<66::aid-hed9>3.0.co;2-z. PMID: 9890353.

Esser E, Neukirchen S, Wagner W. Vergleichende Untersuchungen von Brånemark-Implantaten im bestrahlten und nicht bestrahlten Unterkiefer [Comparative studies of Brånemark implants in the irradiated and not irradiated mandible]. Mund Kiefer Gesichtschir. 1999 May;3 Suppl 1:S125-9. German. doi: 10.1007/PL00014498. PMID: 10414098.

Niimi A, Ueda M, Keller EE, Worthington P. Experience with osseointegrated implants placed in irradiated tissues in Japan and the United States. Int J Oral Maxillofac Implants. 1998 May-Jun;13(3):407-11. PMID: 9638012.

Ihara K, Goto M, Miyahara A, Toyota J, Katsuki T. Multicenter experience with maxillary prostheses supported by Brånemark implants: a clinical report. Int J Oral Maxillofac Implants. 1998 Jul-Aug;13(4):531-8. PMID: 9714960.

Brogniez V, Lejuste P, Pecheur A, Reychler H. Dental prosthetic reconstruction of osseointegrated implants placed in irradiated bone. Int J Oral Maxillofac Implants. 1998 Jul-Aug;13(4):506-12. PMID: 9714957.

Andersson G, Andreasson L, Bjelkengren G. Oral implant rehabilitation in irradiated patients without adjunctive hyperbaric oxygen. Int J Oral Maxillofac Implants. 1998 Sep-Oct;13(5):647-54. PMID: 9796148.

August M, Bast B, Jackson M, Perrott D. Use of the fixed mandibular implant in oral cancer patients: a retrospective study. J Oral Maxillofac Surg. 1998 Mar;56(3):297-301. doi: 10.1016/s0278-2391(98)90102-1. PMID: 9496839.

Wagner W, Esser E, Ostkamp K. Osseointegration of dental implants in patients with and without radiotherapy. Acta Oncol. 1998;37(7-8):693-6. doi: 10.1080/028418698430061. PMID: 10050989.

Niimi A, Ueda M, Keller EE, Worthington P. Experience with osseointegrated implants placed in irradiated tissues in Japan and the United States. Int J Oral Maxillofac Implants. 1998 May-Jun;13(3):407-11. PMID: 9638012.

Keller EE, Tolman DE, Zuck SL, Eckert SE. Mandibular endosseous implants and autogenous bone grafting in irradiated tissue: a 10-year retrospective study. Int J Oral Maxillofac Implants. 1997 Nov-Dec;12(6):800-13. PMID: 9425761.

Marker P, Siemssen SJ, Bastholt L. Osseointegrated implants for prosthetic rehabilitation after treatment of cancer of the oral cavity. Acta Oncol. 1997;36(1):37-40. doi: 10.3109/02841869709100729. PMID: 9090963.

Chan MF, Hayter JP, Cawood JI, Howell RA. Oral rehabilitation with implant-retained prostheses following ablative surgery and reconstruction with free flaps. Int J Oral Maxillofac Implants. 1997 Nov-Dec;12(6):820-7. PMID: 9425763.

Ali A, Patton DW, el-Sharkawi AM, Davies J. Implant rehabilitation of irradiated jaws: a preliminary report. Int J Oral Maxillofac Implants. 1997 Jul-Aug;12(4):523-6. PMID: 9274081.

Esser E, Wagner W. Dental implants following radical oral cancer surgery and adjuvant radiotherapy. Int J Oral Maxillofac Implants. 1997 Jul-Aug;12(4):552-7. PMID: 9274085.

Jisander S, Grenthe B, Alberius P. Dental implant survival in the irradiated jaw: a preliminary report. Int J Oral Maxillofac Implants. 1997 Sep-Oct;12(5):643-8. PMID: 9337025.

Roumanas ED, Markowitz BL, Lorant JA, Calcaterra TC, Jones NF, Beumer J 3rd. Reconstructed mandibular defects: fibula free flaps and osseointegrated implants. Plast Reconstr Surg. 1997 Feb;99(2):356-65. doi: 10.1097/00006534-199702000-00008. PMID: 9030140.

McGhee MA, Stern SJ, Callan D, Shewmake K, Smith T. Osseointegrated implants in the head and neck cancer patient. Head Neck. 1997 Dec;19(8):659-65. doi: 10.1002/(sici)1097-0347(199712)19:8<659:aid-hed3>3.0.co;2-4. PMID: 9406744.

Watzinger F, Ewers R, Henninger A, Sudasch G, Babka A, Woelfl G. Endosteal implants in the irradiated lower jaw. J Craniomaxillofac Surg. 1996 Aug;24(4):237-44. doi: 10.1016/s1010-5182(96)80007-2. PMID: 8880450.

Weischer T, Schettler D, Mohr C. Concept of surgical and implant-supported prostheses in the rehabilitation of patients with oral cancer. Int J Oral Maxillofac Implants. 1996 Nov-Dec;11(6):775-81. PMID: 8990640.

Eckert SE, Desjardins RP, Keller EE, Tolman DE. Endosseous implants in an irradiated tissue bed. J Prosthet Dent. 1996 Jul;76(1):45-9. doi: 10.1016/s0022-3913(96)90345-5. PMID: 8814634.

Aldegheri A, Beloni D, Blanc JL, Kaplanski P, Legre R, Zanaret M. La réhabilitation dentaire par fixtures ostéo-intégrables: traitement des cancers oro-maxillo-faciaux. Etude préliminaire de 7 cas [Dental rehabilitation using osseointegrated implants: treatment of oro-maxillo-facial cancer. A preliminary study of 7 cases]. Rev Stomatol Chir Maxillofac. 1996;97(2):108-16. French. PMID: 8685617.

Franzén L, Rosenquist JB, Rosenquist KI, Gustafsson I. Oral implant rehabilitation of patients with oral malignancies treated with radiotherapy and surgery without adjunctive hyperbaric oxygen. Int J Oral Maxillofac Implants. 1995 Mar-Apr;10(2):183-7. PMID: 7744437.

Sclaroff A, Haughey B, Gay WD, Paniello R. Immediate mandibular reconstruction and placement of dental implants. At the time of ablative surgery. Oral Surg Oral Med Oral Pathol. 1994 Dec;78(6):711-7. doi: 10.1016/0030-4220(94)90085-x. PMID: 7898907.

Albrektsson T, Dahl E, Enbom L, Engevall S, Engquist B, Eriksson AR, Feldmann G, Freiberg N, Glantz PO, Kjellman O, et al. Osseointegrated oral implants. A Swedish multicenter study of 8139 consecutively inserted Nobelpharma implants. J Periodontol. 1988 May;59(5):287-96. doi: 10.1902/jop.1988.59.5.287. PMID: 3290429.

S3 – Studies identified in the literature search – included/reason for exclusion

Study	Included	Reason for exclusion
Camolesi et al., 2023	Yes	not applicable
Shahi et al., 2023	Yes	not applicable
Kende et al., 2022	Yes	not applicable
Schiegnitz et al., 2022	Yes	not applicable
Shokouhi and Cerajewska, 2022	Yes	not applicable
Atanásio Pitorro et al., 2022	No	full text was unavailable
Estafanous et al., 2022	No	full text was unavailable
Gupta et al., 2021	Yes	not applicable
In't Veld et al., 2021	Yes	not applicable
Benites Condezo et al., 2021	No	did not investigate the control group - non-irradiated
Toneatti et al., 2021	No	included nonspecific data for head and neck cancer
Lu et al., 2021	No	did not focus on oral rehabilitation with implants in irradiated patients
Panchal et al., 2020	No	included nonspecific data for head and neck cancer
Koudougou et al., 2020	No	were not systematic reviews
Schimmel et al., 2018	No	did not investigate the control group - non-irradiated
Ravi et al., 2017	No	did not investigate the control group - non-irradiated
Smith Nobrega et al., 2016	Yes	not applicable
Chrcanovic et al., 2016	Yes	not applicable
Zhang et al., 2016	No	did not investigate the control group - non-irradiated
Shugaa Addin et al., 2016	No	did not investigate the control group - non-irradiated
Zen Filho et al., 2016	No	did not investigate the control group - non-irradiated
Thanya and Ganapathy, 2016	No	were not systematic reviews
Kotsakis et al., 2015	No	did not focus on oral rehabilitation with implants in irradiated patients
Claudy et al., 2015	No	did not investigate the control group - non-irradiated
Korfage et al., 2015	No	were editorials
Schiegnitz et al., 2015	No	were editorials
Shiegnitz et al., 2014	Yes	not applicable
Recani et al., 2014	No	full text was unavailable
Chambrone et al., 2013	Yes	not applicable
Esposito and Worthington, 2013	No	did not investigate the control group - non-irradiated
Nooh, 2013	No	did not investigate the control group - non-irradiated
Tanaka et al., 2013	No	were not systematic reviews
Chen et al., 2013	No	did not focus on oral rehabilitation with implants in irradiated patients
Dholam and Gurav, 2012	No	were not systematic reviews
Barber et al., 2011	No	did not investigate implant survival
Garg and Guez, 2011	No	full text was unavailable
Javed et al., 2010	No	were not systematic reviews
Hugentobl and Guyot, 2009	No	full text was unavailable
Esposito et al., 2008	No	did not investigate the control group - non-irradiated
Colella et al., 2007	No	full text was unavailable
Schoen et al., 2004	No	were not systematic reviews
Harrison et al., 2003	No	were not systematic reviews
Coulthard et al., 2002	No	did not investigate the control group - non-irradiated

Samouhi and Buchbinder, 2000	No	full text was unavailable
Marx and Morales, 1998	No	were not systematic reviews

S 4 – Justification for downgrading studies evaluated by the AMSTAR 2 tool

Question	Study downgraded	Justification
Q.1	Schiegnitz et al. (2022) e Schiegnitz et al. (2014)	Inclusion of participants with odontogenic tumors and cysts - not included in the PICO acronym
Q.2	Shahi et al. (2023), Schiegnitz et al. (2022), Shokouhi and Cerajewska (2022), Gupta et al. (2021), In't Veld et al. (2021), Smith Nobrega et al. (2016); Chrcanovic et al. (2016) e Shiegnitz et al. (2014), Chambrone et al. (2013)	Did not state that the review methods were established before beginning the study
Q.3	In't Veld et al. (2021)	They did not explain the selection of study designs for inclusion in the review
Q.4	Camolesi et al. (2023), Shahi et al. (2023), Kende et al. (2022), Schiegnitz et al. (2022), Gupta et al. (2021), In't Veld et al. (2021), Smith Nobrega et al. (2016) e Shiegnitz et al. (2014)	They carried out language restrictions without justification
Q.5	Shahi et al. (2023), Schiegnitz et al. (2022), Shokouhi and Cerajewska (2022)	They did not select studies in duplicate
Q.6	Shahi et al. (2023), Schiegnitz et al. (2022), Shokouhi and Cerajewska (2022), In't Veld et al. (2021), Chrcanovic et al. (2016)	They did not perform data extraction in duplicate
Q.7	Camolesi et al. (2023), Shahi et al. (2023), Kende et al. (2022), Schiegnitz et al. (2022), Shokouhi and Cerajewska (2022), Gupta et al. (2021), In't Veld et al. (2021), Smith Nobrega et al. (2016), Chrcanovic et al. (2016) e Shiegnitz et al. (2014)	They did not provide a list of excluded studies, justified
Q.8	Shahi et al. (2023), Kende et al. (2022)	Did not describe the included studies in detail
Q.9	In't Veld et al. (2021), Smith Nobrega et al. (2016); Chrcanovic et al. (2016) e Shiegnitz et al. (2014)	They did not use an adequate technique to assess the risk of bias in primary studies
Q.10	Camolesi et al. (2023), Shahi et al. (2023), Kende et al. (2022), Schiegnitz et al. (2022), Shokouhi and Cerajewska (2022), Gupta et al. (2021), In't Veld et al. (2021), Smith Nobrega et al. (2016); Chrcanovic et al. (2016) e Shiegnitz et al. (2014), Chambrone et al. (2013)	They did not inform the source of funding in primary studies
Q.11	Kende et al. (2022)	Meta-analysis results are not consistent
Q.12	Shahi et al. (2023), Kende et al. (2022); Shiegnitz et al. (2014)	They did not assess the potential impact of the risk of bias in primary studies on the results of the meta-analysis
Q.13	Camolesi et al. (2023), Shahi et al. (2023), Kende et al. (2022); Gupta et al. (2021)	They did not take into account the risk of bias in primary studies when discussing/interpreting the review results
Q.14	Kende et al. (2022)	They did not provide an explanation for the heterogeneity observed in the review results
Q.15	Shokouhi and Cerajewska (2022), Gupta et al. (2021), In't Veld et al. (2021), Chambrone et al. (2013)	Did not investigate publication bias

Q.16	Camolesi et al. (2023), Smith Nobrega et al. (2016); Chrcanovic et al. (2016), Shiegnitz et al. (2014)	Did not report any sources of conflicts of interest

Q.1. Did the research questions and inclusion criteria for the review include the components of PICO? Q.2. Did the report of the review contain an explicit statement that the review methods were established prior to the conduct of the review and did the report justify any significant deviations from the protocol? Q. 3. Did the review authors explain their selection of the study designs for inclusion in the review? Q. 4. Did the review authors use a comprehensive literature search strategy? Q. 5. Did the review authors perform study selection in duplicate? Q. 6. Did the review authors perform data extraction in duplicate? Q. 7. Did the review authors provide a list of excluded studies and justify the exclusions? Q. 8. Did the review authors describe the included studies in adequate detail? Q. 9. Did the review authors use a satisfactory technique for assessing the risk of bias (RoB) in individual studies that were included in the review? Q. 10. Did the review authors report on the sources of funding for the studies included in the review? Q. 11. If meta-analysis was performed, did the review authors use appropriate methods for statistical combination of results? Q. 12. If meta-analysis was performed, did the review authors assess the potential impact of RoB in individual studies on the results of the meta-analysis or Other evidence synthesis? Q. 13. Did the review authors account for RoB in primary studies when interpreting/discussing the results of the review? Q. 14. Did the review authors provide a satisfactory explanation for, and discussion of, any heterogeneity observed in the results of the review? Q. 15. If they performed quantitative synthesis did the review authors carry out an adequate investigation of publication bias (small study bias) and discuss its likely impact on the results of the review? Q.16. Did the review authors report any potential sources of conflict of interest, including any funding they received for conducting the review?
