# Supplementary material for: Survival of dental implants in irradiated head and neck cancer patients compared to non-irradiated patients: An umbrella review
Source: PLoS One. 2025 Sep 3;20(9):e0324388. doi: 10.1371/journal.pone.0324388 (PMC12407548; doi:10.1371/journal.pone.0324388)
Supplement: Attachment — Submitted filename: PONE-D-24-47771_reviewer.pdf [file pone.0324388.s002.pdf]

# PLOS ONE

# Survival of dental implants in irradiated head and neck cancer patients compared to non-irradiated patients: An umbrella review

--Manuscript Draft--

| | |
|---|---|
| **Manuscript Number:** | PONE-D-24-47771 |
| **Article Type:** | Research Article |
| **Full Title:** | Survival of dental implants in irradiated head and neck cancer patients compared to non-irradiated patients: An umbrella review |
| **Short Title:** | Survival of dental implants in irradiated head and neck cancer patients compared to non-irradiated patients |
| **Corresponding Author:** | Caio Fernando Teixeira Portela, Ph.D<br>Universidade Federal de Minas Gerais Escola de Engenharia<br>Belo Horizonte, BRAZIL |
| **Keywords:** | Keywords: Dental implants, radiotherapy, head and neck cancer, systematic review, umbrella review. |
| **Abstract:** | Background: There is an increasing demand for oral rehabilitation in patients undergoing irradiation for head and neck cancer treatment. Although radiotherapy appears to adversely affect implanted oral rehabilitation, the evidence provided by previous systematic reviews in the field remains controversial. Thus, this umbrella review aimed to evaluate the survival of dental implants installed before and/or after radiotherapy in patients with head and neck cancer compared to those not irradiated. Methods: A comprehensive search of electronic databases was performed in the Medline/PubMed, Cochrane Library, Embase, Web of Science, and Google Scholar databases, including manual searches, from inception to February, 2024, with no language or date restrictions. The Preferred Reporting Items for Systematic Reviews and Meta-Analyses (PRISMA) 2020 guidelines were followed. Survival percentage of dental implants was the primary outcome. A MeaSurement Tool to Assess systematic Reviews (AMSTAR) 2 was used as a critical appraisal tool for the included studies. The protocol for this review has been registered with PROSPERO (CRD42023406059). Results: Of the 1,811 articles screened, 11 articles that evaluated 73,674 implants were included. Quantitative analyses showed 2,674 failures out of 14,471 implants installed in irradiated bone and 1,825 failures out of 34,092 implants in non-irradiated bone. The survival rate was 81.52% for irradiated implants and 94.64% for non-irradiated implants. A significant difference in survival in favor of implants in non-irradiated bone is supported by 11 meta-analyses. The included systematic reviews showed critically low methodological quality. Conclusions: Despite the critically low methodological quality of the included studies, implants installed in irradiated patients had a higher percentage of failures. Further clinical studies employing improved research designs are warranted. |
| **Order of Authors:** | Caio Fernando Teixeira Portela, Ph.D |
| | Maria Cândida Dourado Pacheco, Doctorate |
| | Alexandre Henrique dos Reis Prado, Doctorate |
| | Tarcísio Passos Ribeiro Campos, Doctorate |
| | Lara Cancella de Arantes, Master |
| | Arno Heeren de Oliveira, Doctorate |
| | Danilo Viegas da Costa, Master |
| **Additional Information:** | |
| **Question** | **Response** |
| **Financial Disclosure**<br><br>Enter a financial disclosure statement that | The author(s) received no specific funding for this work. |

*Powered by Editorial Manager® and ProduXion Manager® from Aries Systems Corporation*

| | |
|---|---|
| describes the sources of funding for the work included in this submission. Review the submission guidelines for detailed requirements. View published research articles from *PLOS ONE* for specific examples.<br><br>This statement is required for submission and **will appear in the published article** if the submission is accepted. Please make sure it is accurate.<br><br>**Funded studies**<br>Enter a statement with the following details:<br>• Initials of the authors who received each award<br>• Grant numbers awarded to each author<br>• The full name of each funder<br>• URL of each funder website<br>• Did the sponsors or funders play any role in the study design, data collection and analysis, decision to publish, or preparation of the manuscript?<br><br>Did you receive funding for this work? | |
| **Competing Interests**<br><br>Use the instructions below to enter a competing interest statement for this submission. On behalf of all authors, disclose any competing interests that could be perceived to bias this work—acknowledging all financial support and any other relevant financial or non-financial competing interests.<br><br>This statement is required for submission and **will appear in the published article** if the submission is accepted. Please make sure it is accurate and that any funding sources listed in your Funding Information later in the submission form are also declared in your Financial Disclosure statement.<br><br>View published research articles from *PLOS ONE* for specific examples. | The authors have declared that no competing interests exist |

| | |
|---|---|
| **NO authors have competing interests**<br><br>Enter: *The authors have declared that no competing interests exist.*<br><br>**Authors with competing interests**<br><br>Enter competing interest details beginning with this statement:<br><br>*I have read the journal's policy and the authors of this manuscript have the following competing interests: [insert competing interests here]*<br><br>* typeset | |
| **Ethics Statement**<br><br>Enter an ethics statement for this submission. This statement is required if the study involved:<br><br>• Human participants<br>• Human specimens or tissue<br>• Vertebrate animals or cephalopods<br>• Vertebrate embryos or tissues<br>• Field research<br><br>Write "N/A" if the submission does not require an ethics statement.<br><br>General guidance is provided below. Consult the submission guidelines for detailed instructions. **Make sure that all information entered here is included in the Methods section of the manuscript.** | N/A |

| | |
|---|---|
| **Format for specific study types**<br><br>**Human Subject Research (involving human participants and/or tissue)**<br>• Give the name of the institutional review board or ethics committee that approved the study<br>• Include the approval number and/or a statement indicating approval of this research<br>• Indicate the form of consent obtained (written/oral) or the reason that consent was not obtained (e.g. the data were analyzed anonymously)<br><br>**Animal Research (involving vertebrate animals, embryos or tissues)**<br>• Provide the name of the Institutional Animal Care and Use Committee (IACUC) or other relevant ethics board that reviewed the study protocol, and indicate whether they approved this research or granted a formal waiver of ethical approval<br>• Include an approval number if one was obtained<br>• If the study involved *non-human primates*, add *additional details* about animal welfare and steps taken to ameliorate suffering<br>• If anesthesia, euthanasia, or any kind of animal sacrifice is part of the study, include briefly which substances and/or methods were applied<br><br>**Field Research**<br><br>Include the following details if this study involves the collection of plant, animal, or other materials from a natural setting:<br>• Field permit number<br>• Name of the institution or relevant body that granted permission | |
| **Data Availability**<br><br>Authors are required to make all data underlying the findings described fully available, without restriction, and from the time of publication. PLOS allows rare exceptions to address legal and ethical concerns. See the PLOS Data Policy and FAQ for detailed information. | Yes - all data are fully available without restriction |

| | |
|---|---|
| A Data Availability Statement describing where the data can be found is required at submission. Your answers to this question constitute the Data Availability Statement and **will be published in the article**, if accepted.<br><br>**Important:** Stating 'data available on request from the author' is not sufficient. If your data are only available upon request, select 'No' for the first question and explain your exceptional situation in the text box.<br><br>Do the authors confirm that all data underlying the findings described in their manuscript are fully available without restriction? | |
| **Describe where the data may be found in full sentences. If you are copying our sample text, replace any instances of XXX with the appropriate details.**<br><br>• If the data are **held or will be held in a public repository**, include URLs, accession numbers or DOIs. If this information will only be available after acceptance, indicate this by ticking the box below. For example: *All XXX files are available from the XXX database (accession number(s) XXX, XXX.*).<br>• If the data are all contained **within the manuscript and/or Supporting Information files**, enter the following: *All relevant data are within the manuscript and its Supporting Information files.*<br>• If neither of these applies but you are able to provide **details of access elsewhere**, with or without limitations, please do so. For example:<br><br>*Data cannot be shared publicly because of [XXX]. Data are available from the XXX Institutional Data Access / Ethics Committee (contact via XXX) for researchers who meet the criteria for access to confidential data.*<br><br>*The data underlying the results presented in the study are available from (include the name of the third party* | All relevant data are within the manuscript and its Supporting Information files. |

| | |
|---|---|
| *and contact information or URL).*<br>• This text is appropriate if the data are owned by a third party and authors do not have permission to share the data.<br><br>* typeset | |
| Additional data availability information: | Tick here if the URLs/accession numbers/DOIs will be available only after acceptance of the manuscript for publication so that we can ensure their inclusion before publication.; Tick here if your circumstances are not covered by the questions above and you need the journal's help to make your data available. |

Powered by Editorial Manager® and ProduXion Manager® from Aries Systems Corporation

Manuscript Click here to access/download;Manuscript;artigo plosone.pdf

# Survival of dental implants in irradiated head and neck cancer patients compared to non-irradiated patients: An umbrella review

Maria Cândida Dourado Pacheco[1], Alexandre Henrique dos Reis-Prado[2], Danilo Viegas da Costa, Lara Cancella de Arantes[3], Caio Fernando Teixeira Portela[1], Tarcísio Passos Ribeiro Campos[1], Arno Heeren de Oliveira[1]

[1]Department of Nuclear Engineering, Federal University of Minas Gerais, School of Engineering, Belo Horizonte, Minas Gerais, Brazil

[2]Department of Cariology, Restorative Dentistry and Endodontics, University of Michigan, School of Dentistry, Ann Abor, Michigan, USA

[3]Restorative Dentistry, Federal University of Minas Gerais, School of Dentistry, Belo Horizonte, Minas Gerais, Brazil

**Correspondence**

Caio Fernando Teixeira Portela

## ABSTRACT

**Background:** There is an increasing demand for oral rehabilitation in patients undergoing irradiation for head and neck cancer treatment. Although radiotherapy appears to adversely affect implanted oral rehabilitation, the evidence provided by previous systematic reviews in the field remains controversial. Thus, this umbrella review aimed to evaluate the survival of dental implants installed before and/or after radiotherapy in patients with head and neck cancer compared to those not irradiated. **Methods:** A comprehensive search of electronic databases was performed in the Medline/PubMed, Cochrane Library, Embase, Web of Science, and Google Scholar databases, including manual searches, from inception to February, 2024, with no language or date restrictions. The Preferred Reporting Items for Systematic Reviews and Meta-Analyses (PRISMA) 2020 guidelines were followed. Survival percentage of dental implants was the primary outcome. A MeaSurement Tool to Assess systematic Reviews (AMSTAR) 2 was used as a critical appraisal tool for the included studies. The protocol for this review has been registered with PROSPERO (CRD42023406059). **Results:** Of the 1,811 articles screened, 11 articles that evaluated 73,674 implants were included. Quantitative analyses showed 2,674 failures out of 14,471 implants installed in irradiated bone and 1,825 failures out of 34,092 implants in non-irradiated bone. The survival rate was 81.52% for irradiated implants and 94.64% for non-irradiated implants. A significant difference in survival in favor of implants in non-irradiated bone is supported by 11 meta-analyses. The included systematic reviews showed critically low methodological quality. **Conclusions:** Despite the critically low methodological quality of the included studies, implants installed in irradiated patients had a higher percentage of failures. Further clinical studies employing improved research designs are warranted.

**Keywords:** Dental implants, radiotherapy, head and neck cancer, systematic review, umbrella review.

## 1 | INTRODUCTION

Osseointegrated dental implants are widely used and can provide a safe treatment option for edentulous patients, demonstrating promising survival rates within a 5 to 10-year follow-up period (Jung et al., 2012) [1]. However, the risks associated with implant failures have been extensively investigated over the years (Papi et al., 2019; Albrega et al., 2020; Pieralli et al., 2021; Li et al., 2022) [2, 3, 4, 5]. Possible reasons for failure are low insertion torque of implants planned to be loaded immediately or early, inexperienced surgeons inserting the implants, insertion of the implant into the maxilla, the patient history (radiotherapy, bruxism, periodontitis and early implant loss) (Chrcanovic et al., 2014; Do et al., 2020) [6, 7].

There is an increasing demand for oral rehabilitation in patients undergoing irradiation for head and neck cancer treatment (Chrcanovic et al., 2016) [8]. Cases of head and neck cancer are significantly increasing among the population (Sung et al., 2021) [9], which is also associated with the increased frequency of dental implants installed in these patients (Shiegnitz et al., 2021) [10]. However, radiotherapy seems to adversely affect implanted oral rehabilitation (Ernst et al., 2016; Neckel et al., 2021; Wolf et al., 2021) [11, 12, 13] due to radiation-induced fibroatrophic process (Delanian and Lefaix, 2004) [14], leading to tissue hypoxia, hypocellularity, hypovascularity and reduced bone regeneration capacity (Marx; Johnson, 1987) [15].

Although several systematic reviews have investigated the survival of dental implants in head and neck cancer patients undergoing radiotherapy involving various clinical analyses, the presented results have been inconclusive (In’t Veld et al., 2021; Shiegnitz et al., 2022; Kende et al., 2022). [16, 17, 18] Recently evaluated in two umbrella reviews (Marques et al., 2023; Zarzar et al., 2024) [19, 20], oral rehabilitation in patients with irradiated head and neck cancer is a valid therapy. However, due to heterogeneity and the lack of a comparator group in most of the included systematic reviews, the recommendation is that the results presented be interpreted with caution. The present umbrella review aimed to analyse the available relevant systematic reviews, comparing irradiated versus non-irradiated patients, in an attempt to determine whether there is an influence of head and neck radiotherapy on dental implant survival in patients with head and neck cancer.

## 2 | MATERIALS AND METHODS

This study followed the guidelines of the Preferred Reporting Items for Systematic Reviews and Meta-Analyses (PRISMA) 2020 (Page et al., 2021) [21]. The research protocol was registered on the International Prospective Register of Systematic Reviews (PROSPERO) database under the number CRD42023406059.

### 2.1 PICO formulation

The PICO (Population; Intervention; Comparison; Outcome) strategy was used to formulate the following research question: "What is the survival of dental implants installed before and/or after radiotherapy in patients with head and neck cancer, compared to those not exposed to radiation?" (Table 1).

### 2.2 | Eligibility Criteria

The inclusion criterion were systematic reviews, with or without meta-analysis, that assessed the survival of dental implants installed before and/or after radiotherapy in patients with head and neck cancer, compared to patients with head and neck cancer in whom implants were installed in non-irradiated bone. However, systematic reviews of animal studies, other types of reviews different from systematic reviews, and studies for which the full text was unavailable were excluded.

### 2.3 | Research Sources and Search Strategy

The electronic search was conducted by two independent reviewers (MCDP and LCA), including studies published in PubMed/MEDLINE, Cochrane Library, Embase, and Web of Science, from their inception until February 23, 2024. Additionally, grey literature search was searched in Google Scholar database. Search strategies were developed for each database using indexed terms and synonyms (Supporting Information S1). A manual search of references was performed in all included studies. In case of doubt, a third reviewer (DVC) was consulted. There were no restrictions on language or year of publication for the articles.

### 2.4 | Study Selection Process

The selection of studies was conducted by two reviewers (MCDP and LCA) independently, in a two-stage process using the Ryyan app (Qatar Foundation, Qatar) (Ouzzani et al., 2016) [22]. Duplicate studies were excluded using reference management software (EndNote® X9; Clarivate, Philadelphia, PA, USA). In the first stage, titles and abstracts were assessed, and in the next stage, the full text selected from the titles/abstracts

was read, and those that did not meet the eligibility criteria were excluded. Disagreements were resolved by a third reviewer (DVC). Contacting authors to clarify doubts was not necessary. The level of agreement between the reviewers was assessed by Cohen's Kappa (Landis and Koch, 1977) [23].

### 2.5 | Data Extraction and Analysis

Data were collected by two reviewers (MCDP and LCA) independently, based on the main characteristics of the studies, in an Excel form created and customized for the current review and included: study and publication year, number of participants/age range, country of publication, number of included studies/study design, presence of meta-analysis, variation in radiation dose (Gy)/implant installation time, number of failed/installed implants, implantation site, time interval between radiotherapy and implant installation, follow-up period, implant characteristics, cancer characteristics, adjuvant therapy, bone crest stability, postoperative complications, prosthetic rehabilitation. Subsequently, a third author (DVC) reviewed the data. In cases of missing data, authors would be contacted by email twice.

### 2.6 | Assessment of Methodological Quality

The quality of included systematic reviews was appraised by using the A MeaSurement Tool to Assess Systematic Reviews (AMSTAR 2) tool (Shea et al., 2017) [24], and it was independently assessed by two reviewers (MCDP and LCA). Disagreements were resolved by a third reviewer (DVC). The methodological quality of systematic reviews was assessed using the, which consists of 16 items, where items 2, 4, 7, 9, 11, 13, and 15 are considered critical. Questions are answered with options: yes, partial yes, no, or meta-analysis not conducted. The overall confidence rating of the review's results is categorized as high (none or one non-critical weakness), moderate (more than one non-critical weakness), low (one critical flaw with or without non-critical weaknesses), or critically low (more than one critical flaw with or without non-critical weaknesses).

### 2.7 | Results Synthesis

A descriptive synthesis of the results of the included studies was conducted. If a study did not provide raw data on implant failure but presented percentages related to implant survival, the data were converted when necessary. The percentage of implant survival was calculated by arithmetic mean.

## 3 | RESULTS

### 3.1 | Study Selection

The PRISMA flowchart depicting the study selection process is presented in Figure 1. The literature search strategy identified 1,811 articles. After removing 670 duplicates, the remaining 1,141 publications were evaluated by reading titles/abstracts. A total of five additional reports were identified through manual search. This resulted in 45 records selected for reading the full text. Thirty-four references were excluded because 11 did not investigate the control group - non-irradiated (Benites Condezo et al., 2021; Schimmel et al., 2018; Ravi et al., 2017; Zhang et al., 2016; Shugaa Addin et al., 2016; Zen Filho et al., 2016; Claudy et al., 2015; Esposito and Worthington, 2013; Nooh, 2013; Esposito et al., 2008; Coulthard et al., 2002) [25, 26, 27, 28, 29, 30, 31, 32, 33, 34, 35], eight were not systematic reviews (Koudougou et al., 2020; Thanya and Ganapathy, 2016; Tanaka et al., 2013; Dholam and Gurav, 2012; Javed et al., 2010; Schoen et al., 2004; Harrison et al., 2003; Marx and Morales, 1998) [36, 37, 38, 39, 40, 41, 42, 43], in 7 references the full text was unavailable (Atanásio Pitorro et al., 2022; Estafanous et al., 2022; Recani et al., 2014; Garg and Guez, 2011; Hugentobl and Guyot, 2009; Colella et al., 2007; Samouhi and Buchbinder, 2000) [44, 45, 46, 47, 48, 49, 50], three did not focus on oral rehabilitation with implants in irradiated patients (Lu et al., 2021; Kotsakis et al., 2015; Chen et al., 2013) [51, 52, 53], two included nonspecific data for head and neck cancer (Toneatti et al., 2021; Panchal et al., 2020) [54, 55]; two were editorials (Korfage et al., 2015; Schiegnitz et al., 2015) [56, 57], and one did not investigate implant survival (Barber et al., 2011) [58]. Finally, eleven systematic reviews were included for qualitative synthesis. The inter-rater kappa coefficients indicated an almost perfect agreement level among reviewers during study selection, with values greater than 0.800 for the databases.

### 3.2 | Main characteristics of included reviews

Tables 2 and 3 show the characteristics of the eligible systematic reviews. A total of 16,193 patients who received 73,674 dental implants were included. Of these, approximately 31,137 were installed in irradiated sites. All selected studies (Camolesi et al., 2023; Shahi et al., 2023; Kende et al., 2022; Schiegnitz et al., 2022; Shokouhi and Cerajewska, 2022; Gupta et al., 2021; In't Veld et al., 2021; Smith Nobrega et al., 2016; Chrcanovic et al., 2016; Chambrone et al., 2013) [59, 60, 18, 17, 61, 62, 16, 63, 8, 64] assessed the survival of dental implants installed after the diagnosis of head and neck

cancer. One systematic review (Shiegnitz et al., 2022) [17] was an update of a previous study elaborated by the same research team (Shiegnitz et al., 2014) [65]. The age range of participants varied from 6 to 94 years, with one study not reporting the sample age (Chambrone et al., 2013) [64]. Among the included reviews, most evaluated data from retrospective studies. All included studies conducted meta-analysis.

The minimum radiation dose among the studies ranged from 10 Gy to 40 Gy and the maximum dose from 72 Gy to 145 Gy; two studies did not mention the radiation dose (Shahi et al., 2023; Chambrone et al., 2013) [60, 64]. Regarding the timing of implant installation, five articles described implants installed before and after radiotherapy (Kende et al., 2022; Schiegnitz et al., 2022; Smith Nobrega et al., 2016; Chrcanovic et al., 2016; Chambrone et al., 2013) [18, 17, 63, 8, 64], four reported implants installed after radiotherapy (Camolesi et al., 2023; Shahi et al., 2023; Shokouhi and Cerajewska, 2022; Gupta et al., 2021) [59, 60, 61, 62]; one study evaluated implant installation during panendoscopy or ablative surgery - before radiotherapy (In't Veld et al., 2021) [16]. All selected studies evaluated the survival of implants installed in the maxilla and mandible. While seven studies investigated implants installed in native and grafted bone (Camolesi et al., 2023; Kende et al., 2022; Schiegnitz et al., 2022; Gupta et al., 2021; In't Veld et al., 2021; Smith Nobrega et al., 2016; Chrcanovic et al., 2016) [59, 18, 17, 62, 16, 63, 8], two analyzed implants installed only in native bone (Shokouhi and Cerajewska, 2022; Chambrone et al., 2013) [61, 64], and one study did not mention (Shahi et al., 2023) [60]. Only one study investigated the time interval between radiotherapy and implantation (Chrcanovic et al., 2016) [8]. The follow-up time among the studies varied from one month to 23 years, with one study not mentioning the follow-up period (Shahi et al., 2023) [60]. More details regarding the study design, number of implants installed, number of failed implants, and implant survival percentage extracted from the primary articles are described in Supporting Information S2.

### 3.3 Synthesis of results

*Survival percentage of dental implants in irradiated and non-irradiated patients*

The survival rate of implants was higher among non-irradiated patients according to data from 11 meta-analyses. Conversely, three meta-analyses showed no significant difference between the irradiated and non-irradiated groups (Kende et al., 2022; In't Veld et al., 2021; Shiegnitz et al., 2014 – literature between 2007-2013). Data were reported in four

time periods (1990-2021, 1990-2006, 2007-2013, and 2013-2021) [18, 16, 65] and two follow-up periods (≥ 3 years of follow-up and ≥ 5 years of follow-up). The effect estimate and precision of each meta-analysis are presented in Table 4. Further information on the number of failed/installed implants, number of primary articles included in the meta-analysis, and the percentage of survival is also shown in Table 4.

Overall, 48,563 implants were evaluated, including 14,471 with 2,674 failures in the irradiated group and 34,092 with 1,825 failures in the non-irradiated group. The survival percentage was 81.52% and 94.64%, respectively.

*Survival percentage of Implants Installed in Irradiated Maxilla versus Irradiated Mandible*

Two meta-analyses (Chrcanovic et al., 2016; Chambrone et al., 2013) [8, 64] showed higher implant survival installed in irradiated mandible compared to irradiated maxilla (RR: 3.16; 95% CI: 1.76-5.68; p=0.0001; I2=70%) (RR: 5.96; 95% CI: 2.71-13.12; p<0.00001; I2=33%). A total of 3,378 implants were evaluated, including 897 with 188 failures in the irradiated maxilla group and 2,481 with 160 failures in the irradiated mandible group. The survival percentage was 79.04% and 93.55%, respectively.

In the study by Shokouhi and Cerajewska (2022) [61], implants installed in the maxilla were significantly more likely to fail than those installed in the mandible (OR: 5.03; 95% CI: 1.07-23.58; p=0.04; I2=87%).

*Survival of Implants Installed in Irradiated Grafted Bone versus Irradiated Native Bone*

Two meta-analyses (Shiegnitz et al., 2022 – literature 1990-2021) showed higher implant survival in irradiated native bone [17]. (OR: 2.06; 95% CI: 1.44-2.94; p<0.0001; I2=0% - follow-up ≥36 months) (OR: 2.26; 95% CI: 1.50-3.40; p<0.0001; I2=12% - follow-up ≥60 months). Conversely, one meta-analysis (Chrcanovic et al., 2016) [8] showed no significant difference between irradiated grafted bone and irradiated native bone (RR: 1.35; 95% CI: 0.93-1.94; p=0.11; I2=48%). A total of 1,740 implants were evaluated, including 596 with 106 failures in the irradiated grafted bone group and 1,144 with 148 failures in the irradiated native bone group. The survival percentage was 82.21% and 87.06%, respectively.

Regarding the time interval between the end of radiotherapy and implant installation (up to 12 months versus after 12 months), and the evaluation of radiation dose (high doses

versus low doses), there was no influence of these variables on implant failure among the evaluated groups (Chrcanovic et al., 2016) [8].

### 3.4 | Methodological Quality of Systematic Reviews

Overall, according to the evaluated domains using the AMSTAR 2 tool, the eligible reviews exhibited critically low quality (Table 5). The justifications for downgrading the studies are detailed in the Supporting Information S3.

## DISCUSSION

The present umbrella review included eleven systematic reviews published between 2013 and 2023, assessing the survival of 14,471 dental implants installed before and/or after radiotherapy in head and neck cancer patients, compared to 34,092 implants in non-irradiated patients. Overall, the survival rate of implants installed in irradiated patients was considered inferior compared to those installed in non-irradiated patients. Implant survival in the irradiated maxilla was significantly lower compared to the irradiated mandible. Dental implants installed in irradiated grafted bone were considered more prone to failure compared to irradiated native bone. The methodological quality of the selected studies was considered critically low.

Evidence from systematic reviews supports that implant survival is influenced by the effect of radiotherapy (Panchal et al., 2020; Toneatti et al., 2021) [55, 54]. Zarzar et al., (2024) [20], in an umbrella review, demonstrated that the implant survival rate in irradiated patients was lower compared to non-irradiated patients, with follow-up ranging from 1 month to 23 years. The present umbrella review evaluated and summarized scientific evidence available in the literature, corroborating previous reviews. It is important to mention that the analyzed data had different follow-up periods, which could cause significant errors in the descriptive synthesis presented in this study.

The impact of radiation dose on the survival of dental implants installed in head and neck cancer patients depends on the specific dose at the implant site (>50 Gy) and the parotid gland (>30 Gy) (Wolf et al., 2021) [13]. An umbrella review reported that radiation doses lower than 50 Gy were associated with better survival rates (Marques et al., 2023) [19]. This is consistent with the results observed in this umbrella review. It is important to consider that these data compare high doses with low doses, not detailing the implantation site with the target volume of radiation. Moreover, the systematic reviews included

studies that varied the maximum doses from 72 Gy to 145 Gy, which may represent a significant risk of implant failure and osteoradionecrosis (Anderson et al., 2013) [66].

Most implants evaluated in this review were installed after the completion of radiotherapy. Implants installed after the end of radiotherapy were significantly more likely to fail than those installed in non-irradiated bone (Gupta et al., 2021; Shokouhi and Cerajewka, 2021) [62, 61]. Whereas implants installed immediately during ablative surgery (pre-RT) showed no statistically significant difference between irradiated and non-irradiated groups (In't Veld et al., 2021) [16]. There is a trend to install the implant during tumor removal, significantly reducing prosthetic rehabilitation time and, on the other hand, increasing complications that may hinder implantation success, such as obtaining a well-positioned implant (Di Carlo et al., 2019; Patel et al., 2020; Albrega et al., 2020) [67, 68, 3].

The time interval between radiotherapy and implant surgery may influence implant survival. A meta-analysis showed no statistically significant difference when comparing implant failures installed before and after 12 months of radiotherapy (Chrcanovic et al., 2016) [8]. Claudy et al. (2013) [31], in a meta-analysis, evaluated the time interval between 6 and 12 months after radiotherapy versus 12 months after radiotherapy, and an increased risk of 34% was observed for implants installed between 6 and 12 months post-radiotherapy. The literature recommends implant installation at least six months after the completion of radiotherapy, the expected period for the end of the acute process in bone and soft tissues induced by radiation (Marx and Johnson, 1987) [15].

The umbrella reviews, Zarzar et al. (2024) [20] and Marques et al. (2023) [19], using the AMSTAR 2 tool, included five systematic reviews in common with this study (Chambrone et al., 2013; Shiegnitz et al., 2014; Chrcanovic et al., 2016; Smith Nobrega et al., 2016; In't Veld et al., 2021) [64, 65, 8, 63, 16]. Zarzar et al. (2024) [20] classified the systematic reviews of Shiegnitz et al. (2014) [65], Smith Nobrega et al. (2016) [63], and In't Veld et al. (2021) [16] as critically low, which is in line with the results observed in this study. In contrast, the systematic review by Chambrone et al. (2013) [64] was considered high quality and Chrcanovic et al. (2016) [8] as low quality. This difference could be explained by the complexity associated with judging the issues. Marques et al. (2023) [19] analyses combined responses to the 16 individual items and performed an overall score to assess the risk of bias of the studies, making the classification criteria different from the assessments of this study.

The present study investigated irradiated head and neck cancer patients, recommending caution in interpreting the results, despite the high survival rate demonstrated for dental implants. The evaluation of methodological quality, through the AMSTAR 2 tool, showed that although the included studies reported the use of the PRISMA guideline, only two systematic reviews reported prior registration of the research protocol (Camolesi et al., 2023; Kende et al., 2022) [59, 18]. The PRISMA guideline itself advises indicating where the review protocol can be accessed to allow readers to assess whether any deviations from the protocol compared to what was done may have introduced bias.

Some limitations should be considered when interpreting the results of this umbrella review. The search strategy identified only eleven systematic reviews, all classified with critically low methodological quality. Considering the retrospective nature of most primary studies included in the selected systematic reviews, it was observed that the reported data were notably heterogeneous, especially for questions related to implant characteristics, radiation, and follow-up period. The effect measure used in conducting the selected systematic reviews was the number of implants that failed and the number of implants installed, not including details about the patient or radiation to answer the research question. Additionally, implant surface properties are confounding factors to be considered. In this sense, some studies (Chambrone et al., 2013; Chrcanovic et al., 2016; Smith Nobrega et al., 2016; In't Veld et al., 2021; Gupta et al., 2021) [64, 8, 63, 16, 62] sought to evaluate the geometry and surface treatment of the implant. However, it was not possible to reach a consensus on the risk of implant failures based on their characteristics because most individual studies did not report these data.

The main finding of this study was that, for patients after the diagnosis of head and neck cancer, the survival of implants that were irradiated before and/or after implantation was lower than that of implants installed in non-irradiated patients. We recommend that future randomized controlled trials be conducted on the topic. On the other hand, we consider the difficulty involved in conducting controlled and randomized experiments in this population. Therefore, new prospective and retrospective observational studies are needed. The literature is underexplored regarding the influence of radiotherapy on implants installed before the diagnosis of head and neck cancer, future research is also indicated in this regard.

## CONCLUSIONS

Despite weak evidence on dental implant failure in irradiated patients, it is important to evaluate the survival of implants installed before and/or after radiotherapy in head and neck cancer patients, which may improve the quality of life for these patients. Radiotherapy was associated with a higher percentage of dental implant losses, especially for implants installed in the maxilla and grafted bone. There are many biases in the selected systematic reviews, and the strength of evidence for analysis is weak. Therefore, the results of this umbrella review are not reliable to provide security in clinical decision-making. New systematic reviews with high methodological quality are necessary to increase the power of evidence in the success of implant installation in irradiated patients with head and neck cancer.

## OTHER INFORMATION

### Registration and Protocol

A detailed protocol was carried out before the beginning of this study and registered in the International Prospective Register of Systematic Reviews (PROSPERO) database under the identification number CRD42023406059.

### Funding

The author, Maria Cândida Dourado Pacheco, is a public servant at the Federal Institute of Northern Minas Gerais (IFNMG) - Januária campus. This study was carried out with the support for scientific publication from IFNMG, notice no. 1 of January 2, 2023.

### Conflict of Interest

The authors have no competing interests.

## REFERENCES


1. Jung RE, Zembic A, Pjetursson BE, Zwahlen M, Thoma DS. Systematic review of the survival rate and the incidence of biological, technical, and aesthetic complications of single crowns on implants reported in longitudinal studies with a mean follow-up of 5 years. Clin Oral Implants Res. 2012 Oct;23 Suppl 6:2-21. doi: 10.1111/j.1600-0501.2012.02547.x. PMID: 23062124. https://doi.org/10.1111/j.1600-0501.2012.02547.x

2. Papi P, Brauner E, Di Carlo S, Musio D, Tombolini M, De Angelis F, Valentini V, Tombolini V, Polimeni A, Pompa G. Crestal bone loss around dental implants placed in head and neck cancer patients treated with different radiotherapy techniques: a prospective cohort study. Int J Oral Maxillofac Surg. 2019 May;48(5):691-696. doi: 10.1016/j.ijom.2018.10.021. Epub 2018 Nov 22. PMID: 30471831. https://doi.org/10.1016/j.ijom.2018.10.021

3. Alberga JM, Korfage A, Bonnema I, Witjes MJH, Vissink A, Raghoebar GM. Mandibular dental implant placement immediately after teeth removal in head and neck cancer patients. Support Care Cancer. 2020 Dec;28(12):5911-5918. doi: 10.1007/s00520-020-05431-y. Epub 2020 Apr 11. PMID: 32279135; PMCID: PMC7686200. https://doi.org/10.1007/s00520-020-05431-y

4. Pieralli S, Spies BC, Schweppe F, Preissner S, Nelson K, Heiland M, Nahles S. Retrospective long-term clinical evaluation of implant-prosthetic rehabilitations after head and neck cancer therapy. Clin Oral Implants Res. 2021 Apr;32(4):470-486. doi: 10.1111/clr.13716. Epub 2021 Feb 7. PMID: 33501694. https://doi.org/10.1111/clr.13716

5. Li J, Feng K, Ye L, Liu Y, Sun Y, Wu Y. Influence of radiotherapy on dental implants placed in individuals before diagnosed with head and neck cancer: focus on implant-bed-specific radiation dosage. Clin Oral Investig. 2022 Sep;26(9):5915-5922. doi: 10.1007/s00784-022-04549-z. Epub 2022 May 16. PMID: 35578112. https://doi.org/10.1007/s00784-022-04549-z

6. Chrcanovic BR, Albrektsson T, Wennerberg A. Reasons for failures of oral implants. J Oral Rehabil. 2014 Jun;41(6):443-76. doi: 10.1111/joor.12157. Epub 2014 Mar 11. PMID: 24612346.

7. Do TA, Le HS, Shen YW, Huang HL, Fuh LJ. Risk Factors related to Late Failure of Dental Implant-A Systematic Review of Recent Studies. Int J Environ Res Public Health. 2020 Jun 2;17(11):3931. doi: 10.3390/ijerph17113931. PMID: 32498256; PMCID: PMC7312800.

8. Chrcanovic BR, Albrektsson T, Wennerberg A. Dental implants in irradiated versus nonirradiated patients: A meta-analysis. Head Neck. 2016 Mar;38(3):448-81. doi: 10.1002/hed.23875. Epub 2015 Jun 16. PMID: 25242560. https://doi.org/10.1002/hed.23875

9. Sung H, Ferlay J, Siegel RL, Laversanne M, Soerjomataram I, Jemal A, Bray F. Global Cancer Statistics 2020: GLOBOCAN Estimates of Incidence and Mortality Worldwide for 36 Cancers in 185 Countries. CA Cancer J Clin. 2021

May;71(3):209-249. doi: 10.3322/caac.21660. Epub 2021 Feb 4. PMID: 33538338. https://doi.org/10.3322/caac.21660

10. Schiegnitz E, Müller LK, Sagheb K, Theis L, Cagiran V, Kämmerer PW, Wegener J, Wagner W, Al-Nawas B. Clinical long-term and patient-reported outcomes of dental implants in oral cancer patients. Int J Implant Dent. 2021 Jul 13;7(1):93. doi: 10.1186/s40729-021-00373-4. PMID: 34255187; PMCID: PMC8276905.

11. Ernst N, Sachse C, Raguse JD, Stromberger C, Nelson K, Nahles S. Changes in Peri-Implant Bone Level and Effect of Potential Influential Factors on Dental Implants in Irradiated and Nonirradiated Patients Following Multimodal Therapy Due to Head and Neck Cancer: A Retrospective Study. J Oral Maxillofac Surg. 2016 Oct;74(10):1965-73. doi: 10.1016/j.joms.2016.06.005. Epub 2016 Jun 12. PMID: 27376183. https://doi.org/10.1016/j.joms.2016.06.005

12. Neckel N, Wagendorf P, Sachse C, Stromberger C, Vach K, Heiland M, Nahles S. Influence of implant-specific radiation doses on peri-implant hard and soft tissue: An observational pilot study. Clin Oral Implants Res. 2021 Feb;32(2):249-261. doi: 10.1111/clr.13696. Epub 2020 Dec 21. PMID: 33278849.

13. Wolf F, Spoerl S, Gottsauner M, Klingelhöffer C, Spanier G, Kolbeck C, Reichert TE, Hautmann MG, Ettl T. Significance of site-specific radiation dose and technique for success of implant-based prosthetic rehabilitation in irradiated head and neck cancer patients-A cohort study. Clin Implant Dent Relat Res. 2021 Jun;23(3):444-455. doi: 10.1111/cid.13005. Epub 2021 May 4. PMID: 33949108. https://doi.org/10.1111/cid.13005

14. Delanian S, Lefaix JL. The radiation-induced fibroatrophic process: therapeutic perspective via the antioxidant pathway. Radiother Oncol. 2004 Nov;73(2):119-31. doi: 10.1016/j.radonc.2004.08.021. PMID: 15542158.

15. Marx RE, Johnson RP. Studies in the radiobiology of osteoradionecrosis and their clinical significance. Oral Surg Oral Med Oral Pathol. 1987 Oct;64(4):379-90. doi: 10.1016/0030-4220(87)90136-8. PMID: 3477756. https://doi.org/10.1016/0030-4220(87)90136-8

16. In 't Veld M, Schulten EAJM, Leusink FKJ. Immediate dental implant placement and restoration in the edentulous mandible in head and neck cancer patients: a systematic review and meta-analysis. Curr Opin Otolaryngol Head Neck Surg. 2021 Apr 1;29(2):126-137. doi: 10.1097/MOO.0000000000000685. PMID: 33278135; PMCID: PMC7969163. https://doi.org/10.1097/MOO.0000000000000685

17. Schiegnitz E, Reinicke K, Sagheb K, König J, Al-Nawas B, Grötz KA. Dental implants in patients with head and neck cancer-A systematic review and meta-analysis of the influence of radiotherapy on implant survival. Clin Oral Implants Res. 2022 Oct;33(10):967-999. doi: 10.1111/clr.13976. Epub 2022 Aug 26. PMID: 35841367. https://doi.org/10.1111/clr.13976

18. Kende PP, Ranganath S, Landge JS, Sarda A, Wadewale M, Patil A, Singhavi HR. Survival of Dental Implants on Irradiated Jaws: A Systematic Review and Meta-

analysis. J Maxillofac Oral Surg. 2022 Sep;21(3):787-795. doi: 10.1007/s12663-022-01686-6. Epub 2022 Jan 15. PMID: 36274870; PMCID: PMC9474974. https://doi.org/10.1007/s12663-022-01686-6

19. Marques NP, Pérez-de-Oliveira ME, Normando AGC, Marques NCT, Epstein JB, Migliorati CA, Martelli-Júnior H, Ribeiro ACP, Rocha AC, Brandão TB, Sánchez FGV, Gueiros LAM, Lopes MA, Santos-Silva AR. Corrigendum to 'Clinical outcomes of dental implants in head and neck cancer patients: An overview'. Oral Surg Oral Med Oral Pathol Oral Radiol. 2023 Nov;136(5):663. doi: 10.1016/j.oooo.2023.07.014. Epub 2023 Jul 27. Erratum for: Oral Surg Oral Med Oral Pathol Oral Radiol. 2023 Jul;136(1):42-53. PMID: 37516619. https://doi.org/10.1016/j.oooo.2023.01.006

20. Zarzar AM, Sales PHDH, Barros AWP, Arreguy IMS, Carvalho AAT, Leão JC. Effectiveness of dental implants in patients undergoing radiotherapy for head and neck cancer: An umbrella review. Spec Care Dentist. 2024 Jan-Feb;44(1):40-56. doi: 10.1111/scd.12840. Epub 2023 Feb 28. PMID: 36852979. https://doi.org/10.1111/scd.12840

21. Page MJ, Moher D, Bossuyt PM, Boutron I, Hoffmann TC, Mulrow CD, Shamseer L, Tetzlaff JM, Akl EA, Brennan SE, Chou R, Glanville J, Grimshaw JM, Hróbjartsson A, Lalu MM, Li T, Loder EW, Mayo-Wilson E, McDonald S, McGuinness LA, Stewart LA, Thomas J, Tricco AC, Welch VA, Whiting P, McKenzie JE. PRISMA 2020 explanation and elaboration: updated guidance and exemplars for reporting systematic reviews. BMJ. 2021 Mar 29;372:n160. doi: 10.1136/bmj.n160. PMID: 33781993; PMCID: PMC8005925.

22. Ouzzani M, Hammady H, Fedorowicz Z, Elmagarmid A. Rayyan-a web and mobile app for systematic reviews. Syst Rev. 2016 Dec 5;5(1):210. doi: 10.1186/s13643-016-0384-4.

23. Landis JR, Koch GG. The measurement of observer agreement for categorical data. Biometrics. 1977 Mar;33(1):159-74. PMID: 843571.

24. Shea BJ, Reeves BC, Wells G, Thuku M, Hamel C, Moran J, Moher D, Tugwell P, Welch V, Kristjansson E, Henry DA. AMSTAR 2: a critical appraisal tool for systematic reviews that include randomised or non-randomised studies of healthcare interventions, or both. BMJ. 2017 Sep 21;358:j4008. doi: 10.1136/bmj.j4008. PMID: 28935701; PMCID: PMC5833365.

25. Benites Condezo AF, Araujo RZ, Koga DH, Curi MM, Cardoso CL. Hyperbaric oxygen therapy for the placement of dental implants in irradiated patients: systematic review and meta-analysis. Br J Oral Maxillofac Surg. 2021 Jul;59(6):625-632. doi: 10.1016/j.bjoms.2020.08.050. Epub 2020 Aug 19. PMID: 33952404. https://doi.org/10.1016/j.bjoms.2020.08.050

26. Schimmel M, Srinivasan M, McKenna G, Müller F. Effect of advanced age and/or systemic medical conditions on dental implant survival: A systematic review and meta-analysis. Clin Oral Implants Res. 2018 Oct;29 Suppl 16:311-330. doi: 10.1111/clr.13288. PMID: 30328186. https://doi.org/10.1111/clr.13288

27. Ravi P, Vaishnavi D, Gnanam A, Krishnakumar Raja VB. The role of hyperbaric oxygen therapy in the prevention and management of radiation-induced complications of the head and neck - a systematic review of literature. J Stomatol Oral Maxillofac Surg. 2017 Dec;118(6):359-362. doi: 10.1016/j.jormas.2017.07.005. Epub 2017 Aug 22. PMID: 28838774. https://doi.org/10.1016/j.jormas.2017.07.005

28. Zhang L, Ding Q, Liu C, Sun Y, Xie Q, Zhou Y. Survival, Function, and Complications of Oral Implants Placed in Bone Flaps in Jaw Rehabilitation: A Systematic Review. Int J Prosthodont. 2016 Mar-Apr;29(2):115-25. doi: 10.11607/ijp.4402. PMID: 26929947. https://doi.org/10.11607/ijp.4402

29. Shugaa-Addin B, Al-Shamiri HM, Al-Maweri S, Tarakji B. The effect of radiotherapy on survival of dental implants in head and neck cancer patients. J Clin Exp Dent. 2016 Apr 1;8(2):e194-200. doi: 10.4317/jced.52346. PMID: 27034761; PMCID: PMC4808316. https://doi.org/10.4317/jced.52346

30. Zen Filho EV, Tolentino Ede S, Santos PS. Viability of dental implants in head and neck irradiated patients: A systematic review. Head Neck. 2016 Apr;38 Suppl 1:E2229-40. doi: 10.1002/hed.24098. Epub 2015 Jul 15. PMID: 25926008.

31. Claudy MP, Miguens SA Jr, Celeste RK, Camara Parente R, Hernandez PA, da Silva AN Jr. Time interval after radiotherapy and dental implant failure: systematic review of observational studies and meta-analysis. Clin Implant Dent Relat Res. 2015 Apr;17(2):402-11. doi: 10.1111/cid.12096. Epub 2013 Jun 7. PMID: 23742098. https://doi.org/10.1111/cid.12096

32. Esposito M, Worthington HV. Interventions for replacing missing teeth: hyperbaric oxygen therapy for irradiated patients who require dental implants. Cochrane Database Syst Rev. 2013 Sep 30;2013(9):CD003603. doi: 10.1002/14651858.CD003603.pub3. PMID: 24085641; PMCID: PMC6787936.

33. Nooh N. Dental implant survival in irradiated oral cancer patients: a systematic review of the literature. Int J Oral Maxillofac Implants. 2013 Sep-Oct;28(5):1233-42. doi: 10.11607/jomi.3045. PMID: 24066313.

34. Esposito M, Grusovin MG, Patel S, Worthington HV, Coulthard P. Interventions for replacing missing teeth: hyperbaric oxygen therapy for irradiated patients who require dental implants. Cochrane Database Syst Rev. 2008 Jan 23;(1):CD003603. doi: 10.1002/14651858.CD003603.pub2. Update in: Cochrane Database Syst Rev. 2013;9:CD003603. PMID: 18254025.

35. Coulthard P, Esposito M, Worthington HV, Jokstad A. Interventions for replacing missing teeth: hyperbaric oxygen therapy for irradiated patients who require dental implants. Cochrane Database Syst Rev. 2002;(3):CD003603. doi: 10.1002/14651858.CD003603. Update in: Cochrane Database Syst Rev. 2008;(1):CD003603. PMID: 12137701.

36. Koudougou C, Bertin H, Lecaplain B, Badran Z, Longis J, Corre P, Hoornaert A. Postimplantation radiation therapy in head and neck cancer patients: Literature

review. Head Neck. 2020 Apr;42(4):794-802. doi: 10.1002/hed.26065. Epub 2020 Jan 3. PMID: 31898358. https://doi.org/10.1002/hed.26065

37. Thanya K, Ganapathy D. A review on dental implants in irradiated bones. Journal of Pharmaceutical Sciences and Research. 2016;8(7):635-637.

38. Tanaka TI, Chan HL, Tindle DI, Maceachern M, Oh TJ. Updated clinical considerations for dental implant therapy in irradiated head and neck cancer patients. J Prosthodont. 2013 Aug;22(6):432-8. doi: 10.1111/jopr.12028. Epub 2013 Feb 6. PMID: 23388045. https://doi.org/10.1111/jopr.12028

39. Dholam KP, Gurav SV. Dental implants in irradiated jaws: a literature review. J Cancer Res Ther. 2012 Jan;8 Suppl 1:S85-93. doi: 10.4103/0973-1482.92220. PMID: 22322737.

40. Javed F, Al-Hezaimi K, Al-Rasheed A, Almas K, Romanos GE. Implant survival rate after oral cancer therapy: a review. Oral Oncol. 2010 Dec;46(12):854-9. doi: 10.1016/j.oraloncology.2010.10.004. Epub 2010 Nov 4. PMID: 21055997. https://doi.org/10.1016/j.oraloncology.2010.10.004

41. Schoen PJ, Reintsema H, Raghoebar GM, Vissink A, Roodenburg JL. The use of implant retained mandibular prostheses in the oral rehabilitation of head and neck cancer patients. A review and rationale for treatment planning. Oral Oncol. 2004 Oct;40(9):862-71. doi: 10.1016/j.oraloncology.2003.08.024. PMID: 15380163. https://doi.org/10.1016/j.oraloncology.2003.08.024

42. Harrison JS, Stratemann S, Redding SW. Dental implants for patients who have had radiation treatment for head and neck cancer. Spec Care Dentist. 2003 Nov-Dec;23(6):223-9. doi: 10.1111/j.1754-4505.2003.tb00316.x. PMID: 15085959. https://doi.org/10.1111/j.1754-4505.2003.tb00316.x

43. Marx RE, Morales MJ. The use of implants in the reconstruction of oral cancer patients. Dent Clin North Am. 1998 Jan;42(1):177-202. PMID: 9421676.

44. Atanásio Pitorro TE, de Araújo Reis NT, Paranhos LR, Ferreira Soares PB. Survival of Dental Implants Placed Pre-Radiotherapy Versus Post-Radiotherapy in Native Bone: A Systematic Review. Int J Oral Maxillofac Implants. 2022 Nov-Dec;37(6):1100-1109. doi: 10.11607/jomi.9756. PMID: 36450015. https://doi.org/10.11607/jomi.9756

45. Estafanous E, Osswald M, Ellingsen J-E, Oates TW, Chvartszaid D, Stanford CM. Dental implant success: systematic reviews and clinical outcomes. International Journal of oral and Maxillofacial Implants. 2022;37(2):231-234.

46. Recani B, Dulcic N, Catic A, Cimic S. Influence of systemic diseases on the dental implants success rate: Review article. Research Journal of Pharmaceutical, Biological and Chemical Sciences. 2014;5(4):1188-1193.

47. Garg A, Guez G. Head and neck cancer, dental implants, and dental oncology. Dent Implantol Update. 2011 Jan;22(1):1-8. PMID: 21323003.

48. Hugentobler M, Guyot JP. Implants dentaires et radiothérapie [Dental implants and radiotherapy]. Rev Med Suisse. 2009 Sep 30;5(219):1936-9. French. PMID: 19946996.

49. Colella G, Cannavale R, Pentenero M, Gandolfo S. Oral implants in radiated patients: a systematic review. Int J Oral Maxillofac Implants. 2007 Jul-Aug;22(4):616-22. PMID: 17929523.

50. Samouhi P.; Buchbinder D. Rehabilitation of oral cancer patients with dental implants Current Opinion in Otolaryngology and Head and Neck Surgery. 2000;8(4):305-313. doi: 10.1097/00020840-200008000-00006

51. Lu B, Zhang X, Liu B. A systematic review and meta-analysis on influencing factors of failure of oral implant restoration treatment. Ann Palliat Med. 2021 Dec;10(12):12664-12677. doi: 10.21037/apm-21-3449. PMID: 35016469. https://doi.org/10.21037/apm-21-3449

52. Kotsakis GA, Ioannou AL, Hinrichs JE, Romanos GE. A systematic review of observational studies evaluating implant placement in the maxillary jaws of medically compromised patients. Clin Implant Dent Relat Res. 2015 Jun;17(3):598-609. doi: 10.1111/cid.12240. Epub 2014 May 30. PMID: 24889264. https://doi.org/10.1111/cid.12240

53. Chen H, Liu N, Xu X, Qu X, Lu E. Smoking, radiotherapy, diabetes and osteoporosis as risk factors for dental implant failure: a meta-analysis. PLoS One. 2013 Aug 5;8(8):e71955. doi: 10.1371/journal.pone.0071955. PMID: 23940794; PMCID: PMC3733795. https://doi.org/10.1371/journal.pone.0071955

54. Toneatti DJ, Graf RR, Burkhard JP, Schaller B. Survival of dental implants and occurrence of osteoradionecrosis in irradiated head and neck cancer patients: a systematic review and meta-analysis. Clin Oral Investig. 2021 Oct;25(10):5579-5593. doi: 10.1007/s00784-021-04065-6. Epub 2021 Aug 16. PMID: 34401944; PMCID: PMC8443505. https://doi.org/10.1007/s00784-021-04065-6

55. Panchal H, Shamsunder MG, Petrovic I, Rosen EB, Allen RJ Jr, Hernandez M, Ganly I, Boyle JO, Matros E, Nelson JA. Dental Implant Survival in Vascularized Bone Flaps: A Systematic Review and Meta-Analysis. Plast Reconstr Surg. 2020 Sep;146(3):637-648. doi: 10.1097/PRS.0000000000007077. PMID: 32459736; PMCID: PMC8435212. https://doi.org/10.1097/PRS.0000000000007077

56. Korfage A, Dijkstra PU, Roodenburg JL, Reintsema H, Raghoebar GM, Vissink A. Dental implants in irradiated patients: which factors influence implant survival? Clin Oral Investig. 2015 Sep;19(7):1689-90. doi: 10.1007/s00784-015-1435-2. Epub 2015 Mar 5. PMID: 25735845. https://doi.org/10.1007/s00784-015-1435-2

57. Schiegnitz E, Al-Nawas B, Kämmerer PW, Grötz KA. Dental implants in irradiated patients: which factors influence implant survival? Clin Oral Investig. 2015 Sep;19(7):1691-2. doi: 10.1007/s00784-015-1480-x. Epub 2015 Apr 24. PMID: 25903062. https://doi.org/10.1007/s00784-015-1480-x

58. Barber AJ, Butterworth CJ, Rogers SN. Systematic review of primary osseointegrated dental implants in head and neck oncology. Br J Oral Maxillofac Surg. 2011 Jan;49(1):29-36. doi: 10.1016/j.bjoms.2009.12.007. Epub 2010 Jan 15. PMID: 20079957. https://doi.org/10.1016/j.bjoms.2009.12.007

59. Camolesi GCV, Veronese HRM, Celestino MA, Blum DFC, Márquez-Zambrano JA, Carmona-Pérez FA, Jara-Venegas TA, Pellizzon ACA, Bernaola-Paredes WE. Survival of osseointegrated implants in head and neck cancer patients submitted to multimodal treatment: a systematic review and meta-analysis. Support Care Cancer. 2023 Oct 18;31(12):641. doi: 10.1007/s00520-023-08088-5. PMID: 37851170.

60. Shahi A, Jebeli ZST, Yousefi H, Fazeli F, Fotovat F. Evaluation of the Success Rate of Dental Implants in Oral Cavity Cancers: A Systematic Review and Meta-analysis. Academic Journal of Health Sciences. 2023 Mar;38(2):54-59. doi: 10.3306/AJHS.2023.38.02.54.

61. Shokouhi B, Cerajewska T. Radiotherapy and the survival of dental implants: a systematic review. Br J Oral Maxillofac Surg. 2022 May;60(4):422-429. doi: 10.1016/j.bjoms.2021.09.006. Epub 2021 Sep 16. PMID: 34903387. https://doi.org/10.1016/j.bjoms.2021.09.006

62. Gupta S, Mortellaro C, Panda S, Rovati M, Giacomello MS, Colletti L, Greco Lucchina A, Del Fabbro M. Dental implant survival rate in irradiated and non-radiated patients: a systematic review and meta-analysis. J Biol Regul Homeost Agents. 2021 Mar-Apr;35(2 Suppl. 1):53-65. doi: 10.23812/21-2supp1-5. PMID: 34281302. https://doi.org/10.23812/21-2supp1-5

63. Smith Nobrega A, Santiago JF Jr, de Faria Almeida DA, Dos Santos DM, Pellizzer EP, Goiato MC. Irradiated patients and survival rate of dental implants: A systematic review and meta-analysis. J Prosthet Dent. 2016 Dec;116(6):858-866. doi: 10.1016/j.prosdent.2016.04.025. Epub 2016 Jul 25. PMID: 27460315. https://doi.org/10.1016/j.prosdent.2016.04.025

64. Chambrone L, Mandia J Jr, Shibli JA, Romito GA, Abrahao M. Dental implants installed in irradiated jaws: a systematic review. J Dent Res. 2013 Dec;92(12 Suppl):119S-30S. doi: 10.1177/0022034513504947. Epub 2013 Oct 24. PMID: 24158336; PMCID: PMC3860067. https://doi.org/10.1177/0022034513504947

65. Schiegnitz E, Al-Nawas B, Kämmerer PW, Grötz KA. Oral rehabilitation with dental implants in irradiated patients: a meta-analysis on implant survival. Clin Oral Investig. 2014 Apr;18(3):687-98. doi: 10.1007/s00784-013-1134-9. Epub 2013 Nov 24. Erratum in: Clin Oral Investig. 2015 Sep;19(7):1693. PMID: 24271500. https://doi.org/10.1007/s00784-013-1134-9

66. Anderson L, Meraw S, Al-Hezaimi K, Wang HL. The influence of radiation therapy on dental implantology. Implant Dent. 2013 Feb;22(1):31-8. doi: 10.1097/ID.0b013e31827e84ee. PMID: 23287974.

67. Di Carlo S, De Angelis F, Ciolfi A, Quarato A, Piccoli L, Pompa G, Brauner E. Timing for implant placement in patients treated with radiotherapy of head and

neck. Clin Ter. 2019 Sep-Oct;170(5):e345-e351. doi: 10.7417/CT.2019.2153. PMID: 31612191. https://doi.org/10.7417/CT.2019.2153

68. Patel J, Antov H, Nixon P. Implant-supported oral rehabilitation in oncology patients: a retrospective cohort study. Br J Oral Maxillofac Surg. 2020 Oct;58(8):1003-1007. doi: 10.1016/j.bjoms.2020.05.016. Epub 2020 May 27. PMID: 32474015. https://doi.org/10.1016/j.bjoms.2020.05.016

Table 1 – PICO formulation

| | | |
|---|---|---|
| P | Population | Patients undergoing radiotherapy for head and neck cancer |
| I | Intervention | Installation of dental implants before and/or after radiotherapy |
| C | Comparison | Patients with head and neck cancer |
| O | Outcome | Survival of dental implants |

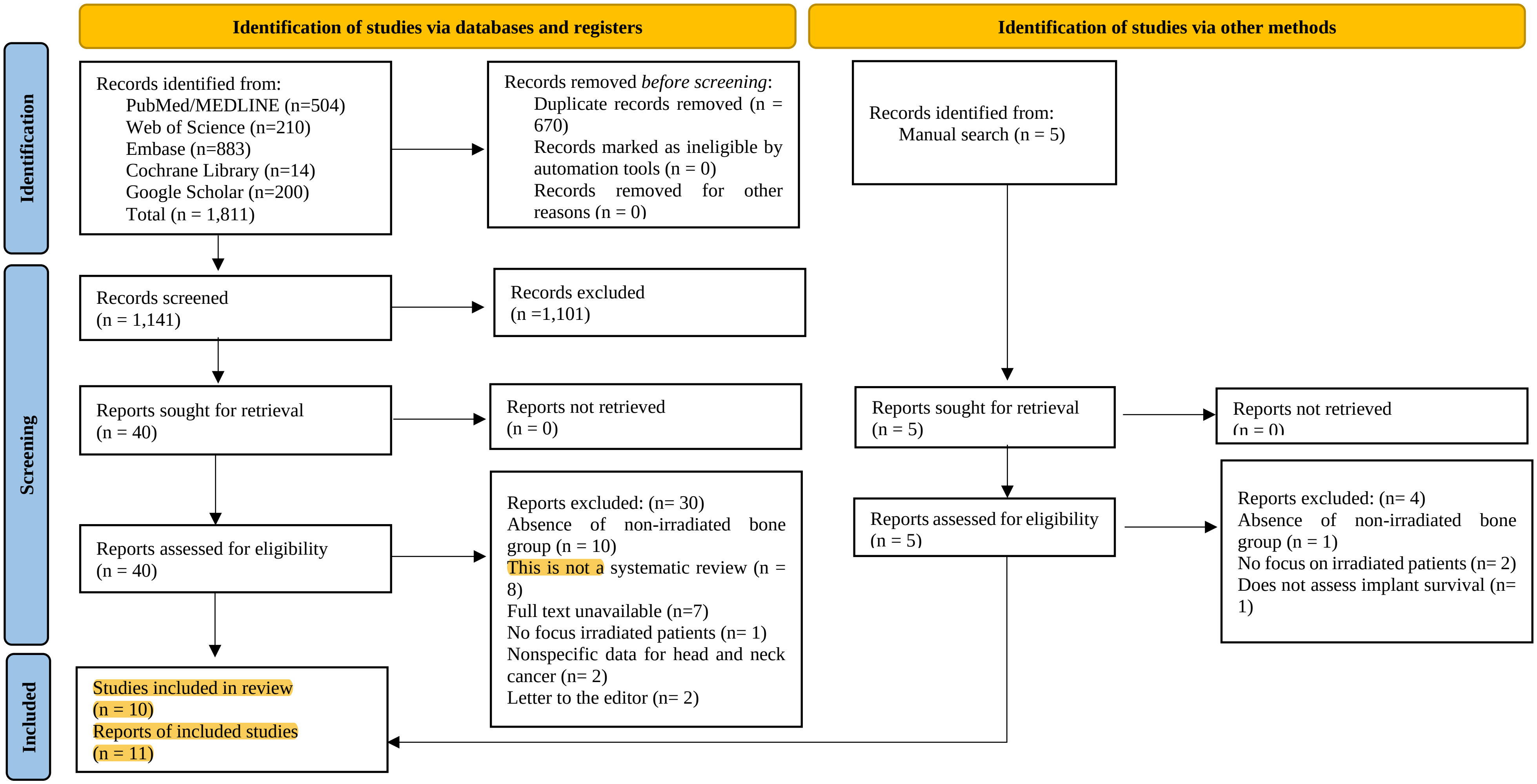


Figure 1 – PRISMA 2020 flow diagram for new systematic reviews which included searches of databases, registers, and other sources.

Table 2 – General characteristics of the included systematics reviews (part 1)

| Study (year) | N Participants / Age range (years) | Country | N Primary studies / N Study design | Presence of meta-analysis | Radiation dose range (Gy) / Time of implant placement | *N Failed / placed implants | Implantation site | Interval between RT and implant placement, months | Follow-up, mo |
|---|---|---|---|---|---|---|---|---|---|
| Camolesi et al. (2023) | 876 / 31,6% (> 60) | Espanha | 19 / PS:2 RS:17 | Yes | 17 S (≥ 50 Gy) 2 S (< 50 Gy) / Post RT | Overall: 4,473 | 1 S (Mx) 7 S (Md) 8 S (Mx/md) NB/GB | 6-12 Pot RT 13-20 Posr RT >21 Post RT | 5 S (>5 years) |
| Shahi et al (2023) | 726 / 50,93 mean | Iran | 13 / RS: 13 | Yes | NM / Post RT | 136/1,226(EG) 41/932(CG) | Mx/md | NM | NM |
| Kende et al. (2022) | 1,246 / 51.43 mean | India | 23 / RS:21 PS:2 | Yes | 40-72 / **Pre and post RT | 364/2,186 (EG) 179/1,685 (CG) | Mx/md NB/GB | NM | 52.5 mean |
| Schiegnitz et al. (2022) | 3,445 / 6-91 | Germany | 59 / PS:12 RS:47 CSS:1 | Yes | 20-81,6 / Pre and post RT | 373/1,825(EG) 278/2,077 (CG) | Mx/md NB/GB | NM | 3-120 |
| Shokouhi and Cerajewska (2022) | 441 / 28-88 | United Kingdom | 07 / PS:4 RS:3 | Yes | 10-72 / Post RT | 62/508(EG) 13/452 (CG) | Mx/md NB | NM | 12-168 |
| Gupta et al. (2021) | 1,097 / 13-89 | Italy | 15 / RS:5 PS:10 | Yes | 20-72 / Post RT | 323/2,203 (EG) 141/2,349 (CG) | Mx/md NB/GB | ***6-41 Post RT | 6-120 |
| In't Veld et al. (2021) | 1,148 / 17-91 | Netherlands | 10 / PS:6 RS:4 | Yes | 30-72 / Pre RT | 37/413 (EG) 5/372 (CG) | Md/mx NB/GB | Not performed | 12-174 |
| Smith Nobrega et al. (2016) | 2,220 / 58 mean | Brazil | 40 / PS:18 RS:21 RCT:1 | Yes | 10-145 / Pre and post RT | 629/3,773 (EG) 191/3,228 (CG) | Mx/md NB/GB | ***1-240 Post RT | 1-276 |
| Chrcanovic et al. (2016) | 4,431 / 6-94 | Sweden | 54 / RS:44 CCT:10 | Yes | 21-120 / Pre and post RT | 640/3,914 (EG) 684/14,514 (CG) | Mx/md NB/GB | <12 Post RT ≥12 Post RT | Up to 180 |
| Chambrone et al. (2013) | 563 / NM | Brazil | 15 / RS:11 PS:2 RCT:2 | Yes | NM / Pre and post RT | 211/1,689 (EG) 305/8,461 (CG) | Mx/md NB | NM | Up to 181 |

Abbreviations: CG: control group (nonirradiated patients); CCT: controlled clinical trial; CS:cross sectional study; EG: experimental group (irradiated patients); GB: grafted bone; mo: months; N: number; NB: native bone; Mx/md: maxilla and mandible; NM: not mentioned; PS: prospective study; RS: retrospective study; RCT: randomized controlled trial; RT: radiotherapy; S: studies.

* Numbers reported in meta-analyses including data from the longest follow-up period.

** Implants placed during tumor ablative surgery prior to radiotherapy and implants placed after completion of radiotherapy.

*** Variation in primary studies.

Table 3 – General characteristics of the included systematics reviews (part 2)

| Study (year) | Implant characteristics | Characteristics of cancer | Adjuvant therapy | Bone crest stability | Postoperative complications | Prosthetic rehabilitation |
|---|---|---|---|---|---|---|
| Camolesi et al. (2023) | NM | Head and neck cancer | 8 S (CT+ surgery) | NM | 7 S (PI)<br>5 S (ORN) | NM |
| Shahi et al. (2023) | NM | Oral cancer | NM | NM | NM | NM |
| Kende et al. (2022) | NM | Oral cancer | NM | NM | NM | NM |
| Schiegnitz et al. (2022) | NM | Head and neck cancer | CT<br>HBO<br>AT | NM | ORN, PI, Mucositis | NM |
| Shokouhi and Cerajewska (2022) | NM | Head and neck cancer | NM | NM | ORN | NM |
| Gupta et al. (2021) | Branemark, Frialit, TPS, SLA, ITI, IMZ, Xive, Astra tech, Steri-Oss, Camlog, Osteotite implants | NM | HBO<br>AT | NM | NM | NM |
| In't Veld et al. (2021) | Osseospeed/Astra tech, Branemark machined surface or a Ti-Unite surface/Nobel Biocare, Branemark MK II/III 2-phase implants/Nobel Biocare, Frialit 2-phase implants/Nobel Biocare, ITI/Straumann, Neoss/Neoss implant | Head and neck cancer | HBO | NM | ORN, PI, LO, Tumor recurrence | Overdenture |
| Smith Nobrega et al. (2016) | Astra tech, Nobel biocare, Serf, Friatec, Lifecore biomedical, Straumann, Osseous-mozograu, Sterio Os, Camlog, Friadent, Interpore, Internatonal, Imtec, Dyna, Screw-vent | NM | HBO | NM | ORN, PI | NM |
| Chrcanovic et al. (2016) | Branemark, Bonefit, IMZ, Frialit-2 system, Astra, Bonefit, Steri-Oss, ITI, Ankylos, Dyna, Ciny, SLA, CAMLOG, Branemark MKII, MKIII, Endopore, MG Osseus-mozograu, TiUnite, Neoss | Head and neck cancer | CT<br>AT<br>MR<br>HBO | MBL | Postoperative infection | Fixed overdenture |
| Chambrone et al. (2013) | Branemark, TPS, SLA, SLActive | Head cancer | HBO | NM | NM | NM |

Abbreviations: AT: antibiotic therapy, BOT; base of tongue, CT: chemortherapy, DM: dorsal maxila, FOM: floor of mouth, HBO: hyperbaric oxygen therapy, LO: lack of osseointegration, LP: laryngopharynx, MBL: marginal bone loss, MR: mouth rinse, NM: not mentioned, NP: nasopharynx, OP: oropharynx, ORN: osteoradionecrosis, PI: peri-implantitis, S: studies; SLA: sandblasted and acid etched, SLActive: modified sandblasted and acid etched, TPS: titanium plasma sprayed.

Table 4 – Summary of meta-analyses on implant failures in irradiated versus non-irradiated patients

| Meta-analysis | N Failed/placed implants | Results | N Studies Survival rate (%) |
|---|---|---|---|
| Camolesi et al. (2023) 5 years of follow-up | NM | WP (EG): 93.13%; CI 95%: 87.20-99.6; *p*<0.001; $I^2$=85% WP (CG): 98.52%; CI 95%: 97.56-99.48; *p*<0.001; $I^2$=0% | S: 3 93.13(EG) 98.56(CG) |
| Shahi et al. (2023) follow-up not mentioned | 136/1,226(EG) 41/932(CG) | OR: 1.79; CI 95%: 1.35-2.23; *p*<0.01; $I^2$=62,89% | S: 9 81.35(EG) 87.45(CG) |
| Kende et al. (2022) mean 52.5 months of follow-up | 364/2,186(EG) 179/1,685(CG) | OR: 0.24; CI 95%: 0.14-0.40; *p*<0.01; $I^2$=73% | S: 16 82.47(EG) 89.37(CG) |
| Schiegnitz et al. (2022) (2013-2021) ≥ 3 years of follow-up | NM | OR: 2.07; CI 95%: 1.54-2.97; *p*<0,00001 | NM |
| Schiegnitz et al. (2022) (2013-2021) ≥ 5 years of follow-up | NM | OR: 1.8; CI 95%: 1.21–2.67; *p*=0.003 | NM |
| Schiegnitz et al. (2022) (1990-2021) 3 years of follow-up | 500/3,269(EG) 339/3,348(CG) | OR: 2.06; CI 95%: 1.75-2.42; *p*<0.00001; $I^2$=33% | S: 21 79.56(EG) 86.61(CG) |
| Schiegnitz et al. (2022) (1990 - 2021) 5 years of follow-up | 373/1,825(EG) 278/2,077(CG) | OR: 1.97; CI 95%:1.63-2.37; *p*<0.00001; $I^2$=47% | S: 12 84.70(EG) 89.87(CG) |
| Shokouhi and Cerajewska (2022) 1-3.8 years of follow-up | 62/508(EG) 13/452(CG) | OR: 4.77; CI 95%: 2.57-8.89; *p*<0.00001; $I^2$=0% | S: 4 87.79(EG) 97.12(CG) |
| Gupta et al. (2021) 6-12 months of follow-up | 323/2,203(EG) 141/2,349(CG) | OR: 2.95; CI: 1.93-4.50; *p*<0.00001; $I^2$=50% | S: 14 85.80(EG) 93.90(CG) |
| In't Veld et al. (2021) follow-up not mentioned | 37/473(EG) 5/372(CG) | RR: 5.02; CI 95%: 0.92-27.38; *p*=0.06; $I^2$=56% | S: 3 92.17(EG) 98.65(CG) |
| Smith Nobrega et al. (2016) 16 years of folow-up | 629/3,773(EG) 191/3,328(CG) | RR: 2.63; CI 95%: 1.93-3.58; *p*<0.001; $I^2$=56% | S: 26 84.30(EG) 94.26(CG) |
| Chrcanovic et al. (2016) follow-up not mentioned | 640/3,914(EG) 684/14,514(CG) | RR: 2.18; CI 95%: 1.71-2.79; *p*<0.00001; $I^2$=52% | S: 37 83.64(CG) 95.29(EG) |
| Shiegnitz et al. (2014) (2007-2013) ≥ 5 years of follow-up | 19/263(EG) 13/241(CG) | OR: 1.44; CI 95%: 0.67-3.10; *p*=0,35; $I^2$=75% | S: 3 92.77(EG) 94.60(CG) |
| Shiegnitz et al. (2014) (1990-2006) ≥ 5 years of follow-up | 281/983(EG) 203/1,040(CG) | OR: 2.11; CI 95%: 1.69-2.65; *p*<0.00001; $I^2$=72% | S: 4 71.41(EG) 80.48(CG) |
| Chambrone et al. (2013) follow-up not mentioned | 110/549(EG) 293/8,383(CG) | RR: 2.74; CI 95%: 1.86-4.05; *p*<0.00001; $I^2$=0% | S: 7 83.30(EG) 93.81(CG) |

Abbreviations: CG: control group (nonirradiated patients); CI: confidence interval; EG: experimental group (irradiated patients); N: number; NM: not mentioned; OR: odds ratio; RR: risk ratio; S: studies; WP: weighted proportion

Table 5 - Assessment of the methodological quality of systematic reviews using the AMSTAR 2 tool

| Systematic review | Q.1 | Q.2 | Q.3 | Q.4 | Q.5 | Q.6 | Q.7 | Q.8 | Q.9 | Q.10 | Q.11 | Q.12 | Q.13 | Q.14 | Q.15 | Q.16 | Overall classification |
|---|---|---|---|---|---|---|---|---|---|---|---|---|---|---|---|---|---|
| Camolesi et al. (2023) | Y | Y | Y | N | Y | Y | N | Y | Y | N | Y | Y | N | Y | Y | N | Critically low |
| Shahi et al. (2023) | Y | N | Y | N | N | N | N | N | Y | N | Y | N | N | Y | Y | Y | Critically low |
| Kende et al. (2022) | Y | Y | Y | N | Y | Y | N | N | Y | N | N | N | N | N | N | Y | Critically low |
| Schiegnitz et al. (2022) | N | PY | Y | N | N | N | N | Y | Y | N | Y | Y | Y | Y | Y | Y | Critically low |
| Shokouhi and Cerajewska (2022) | Y | N | Y | PY | N | N | N | Y | Y | N | Y | Y | Y | Y | N | Y | Critically low |
| Gupta et al. (2021) | Y | N | Y | N | Y | Y | N | Y | Y | N | Y | Y | N | Y | N | Y | Critically low |
| In't Veld et al. (2021) | Y | N | N | N | Y | N | N | Y | N | N | Y | Y | Y | Y | N | Y | Critically low |
| Smith Nobrega et al. (2016) | Y | N | Y | N | Y | Y | N | Y | N | N | Y | Y | Y | Y | Y | N | Critically low |
| Chrcanovic et al. (2016) | Y | N | Y | PY | Y | N | N | Y | N | N | Y | Y | Y | Y | Y | N | Critically low |
| Shiegnitz et al. (2014) | N | N | Y | N | Y | Y | N | Y | N | N | Y | N | Y | Y | Y | N | Critically low |
| Chambrone et al. (2013) | Y | N | Y | PY | Y | Y | Y | Y | Y | N | Y | Y | Y | Y | N | Y | Critically low |

Abbreviations: Y: yes, N: not, PY: Partially yes

Q.1. Did the research questions and inclusion criteria for the review include the components of PICO? Q.2. Did the report of the review contain an explicit statement that the review methods were established prior to the conduct of the review and did the report justify any significant deviations from the protocol? Q. 3. Did the review authors explain their selection of the study designs for inclusion in the review? Q. 4. Did the review authors use a comprehensive literature search strategy? Q. 5. Did the review authors perform study selection in duplicate? Q. 6. Did the review authors perform data extraction in duplicate? Q. 7. Did the review authors provide a list of excluded studies and justify the exclusions? Q. 8. Did the review authors describe the included studies in adequate detail? Q. 9. Did the review authors use a satisfactory technique for assessing the risk of bias (RoB) in individual studies that were included in the review? Q. 10. Did the review authors report on the sources of funding for the studies included in the review? Q. 11. If meta-analysis was performed, did the review authors use appropriate methods for statistical combination of results? Q. 12. If meta-analysis was performed, did the review

authors assess the potential impact of RoB in individual studies on the results of the meta-analysis or Other evidence synthesis? Q. 13. Did the review authors account for RoB in primary studies when interpreting/discussing the results of the review? Q. 14. Did the review authors provide a satisfactory explanation for, and discussion of, any heterogeneity observed in the results of the review? Q. 15. If they performed quantitative synthesis did the review authors carry out an adequate investigation of publication bias (small study bias) and discuss its likely impact on the results of the review? Q.16. Did the review authors report any potential sources of conflict of interest, including any funding they received for conducting the review?

**SUPPORTING INFORMATION**

S1 – Search strategy in electronic databases

| Electronic databases | Search strategy |
|---|---|
| PubMed | ("dental implants"[MeSH Terms] OR ("dental"[All Fields] AND "implants"[All Fields]) OR "dental implants"[All Fields] OR ("dental implants"[MeSH Terms] OR ("dental"[All Fields] AND "implants"[All Fields]) OR "dental implants"[All Fields] OR ("dental"[All Fields] AND "implant"[All Fields]) OR "dental implant"[All Fields]) OR ("dental implantation"[MeSH Terms] OR ("dental"[All Fields] AND "implantation"[All Fields]) OR "dental implantation"[All Fields]) OR ("dental prosthesis, implant supported"[MeSH Terms] OR ("dental"[All Fields] AND "prosthesis"[All Fields] AND "implant supported"[All Fields]) OR "implant-supported dental prosthesis"[All Fields] OR ("implant"[All Fields] AND "supported"[All Fields] AND "dental"[All Fields] AND "prosthesis"[All Fields]) OR "implant supported dental prosthesis"[All Fields]) OR ("dental implantation, endosseous"[MeSH Terms] OR ("dental"[All Fields] AND "implantation"[All Fields] AND "endosseous"[All Fields]) OR "endosseous dental implantation"[All Fields] OR ("endosseous"[All Fields] AND "dental"[All Fields] AND "implantation"[All Fields])) OR (("dental implants"[MeSH Terms] OR ("dental"[All Fields] AND "implants"[All Fields]) OR "dental implants"[All Fields] OR ("dental"[All Fields] AND "implant"[All Fields]) OR "dental implant"[All Fields]) AND ("mortality"[MeSH Subheading] OR "mortality"[All Fields] OR "survival"[All Fields] OR "survival"[MeSH Terms] OR "survivability"[All Fields] OR "survivable"[All Fields] OR "survivals"[All Fields] OR "survive"[All Fields] OR "survived"[All Fields] OR "survives"[All Fields] OR "surviving"[All Fields])) OR ("dental implants"[MeSH Terms] OR ("dental"[All Fields] AND "implants"[All Fields]) OR "dental implants"[All Fields] OR ("surgical"[All Fields] AND "dental"[All Fields] AND "prostheses"[All Fields])) OR ("dental implants"[MeSH Terms] OR ("dental"[All Fields] AND "implants"[All Fields]) OR "dental implants"[All Fields] OR ("surgical"[All Fields] AND "dental"[All Fields] AND "prosthesis"[All Fields]) OR "surgical dental prosthesis"[All Fields]) OR (("teeth s"[All Fields] OR "teeths"[All Fields] OR "tooth"[MeSH Terms] OR "tooth"[All Fields] OR "teeth"[All Fields] OR "tooth s"[All Fields] OR "tooths"[All Fields]) AND ("drug implants"[MeSH Terms] OR ("drug"[All Fields] AND "implants"[All Fields]) OR "drug implants"[All Fields] OR "implant"[All Fields] OR "embryo implantation"[MeSH Terms] OR ("embryo"[All Fields] AND "implantation"[All Fields]) OR "embryo implantation"[All Fields] OR "implantation"[All Fields] OR "implant s"[All Fields] OR "implantability"[All Fields] OR "implantable"[All Fields] OR "implantables"[All Fields] OR "implantate"[All Fields] OR "implantated"[All Fields] OR "implantates"[All Fields] OR "implantations"[All Fields] OR "implanted"[All Fields] OR "implanter"[All Fields] OR "implanters"[All Fields] OR "implanting"[All Fields] OR "implantion"[All Fields] OR "implantitis"[All Fields] OR "implants"[All Fields]))) AND ("head and neck neoplasms"[MeSH Terms] OR ("head"[All Fields] AND "neck"[All Fields] AND "neoplasms"[All Fields]) OR "head and neck neoplasms"[All Fields] OR ("head"[All Fields] AND "neck"[All Fields] AND "cancer"[All Fields]) OR "head and neck cancer"[All Fields] OR (("head neck"[Journal] OR ("head"[All Fields] AND "and"[All Fields] AND "neck"[All Fields]) OR "head and neck"[All Fields]) AND ("cysts"[MeSH Terms] OR "cysts"[All Fields] OR "cyst"[All Fields] OR "neurofibroma"[MeSH Terms] OR "neurofibroma"[All Fields] OR "neurofibromas"[All Fields] OR "tumor s"[All Fields] OR "tumoral"[All Fields] OR "tumorous"[All Fields] OR "tumour"[All Fields] OR "neoplasms"[MeSH Terms] OR "neoplasms"[All Fields] OR "tumor"[All Fields] OR "tumour s"[All Fields] OR "tumoural"[All Fields] OR "tumourous"[All Fields] OR "tumours"[All Fields] OR "tumors"[All Fields])) OR ("head and neck neoplasms"[MeSH Terms] OR ("head"[All Fields] AND "neck"[All Fields] AND "neoplasms"[All Fields]) OR "head and neck neoplasms"[All Fields]) OR ("head neck oncol"[Journal] OR ("head"[All Fields] AND "and"[All Fields] AND "neck"[All Fields] AND "oncology"[All Fields]) OR "head and neck oncology"[All Fields]) OR ("mouth neoplasms"[MeSH Terms] OR ("mouth"[All Fields] AND "neoplasms"[All Fields]) OR "mouth neoplasms"[All Fields]) OR ("mouth neoplasms"[MeSH Terms] OR ("mouth"[All Fields] AND "neoplasms"[All Fields]) OR "mouth neoplasms"[All Fields] OR ("oral"[All Fields] AND "cancer"[All Fields]) OR "oral cancer"[All Fields])) AND ("radiotherapy"[MeSH Subheading] OR "radiotherapy"[All Fields] OR ("radiation"[All Fields] AND "therapy"[All Fields]) OR "radiation therapy"[All Fields] OR "radiotherapy"[MeSH Terms] OR ("radiation"[All Fields] AND "therapy"[All Fields]) OR "radiation therapy"[All |

| | |
|---|---|
| | Fields] OR ("radiotherapy"[MeSH Terms] OR "radiotherapy"[All Fields] OR ("radiation"[All Fields] AND "therapies"[All Fields]) OR "radiation therapies"[All Fields]) OR ("radiotherapy"[MeSH Terms] OR "radiotherapy"[All Fields] OR "radiotherapies"[All Fields] OR "radiotherapy"[MeSH Subheading] OR "radiotherapy s"[All Fields]) OR ("radiotherapy"[MeSH Terms] OR "radiotherapy"[All Fields] OR "radiotherapies"[All Fields] OR "radiotherapy"[MeSH Subheading] OR "radiotherapy s"[All Fields]) OR ("irradiance"[All Fields] OR "irradiances"[All Fields] OR "irradiant"[All Fields] OR "irradiates"[All Fields] OR "irradiating"[All Fields] OR "irradiations"[All Fields] OR "irradiative"[All Fields] OR "irradiator"[All Fields] OR "irradiators"[All Fields] OR "radiotherapy"[MeSH Terms] OR "radiotherapy"[All Fields] OR "irradiate"[All Fields] OR "irradiated"[All Fields] OR "irradiation"[All Fields]) OR (("irradiance"[All Fields] OR "irradiances"[All Fields] OR "irradiant"[All Fields] OR "irradiates"[All Fields] OR "irradiating"[All Fields] OR "irradiations"[All Fields] OR "irradiative"[All Fields] OR "irradiator"[All Fields] OR "irradiators"[All Fields] OR "radiotherapy"[MeSH Terms] OR "radiotherapy"[All Fields] OR "irradiate"[All Fields] OR "irradiated"[All Fields] OR "irradiation"[All Fields]) AND ("patient s"[All Fields] OR "patients"[MeSH Terms] OR "patients"[All Fields] OR "patient"[All Fields] OR "patients s"[All Fields]))) |
| Embase | ((('dental'/exp OR dental) AND ('implants'/exp OR implants)) OR (('dental'/exp OR dental) AND ('implant'/exp OR implant)) OR (('dental'/exp OR dental) AND ('implantation'/exp OR implantation)) OR ('implant supported' AND ('dental'/exp OR dental) AND ('prosthesis'/exp OR prosthesis)) OR (endosseous AND ('dental'/exp OR dental) AND ('implantation'/exp OR implantation)) OR (('dental'/exp OR dental) AND ('implant'/exp OR implant) AND ('survival'/exp OR survival)) OR (surgical AND ('dental'/exp OR dental) AND ('prostheses'/exp OR prostheses)) OR (surgical AND ('dental'/exp OR dental) AND ('prosthesis'/exp OR prosthesis)) OR (('tooth'/exp OR tooth) AND ('implant'/exp OR implant))) AND ((('head'/exp OR head) AND ('neck'/exp OR neck) AND ('cancer'/exp OR cancer)) OR (('head'/exp OR head) AND ('neck'/exp OR neck) AND ('tumors'/exp OR tumors)) OR (('head'/exp OR head) AND ('neck'/exp OR neck) AND ('neoplasms'/exp OR neoplasms)) OR (('head'/exp OR head) AND ('neck'/exp OR neck) AND ('oncology'/exp OR oncology)) OR (('mouth'/exp OR mouth) AND ('neoplasms'/exp OR neoplasms)) OR (oral AND ('cancer'/exp OR cancer))) AND ((('radiation'/exp OR radiation) AND ('therapy'/exp OR therapy)) OR (('radiation'/exp OR radiation) AND therapies) OR ('radiotherapy'/exp OR radiotherapy) OR radiotherapies OR ('irradiation'/exp OR irradiation) OR (('irradiation'/exp OR irradiation) AND ('patients'/exp OR patients))) |
| Cochrane Library | dental implants OR dental implant OR dental implantation OR implant-supported dental prosthesis OR endosseous dental implantation OR dental implant survival OR surgical dental prostheses OR surgical dental prosthesis OR tooth implant AND head and neck cancer OR head and neck tumors OR head and neck neoplasms OR head and neck Oncology OR mouth neoplasms OR oral cancer AND radiation therapy OR radiation therapies OR radiotherapy OR radiotherapies OR irradiation OR irradiation patients |
| Web of Science | ((((((ALL=(dental implants OR dental implant OR dental implantation OR implant-supported dental prosthesis OR endosseous dental implantation OR dental implant survival OR surgical dental prostheses OR surgical dental prosthesis OR tooth implant AND head and neck cancer OR head and neck tumors OR head and neck neoplasms OR head and neck Oncology OR mouth neoplasms OR oral cancer AND radiation therapy OR radiation therapies OR radiotherapy OR radiotherapies OR irradiation OR irradiation patients)) AND ALL=(dental implant)) AND ALL=(radiotherapy)) AND ALL=(head and neck cancer)) AND ALL=(dental implants)) AND ALL=(radiotherapy)) AND ALL=(head and neck cancer) |
| Google Scholar | dental implants OR dental implant OR dental implantation OR implant-supported dental prosthesis OR endosseous dental implantation OR dental implant survival OR surgical dental prostheses OR surgical dental prosthesis OR tooth implant AND head and neck cancer OR head and neck tumors OR head and neck neoplasms OR head and neck Oncology OR mouth neoplasms OR oral cancer AND radiation therapy OR radiation therapies OR radiotherapy OR radiotherapies OR irradiation OR irradiation patients |

## S 2 – Primary studies included in systematic reviews

| Study | Study design | Number of implants placed | Number of implants failed | Fallow-up, months | Implant Survival rate |
|---|---|---|---|---|---|

| | | | | | |
|---|---|---|---|---|---|
| Pieralli et al. (2021) | RS | Overall 322<br>Mx 128<br>Md 194<br>HCN 217<br>RT 113<br>NRT 104<br>GB 47<br>NB 170 | NM | 81.2 ± 50.3 | Control group 100%<br>HCN 98.2%<br>RT 98.2%<br>NRT 98.1%<br>GB 97.9%<br>Irrad. GB 95.2%<br>N-irrad.NB 100%<br>NB 98.2%<br>Irrad NB 98.9%<br>N-irrad NB 97.4% |
| Neckel et al. (2020) | PS | Irrad NB 81<br>Mx 26<br>Md 55 | NM | 36 | Overvall 97.5 |
| Ettl et al. (2020) -results of previus publication Ettl et al. (2016) | PS | Overall 234<br>RT 177<br>NRT 57<br>Mx 92<br>Md 142<br>GB 42<br>NB 192 | NM | 24 | Overall<br>2 years 92.3%<br>1 year 94.4%<br>Implant success overall<br>2 years 78.6%<br>1 year 86.3%<br>Mx 73.9%<br>Md 81.7%<br>NB 81.2%<br>GB 69.1%<br>RT 76.7%<br>Inside PTV 76.2%<br>Outside PTV 77%<br>NRT 86% |
| Patel et al. (2020) | RS | Overall 376<br>RT 132<br>NRT 244<br>Mx 99<br>Md 277<br>GB 43<br>NB 333<br>14 Zygoma implantS | NM | 46,92 (1,32 – 152,12) | Without Zigoma implants<br>Overal 97.5%<br>NB 98.1%<br>Irrad NB 97.3%<br>N-irrad NB 98.6%<br>GB 93%<br>Irrad GB 88.9%<br>N-irrad GB 94.1%<br>RT 96.7%<br>NRT 97.9% |
| Albrega et al. (2020) | PS | 58<br>RT 42<br>NRT 16 | NM | Median 18,5 | Overall: 93.1%<br>RT 90.5%<br>NRT 100% |
| Sandoval et al. (2020) | RS | 29<br>All implants placed in irradiated fibula free flaps (postop RT) | NM | From surgery 7 (3–14)<br>From RT completion 3.5 (0–11) | 93.1% |
| Di Carlo et al. (2019) | RS | 84<br>Mx 36<br>Md 48<br>All in irradiated residual bone | NM | After implantation 22.9 (SD 15.5)<br>After RT 39.5 (SD 22.8) | Overall: 90.5%<br>Mx 94.4%<br>Ant 92.3%<br>Post 95.7%<br>Md 87.5%<br>Ant 100%<br>Post 82.4% |

| | | | | | |
|---|---|---|---|---|---|
| Woods et al. (2019) | RS | 102<br>RT 51<br>NRT 51<br>GB 10<br>NB 92<br>Mx and md<br>Immediate 39<br>Delayed 63 | NM | 23 (range 2–140) | Overall 93.1%<br>RT 90.2%<br>NRT 96.1%<br>GB 100%<br>NB 92.4% |
| Laverty et al. (2019) | RS | 779<br>Mx 373<br>Md 406<br>GB 112<br>NB 650<br>NB+ALT e radial 667<br>RT 382<br>RCT: 143<br>RT/RCT 525<br>NRT 254<br>Placement primary 26<br>Delayed 753 | NM | Mean 43 (range 1–142)<br>Median 38 | Overall 95.6%<br>RT/RCT 95%<br>RT 96.1%<br>RCT 92.3%<br>NRT 96.9%<br>NB 98.2%<br>NB+ALT and radial flap 97.8%<br>GB 83%<br>scapula 100%<br>fíbula 83.1%<br>DCIA 76.0%<br>Iliac crest (non-vascular) 80% |
| Moore et al. (2019) | RS | Dental implant 78<br>GB 38<br>NB 40<br>RT 63<br>NRT 15 | NM | 25.7 (6–89) | Dental implant<br>Overall 85.9%<br>NB 87.5%<br>GB 84.2%<br>RT 82.5%<br>NRT 100% |
| Papi et al. (2019) | PS | 113<br>Mx 35<br>Md 78<br>Irradiated residual bone | NM | Range 24–30 after prosthetic rehabilitation | Overall 94.7%<br>Mx 91.4%<br>Md 96.2% |
| Curi et al. (2018) | RS | 169<br>Mx 79<br>Md 90<br>All in irradiated bone | NM | 89,6 (3.6 – 176.4) | Overall: 92.9%<br>Mx 91.1%<br>Md 94.4%<br>5 years success rate<br>Mx 92.4%<br>Md 90.9% |
| Flores-Ruiz et al. (2018) | RS | 106<br>Mx 43<br>Md 63<br>GB 15 (Mx 7, Md 8)<br>NB 91 (36 Mx, Md 55)<br>RT 78<br>NRT 28 | NM<br>13 | 60 | Overall 87.7%<br>Mx 79.1%<br>Md 93.7%<br>GB 73.3%<br>Irrad GB 71.4%<br>N-irrad GB 75%<br>NB 90.1%<br>Irrad NB 87.3%<br>N-irrad NB 100%<br>RT/RCT 85.9%<br>NRT 92.9% |
| Menapace et al. (2018) | RS | 121 | NM | NM | NM |
| Pellagrino et al. (2018) | RS | 108 | NM | NM | NM |
| Sozzi et al. (2017) | RS | FFF 100<br>RT 26<br>NRT 74 | NM | NM | General Sucess Rate<br>97,9% (60 months)<br>72,8% (120 months) |

| | | | | | |
|---|---|---|---|---|---|
| Burgess et al. (2017) | RS | 199<br>FF 96<br>DCIA 64<br>Scapula 37<br>Radius 2<br>RT 45<br>NRT 154<br>All in GB | NM | 24 (6-60) from surgery | Overall 94.5%<br>RT 84.4%<br>NRT 97.4%<br>FF 91.7%<br>DCIA 96.9%<br>Scapula 97.3%<br>Radius 100% |
| Woods et al. (2017) | RS | 156 | NM | NM | NM |
| Wu et al. (2016) | RS | 198 | NM | NM | 93.6% after 10 years |
| Ettl et al. (2016) | PS | 309<br>Implants irrad bone | NM | 12 | 95.2% (irrad bone) |
| Rana et al. (2016) | RS | 162<br>Mx 70 (ant 35, post 35)<br>Md 92 (ant 52, post 40) Irrad bone | NM | 60 | Overall 67.9%<br>Mx 71.4%<br>Ant 65.7%<br>Post 77.1%<br>Md 65.2%<br>Ant 65.4%<br>Post 65% |
| Ernest et al. (2016) | RS | 194<br>Mx 73<br>Md 121<br>GB 12<br>NB 182<br>RT 88<br>NRT 106 | NM | 52,92 (24 – 117) | Overall 97.9%<br>Mx:100%<br>Md 96.7%<br>GB (n-irrad) 100%<br>NB 97.8%<br>Irrad NB 96.6%<br>N-irrad NB 98.9%<br>RT: 96.6%<br>NRT: 99.1% |
| Barber et al. (2016) | RS | 82<br>FFF 35 (RT 12, NRT 23)<br>BIFFF 47 (RT 13, NRT 34)<br>RT 25<br>NRT 57<br>All GB | NM | 60 | Overall 87.8%<br>RT 84%<br>NRT 89.5%<br>FFF 77.1%<br>RT FFF 83.3%<br>NRT FFF 73.9%<br>BIFFF 95.7%<br>RT BIFFF 84.6%<br>NRT BIFFF 100%<br>>10 packyears smokers 93.1% |
| Ch’ng et al. (2016) | RS | 1132<br>NB 889<br>Mx 271<br>Md 618<br>GB (FFF) 243<br>RT 795<br>preop 100<br>postop 695<br>NRT 337 | NM | Median 33,7 (0,9 – 92,7) | Overall 96.3%<br>3 years 92.8%, 5 years 92.2%)<br>Mx 97.8%<br>Md 97.4%<br>GB (FFF) 91.8%<br>Irrad GB 83.3%<br>Non-irrad GB 94.9%<br>NB 97.5%<br>Irrad NB 97.4%<br>N-irrad NB 98.1%<br>RT 96.2%<br>preop RT 92%<br>postop RT 96.8%<br>NRT 96.4% |

| | | | | | |
|---|---|---|---|---|---|
| Jackson et al. (2016) | RS | 15 | NM | NM | NM |
| Kobayashi et al. (2016) | RS | 134 | NM | NM | NM |
| Wetzels et al. (2016) | PS | NR | NM | 60 | NM |
| Wetzels et al. (2017) | RS | NR | NM | 60 | NM |
| Pompa et al. (2015) – patient data matches partly with Di Carlo et al. (2019) | RS | 168<br>Mx 72 (26 ant, 46 post)<br>Md 96 (28 ant, 68 post)<br>RT 51<br>NRT 117 | NM | After RT<br>39.5 SD 22.8<br>After implant placement<br>22.9 SD 15.5 | Overall 90.5%<br>Mx 94.4%<br>Md 87.5%<br>RCT 76.5%<br>NRT 96.6%<br>Mx (ant 92.3%, post 95.7%)<br>Md (ant 100%, post 82.4%) |
| Hessling et al. (2015) | RS | 272<br>Mx 83<br>Md 189<br>NB 179<br>GB 93<br>RCT 223<br>(neoadjuvant 95, adjuvante 128)<br>NRT 49 | NM | 30.9 (3 – 82) | Overall<br>2 years 98.9%,<br>5 years 97.1%<br>During observation time (≤82 meses) 96.3%<br>After 5 years<br>GB 94,6%<br>NB 98.3%<br>RCT 96.4%<br>Neoadjuvant 97.9%<br>Adjuvant 95.3%<br>NRT 100% |
| Nack et al. (2015) – 5 years follow-up of a previus publication Heberer et al. (2011) | RS | 97 (SLA 48, SLActive 49)<br>(102 at beginning, 50 SLA, 52 SLActive)<br>Mx 47<br>Md 35<br>Irrad bone<br>(Mx 55; Md 47 at beginning) | NM | 60 (36 – 72) | Overall (after 5 years) 79.4%<br>SLA 79.2%<br>SLActive 79.6% |
| Doll et al. (2015) – based partly on data published by Nelson et al. (2007) | RS | 830<br>Mx 450<br>Md 380<br>RT 292 (Mx 74, Md 118)<br>NRT 538 | NM | 121 (37 – 240) | Overall 92.2%<br>Mx 92%<br>Md 92.4%<br>NRT 93.5%<br>RT 89.7% |
| Hakim et al. (2015) | RS | 119<br>Mx and Md<br>All in GB (FFF)<br>RT 48<br>NRT 71 | NM | 94,5 ± 37,3 (3 – 172) | Overall 92.4%<br>RT 89.6%<br>NRT 94.4% |
| Saracoglu et al. (2015) | PS | Irrad 40<br>N-irrad 40<br>Mx 40<br>Md 40 | NM | 12 | Mx $p<0.002$<br>Md $p<0.001$ |

| | | | | | |
|---|---|---|---|---|---|
| Jacobsen et al. (2014) | RS | 140<br>RT 47<br>NRT 93<br>GB 99 (Irrad 13, N-irrad 86)<br>NB 41 (Irrad 34, N-irrad 7) | RT 14<br>NRT 13<br>GB 13<br>NB 6<br>12 N-irrad GB<br>1 N-irrad NB | 67 | Overall 80.7%<br>1 year 93.6%<br>5 years 83.3%<br>RT 70.2%<br>NRT 86%<br>GB 79.8%<br>Irrad GB 38.5%<br>N-irrad GB 86%<br>NB 85.4%<br>Irra NB 82.4%<br>N-irrad NB 85.7% |
| Korfage et al. (2014) | PS | 524<br>RT 318<br>NRT 206<br>Native md | NM | 45.6 (0 – 174) | Overall 93.1%<br>RT 90.3%<br>NRT 97.6% |
| Gander et al. (2014) | RS | 136<br>GB 48<br>NB 88<br>RT 84<br>NRT 52<br>All in md | NM | 20 | Overall<br>After 20 months 87.5%<br>After 12 months 92.7%<br>GB 87.5%<br>NB 87.5%<br>RT 85.7%<br>NRT 90.4% |
| Dholam et al. (2013) | PS | 85<br>GB 40<br>NB 45<br>RT 59<br>NRT 26 | NM | 60 | Survival rate NM<br>Rate of osseointegration<br>Overall 88%<br>GB 93%<br>NB 85%<br>RT 83%<br>NRT 100%<br>Success rate<br>Overall 76.5%<br>GB 72.5%<br>NB 80%<br>RT 71.2%<br>NRT 88.5% |
| Buurman et al. (2013) | RS | 73<br>Md irrad | NM | 48.6 (14 – 132) | 97.3%<br>Success rate 95.9% |
| Fierz et al. (2013) | RS | 104<br>Mx 28<br>Md 76<br>GB 46<br>NB 58<br>RT 62<br>NRT 42 | NM | 36 – 72 | Overall 82.7%<br>GB 82.6%<br>Irrad GB 70%<br>N-irrad GB 92.3%<br>NB 82.8%<br>Irrad NB 81%<br>N-irrad NB 87.5%<br>RT 77.4%<br>NRT 90.5% |
| Katsoulis et al. (2013) | RS | 104<br>RT 64<br>NRT 42<br>Mx 24<br>Md 80<br>Irrad GB 20<br>Irrad NB 42<br>N-irrad GB 26<br>N-irrad NB 16 | 18<br>RT 14<br>NRT 4<br>Mx 4<br>Md 10<br>Irrad GB 6<br>Irrad NB 8<br>N-irrad GB 2<br>N-irrad NB 2 | 24 - 60 | Overall 86.53%<br>RT 77.42%<br>NRT 90.48%<br>Mx 83.33%<br>Md 87.5%<br>Irrad GB 70%<br>Irrad NB 80.95%<br>N-irrad NB 92.31%<br>N-irrad NB 87.5% |
| Mizbah et al. (2013) | RS | NM | NM | 60 | NM |

| | | | | | |
|---|---|---|---|---|---|
| Buddula et al. (2012) | RS | 271 | NM | 60 | Overall 89.9%<br>Mx 80.5%<br>Md 93.6%<br>NB 93.4%<br>GB 83.3% |
| Mancha de La Plata et al. (2012) | RS | 355<br>RT 225<br>NRT 130<br>Irrad Mx 94<br>Irrad Md 131 | 28<br>RT 22<br>NRT 6<br>Irrad Mx 7<br>Irrad Md 15 | 45 (6 – 96) | *Overall 91.64<br>RT 90.22%<br>NRT 95.38%<br>Irrad Mx 92.55%<br>Irrad Md 88.55% |
| Linsen et al. (2012) | RS | 262<br>RT 127<br>NRT 135 | 14<br>RT 8<br>NRT 6 | 42 | Overall 86.9% (10 years)<br>RT 95.6% (10 years)<br>RT and chemotherapy 91.5% (5 years)<br>NRT 84.7% (10 years) |
| Fenlon et al. (2012) | **RS/TS | 145<br>RT 35<br>NRT 110 | 18<br>RT 15<br>NRT 3 | 36 | (1) Overall 87.58%<br>RT 57%<br>NRT 97%<br>Immediate placement 35%<br>Non-immediate 96% |
| Al-Nawas et al. (2012) | RS | 516<br>RT 87<br>NRT 429 | RT 2<br>NRT 7 | 120 | NM |
| Buddula et al. (2011) | RS | 271<br>GB 118<br>NB 212 | NM | 23 (5 – 203)<br>NB/GB 36 | Turned implants Mx 72.6% (5 years)<br>Turned implant Md 91.7% (5 years)<br>Roughened implants Mx 87.5% (5 years)<br>Roughened implants Md 100% (5 years)<br>GB Mx 82.3%<br>GB Md 98.1%,<br>NB Mx 79.8%<br>NB Md 100% |
| Buddula et al. (2011) | RS | Irrad Mx 62<br>Irrad Md 209<br>Irrad GB 59<br>Irrad NB 212 | Irrad Mx 20<br>Irrad Md 13<br>Irrad GB 8<br>Irrad NB 25 | 144 | Irrad Mx 67.64%<br>Irrad Md 93.78%<br>Irrad GB 86.14%<br>Irrad NB 88.21% |
| Bodard et al. (2011) | RS | Irrad GB 75 | Irrad GB 6 | 27,5 | Irrad. GB 80% |
| Sammartino et al. (2011) | **CCT/OS | 188<br>Irrad Mx 42<br>Irrad Md 130<br><12 127<br>≥12 61 | NR<br>Irrad Mx 18<br>Irrad Md 2<br><12 12<br>≥12 8 | 36 | Overall 89.4%<br>Irrad Mx 57.1%<br>Irrad Md 98.4%<br>>50 Gy 78.6%<br>< 50 Gy 93.6%<br><12 months 91%<br>≥12 months 87% |
| Heberer et al. (2011) – final results reported by Nack et al. (2015) | **PS/RCT /CCT | 102<br>SLA 50<br>SLActive 52<br>Irrad Mx 55<br>Irrad Md 42 | Irrad Mx 0<br>Irrad Md 2 | 14.4 (12 – 26) | SLA 96%<br>SLActive 100%<br>Irrad Mx 100%<br>Irrad Md 95.24% |
| Salinas et al. (2011) | RS | 206<br>RT 90<br>NRT 116 | 31<br>RT 23<br>NRT 8 | 41.1 (4 – 108) | Overall 85%<br>RT 74.4%<br>NRT 93.1% |

| | | | | | |
|---|---|---|---|---|---|
| | | | | | Irrad GB 72.5%<br>N-irrad GB 90.4%<br>Irrad. NB 76.9%<br>N-irrad NB 96.2% |
| Barrowman et al. (2011) | RS | *115<br>RT 48<br>NRT 67<br>Mx 35<br>Md 80<br>N-irrad Mx NB 9 | *5<br>RT 5<br>NRT 0<br>Mx 0<br>Md 5<br>N-irrad NB<br>Mx 0 | 192 | *95.65% Overall<br>89.58% RT<br>100% NRT<br>100% Mx<br>93.75% Md |
| Korfage et al. (2010) | **CCT/PS | 195<br>RT 123<br>NRT 72 | 14<br>RT 13<br>NRT 1 | 60 | *Overall 92.82%<br>RT 89.4%<br>NRT 98.6% |
| Klein et al. (2009) | RS | 190<br>RT 116<br>NRT 74<br><50 Gy 55<br>≥50 Gy 61 | 25<br>RT 13<br>NRT 12<br><50 Gy 4<br>≥50 Gy 9 | 60 | Overall 86.8%<br>RT 88.8%<br>≥50 Gy 85.2%<br><50 Gy 92.7%<br>NRT 83.8%<br>Irrad GB 89.2%<br>N-irrad GB 68.6%<br>Irrad NB 97.4%<br>N-irrad NB 91.3%<br><50 Gy 92.73%<br>≥50 Gy 85.25% |
| Cuesta-Gil et al. (2009) | RS | 706<br>RT 395<br>NRT 311 | 81<br>RT 75<br>NRT 6 | 72 - 108 | 92.9%<br>RT 81.01%<br>NRT 98.07% |
| Schoen et al. (2008) | PS | 186<br>RT 124<br>NRT 62 | NM | 18 - 24 | RT NB 97%<br>NRT NB 97% |
| Alsaadi et al. (2008) | RS | RT 15<br>NRT 1499 | RT 3<br>NRT 98 | | RT 80%<br>NRT 93.46% |
| Nelson et al. (2007) | RS | 435<br>RT 124<br>NRT 311 | 11<br>RT 7<br>NRT 4 | 120 (5 – 161) | *Overall 97.47%<br>RT 94.35%<br>NRT 98.71%<br>Overall 70% (8 years)<br>Overall 69% (13 years)<br>RT 84% (3.8 years)<br>RT 54% (13.5 years) |
| Schoen et al. (2007) | **CCT/RCT | 103<br>HBO 54<br>N-HBO 49 | 11<br>HBO 8<br>N-HBO 3 | 12 | *Overall 89.32%<br>HBO 85.19%<br>N-HBO 93.88% |
| Yerit et al. (2006) | RS | 316<br>RT 154<br>NRT 162<br>Irrad NB 154<br>N- irrad GB 78<br>N-irrad NB 84<br><12 months 143<br>>12 months 173 | 44<br>RT 29<br>NRT 15<br>Irrad NB 29<br>N- irrad GB 13<br>N-irrad NB 2<br><12 months 29<br>>12 months 15 | 60 (4 – 151) | RT 81.17%<br>NRT 90.70%<br>Irrad NB 81.17%<br>N- irrad GB 83.33%<br>N-irrad NB 97.62%<br><12 months 79.72%<br>>12 months 91.33% |
| Schepers et al. (2006) | RS | 139<br>RT 61<br>NRT 78 | 2<br>RT 2<br>NRT 0 | 96 | *Overall 98.56%<br>RT 97%<br>NRT 100% |

| | | | | | |
|---|---|---|---|---|---|
| Landés and Kovács (2006) | PS | 114<br>72 RT 72<br>42 NRT 4 | 1<br>1 RT 1<br>0 NRT 0 | Mean 36<br>(24 – 46) | *Overall 99,12%<br>98.61% RT<br>100% NRT |
| Bodard et al. (2006) | RS | 68<br>6 Irrad Mx 6<br>62 Irrad Md 62 | 0 Irrad Mx 0<br>0 Irrad Md 0 | Mean 31.9 | *Overall 100%<br>100% Irrad Mx<br>100% Irrad Md |
| Chispasco et al. (2006) | RS | 71 | NM | NM | NM |
| Teoh et al. (2005) | RS | 102<br>RT 30<br>NRT 72<br>HBO 15<br>N-HBO 15 | 6<br>RT 5<br>NRT 1<br>HBO 5<br>N-HBO 0 | Mean 51.7<br>(1.3 – 138) | *Overall 94.11%<br>RT 83.33%<br>NRT 98.61%<br>HBO 66.67%<br>N-HBO 100% |
| Shaw et al. (2005) | RS | 364<br>RT 172<br>NRT 192<br>GB 123<br>NB 241<br>HBO 77<br>N-HBO 95<br>Irrad Md 98<br>N-irrad Md 110<br><50Gy 44<br>=50Gy 78<br>>50Gy 33<br>Mx 42<br>Md 199 | 56<br>RT 31<br>NRT 25<br>GB 32<br>NB 24<br>HBO 15<br>N-HBO 17<br>Irrad Md 1<br>N-irrad Md 15<br><50 Gy 11<br>=50 Gy 12<br>>50 Gy 8<br>Mx 8<br>Md 16 | Mean 3.5 years (0.3 – 14) | *Overall 84.61%<br>RT 81.98%<br>NRT 86.98%<br>GB 73.98%<br>NB 90.04%<br>HBO 85.71%<br>N-HBO 82.11%<br>Md 98.88%<br>N-irrad Md 86.36%<br><50 Gy 75%<br>=50 Gy 84.62%<br>>50 Gy 75.76%<br>Mx 80.95%<br>Md 91.96% |
| Granström (2005) | RS | 1245<br>RT 631<br>RT 614<br>HBO 340<br>N-HBO 291 | 223<br>RT 147<br>NRT 76<br>HBO 29<br>N-HBO 117 | 72 (6 – 276) | *Overall 82.08%<br>RT 75%<br>NRT 87%<br>HBO 91.47%<br>N-HBO 59.79% |
| Granström (2003) | RS | 206<br>HBO 133<br>N-HBO 73<br>Irrad Mx 109<br>Irrad Md 97<br>Irrad GB 40<br>Irrad NB 14 | 22<br>HBO 5<br>N-HBO 17<br>Irrad Mx 14<br>Irrad Md 8<br>Irrad GB 0<br>Irrad NB 2 | Mean 9.8 years (2.2 – 22) | *Geral 89.32%<br>HBO 96.2%<br>N-HBO 76.7%<br>Irrad Mx 87.2%<br>Irrad Md 91.8%<br>Irrad GB 100%<br>Irrad NB 100% |
| Cao et al. (2003) | RS | 131<br>RT 53<br>NRT 78 | 29<br>RT 18<br>NRT 11 | 60 | Overall 65%<br>RT 49.44%<br>NRT 77.8% |
| Schoen et al. (2003) | PS | NM | NM | 13 - 40 | NM |
| Visch et al. (2002) | PS | 446<br>Irrad Mx 108<br>Irrad Md 338<br><50 Gy 207<br>≥50 Gy 239<br><12 months 175<br>≥12 months 271 | 64<br>Irrad Mx 33<br>Irrad Md 31<br><50 Gy 19<br>≥50 Gy 45<br><12 months 29<br>≥12 months 35 | 120 | Overall 78%<br>Irrad Mx 88.04%<br>Irrad Md 95.45%<br><50 Gy 90,82%<br>≥50 Gy 81.18%<br><12 months 94.12%<br>≥12 months 90.18% |
| Van Steen-berghe et al. (2002) | CCT | 1266<br>RT 33<br>NRT 1230 | 27<br>RT 2<br>NRT 25 | Mean 1881 days (72 – 3901) | *Overall 97,86%<br>RT 93.94%<br>NRT 97.97% |

| | | | | | |
|---|---|---|---|---|---|
| Goto et al. (2002) | RS | 180<br>RT 92<br>NRT 88<br>Mx 52<br>Md 128<br>Irrad GB 68<br>Irrad NB 112 | 15<br>RT 11<br>NRT 4<br>Mx 11<br>Md 4<br>Irrad GB 4<br>Irrad NB 11 | 2 - 130 | *Overall 91.66%<br>RT 88.04%<br>NRT 95.45%<br>Mx 78.85%<br>Md 96.87%<br>Irrad GB 94.14%<br>Irrad NB 90.18% |
| Grötz et al. (1999) | RS | 197 | NM | 72 | 72% |
| Betz et al. (1999) | PS | 261 | NM | NM | 77,8% |
| Weischer and Mohr (1999) | RS | 175<br>RT 83<br>NRT 92 | 15<br>10 (GRT)<br>5 (GNRT) | 37 | Overall 91% (3 years)<br>GRT 87.95%<br>GNRT 94.57%<br>RT 75% (7 years)<br>NRT 86% (10 years) |
| Schliephake et al. (1999) | RS | 409<br>RT 145<br>NRT 264 | NM | 120 | Overall 56,6%<br>RT 49.8%<br>NRT 57.7% |
| Werkmeister et al. (1999) | RS | 109<br>RT 30<br>NRT 79<br>N-irrad GB 45<br>Irrad NB 30<br>N-irrad NB 34<br>>54 Gy 16<br><54 Gy 14 | 27<br>RT 8<br>NRT 19<br>N-irrad GB 14<br>Irrad NB 8<br>N-irrad NB 5<br>>54 Gy5<br><54 Gy 3 | 36 | *Overall 75.22%<br>RT 73%<br>NRT 75.94%<br>N-irrad GB 68.89%<br>Irrad NB 73.33%<br>N-irrad NB 85.29%<br>>54 Gy) 68.75%<br><54 Gy 78.57% |
| Mericske-Stem et al. (1999) | CCT | 53<br>RT 33<br>NRT 20<br>Mx 12<br>Md 41 | 8<br>RT 8<br>NRT 0<br>Mx 0<br>Md 8 | 37 (12 – 84) | *Overall 84.90%<br>RT 7.76%<br>NRT 100%<br>Mx 100%<br>Md 80.49% |
| Keller et al. (1999) | RS | 248<br>RT 11<br>NRT 237 | 33<br>RT 0<br>NRT 33 | 120 | *Overall 86.69%<br>RT 100%<br>NRT 86.08 |
| Granström et al. (1999) | RS | 335<br>RT 246<br>NRT 89<br>HBO 99<br>N-HBO 147 | 99<br>RT 87<br>NRT 12<br>HBO 8<br>N-HBO 79 | 44.2 – 88.8 | *Overall 70.44%<br>RT64.63%<br>NRT 86.52%<br>HBO 91.92%<br>N-HBO 46.26% |
| Foster et al. (1999) | RS | 104<br>Irrad GB 15<br>N-irrad GB 89 | 7<br>Irrad GB 0<br>N-irrad GB 7 | 132 | *Overall 93.26<br>Irrad GB100%<br>N-irrad GB 92.13% |
| Esser et al. (1999) | RS | 276<br>RT 148<br>NRT 128 | 12<br>RT 9<br>NRT 3 | 58.2 | *Overall 95.65%<br>RT 93.92%<br>NRT 97.66% |
| Niimi et al. (1998) | RS | RT 228<br>Mx 59<br>Md 169 | RT 20<br>Mx 17<br>Md 3 | 49 | *Overall 91.22<br>Japan 88.9%<br>USA 86% |
| Ihara et al. (1998) | RS | 74<br>RT39<br>NRT 35<br>HBO 19<br>N-HBO 20 | 15<br>RT 6<br>NRT 9<br>HBO 3<br>N-HBO 3 | 27.6 | *Overall 79.711%<br>RT 84.62%<br>NRT 74.29%<br>HBO 84.21%<br>N-HBO 85% |

| | | | | | |
|---|---|---|---|---|---|
| Brogniez et al. (1998) | RS | 53<br>Irrad Mx 3<br>Irrad Md 50 | 2<br>Irrad Mx 0<br>Irrad Md 2 | 38 (6 – 68) | *Overall 96.22%<br>Irrad Mx 100%<br>Irrad Md 96% |
| Andersson et al. (1998) | RS | 90<br>Irrad Mx 12<br>Irrad Md 78 | 2<br>Irrad Mx 0<br>Irrad Md 2 | 96 | *Overall 97.77%<br>Irrad Mx 100%<br>Irrad Md 97.44% |
| August et al. (1998) | RS | 40<br>RT 38<br>NRT 2 | 0 | 16.4 | 100% |
| Wagner et al. (1998) | RS | 275<br>RT 145<br>NRT 130 | NM | 65 | Overall 97.9% (5 years)<br>Overall 72.8% (10 years) |
| Niimi et al. (1997) | RS | 110<br>Irrad Mx 39<br>Irrad Md 71<br>HBO 31<br>N-HBO 79<br>≤45 Gy 67<br>>45 Gy 51 | 12<br>Irrad Mx 9<br>Irrad Md 3<br>HBO 4<br>N-HBO 8<br>≤45 Gy 7<br>>45 Gy 5 | 2 72 | *Overall 89.09%<br>Irrad Mx 76.92%<br>Irrad Md 95.77%<br>HBO 87.1%<br>N-HBO 89.87%<br>≤45 Gy 89.55%<br>>45 Gy 90.2% |
| Keller et al. (1997) | RS | 98<br>Irrad GB 26<br>Irrad NB 72 | 1<br>Irrad GB 1<br>Irrad NB 0 | 120 | *Overall 98.97<br>Irrad GB 96.15%<br>Irrad NB 100% |
| Marker et al. (1997) | RS | RT 19<br>NRT 19 | RT 0<br>NRT 0 | Mean 14<br>(7 – 47) | RT 100%<br>NRT 100% |
| Chan et al. (1997) | RS | 69<br>RT 23<br>NRT 46 | 4<br>RT 4<br>NRT 0 | Mean 32<br>(6 – 84) | *Overall 94.20<br>RT 82.61%<br>NRT 100% |
| Ali et al. (1997) | RS | 42<br>Irrad Mx 10<br>Irrad Md 32 | 6<br>Irrad Mx 6<br>Irrad Md 0 | Mean 33<br>(11 – 64) | *Overall 85.71%<br>Irrad Mx 40<br>Irrad Md 100% |
| Esser and Wagner (1997) | RS | 320<br>RT 249<br>NRT 71 | 46<br>RT 39<br>NRT 7 | 60 | *Overall 85.62%<br>RT 84.34%<br>NRT 90.14% |
| Jisander et al. (1997) | **CCT/RS | 103<br>Irrad Mx 38<br>Irrad Md 65 | 5<br>Irrad Mx 3<br>Irrad Md 2 | 21 (1 – 62) | *Overall 95.14%<br>Mx 92% (1 year)<br>Mn 97% (1 year) |
| Roumanas et al. (1997) | RS | 80<br>RT 45<br>NRT 35<br>Irrad GB 39<br>N-irrad GB 32<br>Irrad NB 5<br>N-irrad NB 4 | 1<br>RT 0<br>NRT 1<br>Irrad GB 0<br>N-irrad GB 1<br>Irrad NB 0<br>N-irrad NB 0 | 1 – 49 | *Overall 98.75%<br>RT 100%<br>NRT 90.1%<br>Irrad GB 100%<br>N-irrad GB 96.9%<br>Irrad NB 100%<br>N-irrad NB 100% |
| McGhee et al. (1997) | RS | 26<br>RT 21<br>NRT 5<br>Irrad GB 9<br>Irrad NB 12<br>N-irrad GB 5 | 2<br>RT 3<br>NRT 0<br>Irrad GB 0<br>Irrad NB 2<br>N-irrad GB 0 | 3 years | *Overall 92.30%<br>RT 90.48%<br>NRT 100%<br>Irrad GB 100%<br>Irrad NB 83.33%<br>N-irrad GB 100% |

| | | | | | |
|---|---|---|---|---|---|
| Watzinger et al. (1996) | RS | 138 | NM | NM | GB 58.3%<br>NB 87.8% |
| Weischer et al. (1996) | **CCT/PS | 105<br>RT 57<br>NRT 48 | 7<br>RT 4<br>NRT3 | 26 | *Overall 93.33%<br>RT 92.98%<br>NRT 93.75 |
| Eckert et al. (1996) | RS | 111<br>Irrad Mx 22<br>Irrad Md 89 | 9<br>Irrad Mx 8<br>Irrad Md 1 | 12 years | *Overall 91.89%<br>Irrad Mx 63.64%<br>Irrad Md 98.88% |
| Aldegheri et al. (1996) | RS | 19<br>Irrad Mx 6<br>Irrad Md 13 | 0<br>Irrad Mx 0<br>Irrad Md 0 | Mean 2,7 years (1 – 4) | *Overall 100%<br>Irrad Mx 100%<br>Irrad Md 100% |
| Franzén et al. (1995) | RS | 20<br><50Gy 13<br>≥50Gy 7 | 1<br><50Gy 0<br>≥50Gy 1 | 36 – 72 | *Overall 95%<br><50Gy 100%<br>≥50Gy 85.71% |
| Sclaroff et al. (1994) | RS | 114<br>RT 80<br>NRT 34 | 2<br>RT 0<br>NRT 2 | 3 years 2 months | *Overall 98,24%<br>RT 100%<br>NRT 94.1% |
| Albrektsson et al. (1988) | RS | 8045<br>RT 49<br>NRT 7996<br>Irrad Mx 16<br>Irrad Md 33<br>N-irrad Mx 3089<br>N- irrad Md 4907 | 273<br>RT 3<br>NRT 270<br>Irrad Mx 3<br>Irrad RMd 0<br>N-irrad Mx 218<br>N-irrad Md 52 | 3 – 8 years | *Overall 96.60%<br>RT 93.88 %<br>NRT 93.88 %<br>Irrad Mx 81.25%<br>Irrad Md 100%<br>N-irrad Mx 92.94%<br>N-irrad Md 98.94% |

Abbreviations: ALT: anterolateral thigh flap, ant: anterior; BIFFF: bone-impacted fibula free flap, CCT: clinical controlled trial, DCIA: deep circumflex iliac artery, FFF: fibula free flap,iado, GB: grafted bone, HBO: group hyperbaric oxygen therapy, HCN: head and neck cancer, Irrad GB: irradiated grafted bone group, Irrad NB: irradiated native bone group, Irrad Md: mandibular irradiated group, Irrad Mx: maxillary irradiated group, Md: mandible, Mx: maxila, NB: native bone, N-HBO:group without hyperbaric oxygen therapy, N-irrad GB: non-irradiated grafted bone group, N_irrad NB: non-irradiated native bone group, N-irrad Md: mandibular non-irradiated group, N-irrad Mx: maxillary non-irradiated group, NRT: non-irradiated group, post: posterior, PS:prospective study, RS: retrospective study, RCT: randomized controlled trial, RT: group of irradiated patients, SLA: sandblasted and acid etched and SLActive: modified sandblasted and acid etched.
* Data obtained by arithmetic calculation carried out by the authors.
** Different classification in systematic reviews.

## Reference


Pieralli S, Spies BC, Schweppe F, Preissner S, Nelson K, Heiland M, Nahles S. Retrospective long-term clinical evaluation of implant-prosthetic rehabilitations after head and neck cancer therapy. Clin Oral Implants Res. 2021 Apr;32(4):470-486. doi: 10.1111/clr.13716. Epub 2021 Feb 7. PMID: 33501694. https://doi.org/10.1111/clr.13716

Neckel N, Wagendorf P, Sachse C, Stromberger C, Vach K, Heiland M, Nahles S. Influence of implant-specific radiation doses on peri-implant hard and soft tissue: An observational pilot study. Clin Oral Implants Res. 2021 Feb;32(2):249-261. doi: 10.1111/clr.13696. Epub 2020 Dec 21. PMID: 33278849. https://doi.org/10.1111/clr.13696

Ettl T, Junold N, Zeman F, Hautmann M, Hahnel S, Kolbeck C, Müller S, Klingelhöffer C, Reichert TE, Meier JK. Implant survival or implant success? Evaluation of implant-based prosthetic rehabilitation in head and neck cancer patients-a prospective

observational study. Clin Oral Investig. 2020 Sep;24(9):3039-3047. doi: 10.1007/s00784-019-03172-9. Epub 2019 Dec 12. PMID: 31832793.

Patel J, Antov H, Nixon P. Implant-supported oral rehabilitation in oncology patients: a retrospective cohort study. Br J Oral Maxillofac Surg. 2020 Oct;58(8):1003-1007. doi: 10.1016/j.bjoms.2020.05.016. Epub 2020 May 27. PMID: 32474015. https://doi.org/10.1016/j.bjoms.2020.05.016

Alberga JM, Korfage A, Bonnema I, Witjes MJH, Vissink A, Raghoebar GM. Mandibular dental implant placement immediately after teeth removal in head and neck cancer patients. Support Care Cancer. 2020 Dec;28(12):5911-5918. doi: 10.1007/s00520-020-05431-y. Epub 2020 Apr 11. PMID: 32279135; PMCID: PMC7686200. https://doi.org/10.1007/s00520-020-05431-y

Sandoval ML, Rosen EB, Robert AJ, Nelson JA, Matros E, Gelblum DY. Immediate dental implants in fibula free flaps to reconstruct the mandible: A pilot study of the short-term effects on radiotherapy for patients with head and neck cancer. Clin Implant Dent Relat Res. 2020 Feb;22(1):91-95. doi: 10.1111/cid.12870. Epub 2019 Nov 28. PMID: 31777154; PMCID: PMC9348905.

Di Carlo S, De Angelis F, Ciolfi A, Quarato A, Piccoli L, Pompa G, Brauner E. Timing for implant placement in patients treated with radiotherapy of head and neck. Clin Ter. 2019 Sep-Oct;170(5):e345-e351. doi: 10.7417/CT.2019.2153. PMID: 31612191. https://doi.org/10.7417/CT.2019.2153

Woods B, Schenberg M, Chandu A. A Comparison of Immediate and Delayed Dental Implant Placement in Head and Neck Surgery Patients. J Oral Maxillofac Surg. 2019 Jun;77(6):1156-1164. doi: 10.1016/j.joms.2019.02.007. Epub 2019 Feb 13. PMID: 30851250.

Laverty DP, Addison O, Wubie BA, Heo G, Parmar S, Martin T, Praveen P, Pearson D, Newsum D, Murphy M, Bateman G. Outcomes of implant-based oral rehabilitation in head and neck oncology patients-a retrospective evaluation of a large, single regional service cohort. Int J Implant Dent. 2019 Mar 5;5(1):8. doi: 10.1186/s40729-019-0161-y. PMID: 30834461; PMCID: PMC6399356.

Moore P, Grinsell D, Lyons B, Hewson I. Outcomes of dental and craniofacial osseointegrated implantation in head and neck cancer patients. Head Neck. 2019 Sep;41(9):3290-3298. doi: 10.1002/hed.25845. Epub 2019 Jun 19. PMID: 31215724.

Papi P, Brauner E, Di Carlo S, Musio D, Tombolini M, De Angelis F, Valentini V, Tombolini V, Polimeni A, Pompa G. Crestal bone loss around dental implants placed in head and neck cancer patients treated with different radiotherapy techniques: a prospective cohort study. Int J Oral Maxillofac Surg. 2019 May;48(5):691-696. doi: 10.1016/j.ijom.2018.10.021. Epub 2018 Nov 22. PMID: 30471831. https://doi.org/10.1016/j.ijom.2018.10.021

Curi MM, Condezo AFB, Ribeiro KDCB, Cardoso CL. Long-term success of dental implants in patients with head and neck cancer after radiation therapy. Int J Oral Maxillofac Surg. 2018 Jun;47(6):783-788. doi: 10.1016/j.ijom.2018.01.012. Epub 2018 Feb 6. PMID: 29426738.

Flores-Ruiz R, Castellanos-Cosano L, Serrera-Figallo MA, Cano-Díaz E, Torres-Lagares D, Gutiérrez-Pérez JL. Implant survival in patients with oral cancer: A 5-year follow-up.

J Clin Exp Dent. 2018 Jun 1;10(6):e603-e609. doi: 10.4317/jced.54937. PMID: 29930780; PMCID: PMC6005093.

Menapace DC, Van Abel KM, Jackson RS, Moore EJ. Primary vs Secondary Endosseous Implantation After Fibular Free Tissue Reconstruction of the Mandible for Osteoradionecrosis. JAMA Facial Plast Surg. 2018 Sep 1;20(5):401-408. doi: 10.1001/jamafacial.2018.0263. PMID: 29801119; PMCID: PMC6233614.

Pellegrino G, Tarsitano A, Ferri A, Corinaldesi G, Bianchi A, Marchetti C. Long-term results of osseointegrated implant-based dental rehabilitation in oncology patients reconstructed with a fibula free flap. Clin Implant Dent Relat Res. 2018 Oct;20(5):852-859. doi: 10.1111/cid.12658. Epub 2018 Aug 24. PMID: 30144257.

Sozzi D, Novelli G, Silva R, Connelly ST, Tartaglia GM. Implant rehabilitation in fibula-free flap reconstruction: A retrospective study of cases at 1-18 years following surgery. J Craniomaxillofac Surg. 2017 Oct;45(10):1655-1661. doi: 10.1016/j.jcms.2017.06.021. Epub 2017 Jul 8. PMID: 28823690. https:// doi. org/ 10. 1016/j. jcms. 2017. 06. 021

Burgess M, Leung M, Chellapah A, Clark JR, Batstone MD. Osseointegrated implants into a variety of composite free flaps: A comparative analysis. Head Neck. 2017 Mar;39(3):443-447. doi: 10.1002/hed.24609. Epub 2016 Nov 23. PMID: 27880030.

Woods BJ, Chandu A. Retrospective Study of Survival of Extraoral Endosseous Craniofacial Implants in Head and Neck Surgery Patients. Int J Oral Maxillofac Implants. 2017 Nov/Dec;32(6):1405-1411. doi: 10.11607/jomi.4734. PMID: 29140386.

Wetzels JGH, Meijer GJ, Koole R, Adang EM, Merkx MAW, Speksnijder CM. Costs and clinical outcomes of implant placement during ablative surgery and postponed implant placement in curative oral oncology: a five-year retrospective cohort study. Clin Oral Implants Res. 2017 Nov;28(11):1433-1442. doi: 10.1111/clr.13008. Epub 2017 Mar 2. PMID: 28251678.

Ettl T, Weindler J, Gosau M, Müller S, Hautmann M, Zeman F, Koller M, Papavasileiou D, Bürgers R, Driemel O, Schneider I, Klingelhöffer C, Meier J, Wahlmann U, Reichert TE. Impact of radiotherapy on implant-based prosthetic rehabilitation in patients with head and neck cancer: A prospective observational study on implant survival and quality of life-Preliminary results. J Craniomaxillofac Surg. 2016 Sep;44(9):1453-62. doi: 10.1016/j.jcms.2016.07.016. Epub 2016 Jul 21. PMID: 27519660.

Wu Y, Huang W, Zhang Z, Zhang Z, Zou D. Long-term success of dental implant-supported dentures in postirradiated patients treated for neoplasms of the maxillofacial skeleton: a retrospective study. Clin Oral Investig. 2016 Dec;20(9):2457-2465. doi: 10.1007/s00784-016-1753-z. Epub 2016 Feb 24. Erratum in: Clin Oral Investig. 2017 Jun 21;: PMID: 26907545. https:// doi. org/ 10. 1007/ s00784- 016- 1753-z

Rana MC, Solanki S, Pujari SC, Shaw E, Sharma S, Anand A, Singh HP. Assessment of the Survival of Dental Implants in Irradiated Jaws Following Treatment of Oral Cancer: A Retrospective Study. Niger J Surg. 2016 Jul-Dec;22(2):81-85. doi: 10.4103/1117-6806.182741. PMID: 27843270; PMCID: PMC5013747.

Ernst N, Sachse C, Raguse JD, Stromberger C, Nelson K, Nahles S. Changes in Peri-Implant Bone Level and Effect of Potential Influential Factors on Dental Implants in Irradiated and Nonirradiated Patients Following Multimodal Therapy Due to Head and Neck Cancer: A Retrospective Study. J Oral Maxillofac Surg. 2016 Oct;74(10):1965-73.

doi: 10.1016/j.joms.2016.06.005. Epub 2016 Jun 12. PMID: 27376183. https://doi.org/10.1016/j.joms.2016.06.005

Barber BR, Dziegelewski PT, Chuka R, O'Connell D, Harris JR, Seikaly H. Bone-impacted fibular free flap: Long-term dental implant success and complications compared to traditional fibular free tissue transfer. Head Neck. 2016 Apr;38 Suppl 1:E1783-7. doi: 10.1002/hed.24315. Epub 2015 Dec 17. PMID: 26681661.

Ch'ng S, Skoracki RJ, Selber JC, Yu P, Martin JW, Hofstede TM, Chambers MS, Liu J, Hanasono MM. Osseointegrated implant-based dental rehabilitation in head and neck reconstruction patients. Head Neck. 2016 Apr;38 Suppl 1:E321-7. doi: 10.1002/hed.23993. Epub 2015 Jun 29. PMID: 25546139.

Jackson RS, Price DL, Arce K, Moore EJ. Evaluation of Clinical Outcomes of Osseointegrated Dental Implantation of Fibula Free Flaps for Mandibular Reconstruction. JAMA Facial Plast Surg. 2016 May 1;18(3):201-6. doi: 10.1001/jamafacial.2015.2271. PMID: 26868226.

Kobayashi Y, Sumida T, Ishikawa A, Mori Y. The Contribution of Dental Implants to Functional Artificial Restoration After Treatment of Oral Cancer. Anticancer Res. 2016 Jun;36(6):3053-6. PMID: 27272826.

Wetzels JW, Koole R, Meijer GJ, de Haan AF, Merkx MA, Speksnijder CM. Functional benefits of implants placed during ablative surgery: A 5-year prospective study on the prosthodontic rehabilitation of 56 edentulous oral cancer patients. Head Neck. 2016 Apr;38 Suppl 1:E2103-11. doi: 10.1002/hed.24389. Epub 2016 Feb 13. PMID: 26873437.

Pompa G, Saccucci M, Di Carlo G, Brauner E, Valentini V, Di Carlo S, Gentile T, Guarino G, Polimeni A. Survival of dental implants in patients with oral cancer treated by surgery and radiotherapy: a retrospective study. BMC Oral Health. 2015 Jan 20;15:5. doi: 10.1186/1472-6831-15-5. PMID: 25599761; PMCID: PMC4324417.

Hessling SA, Wehrhan F, Schmitt CM, Weber M, Schlittenbauer T, Scheer M. Implant-based rehabilitation in oncology patients can be performed with high long-term success. J Oral Maxillofac Surg. 2015 May;73(5):889-96. doi: 10.1016/j.joms.2014.11.009. Epub 2014 Dec 6. PMID: 25773649.

Nack C, Raguse JD, Stricker A, Nelson K, Nahles S. Rehabilitation of irradiated patients with chemically modified and conventional SLA implants: five-year follow-up. J Oral Rehabil. 2015 Jan;42(1):57-64. doi: 10.1111/joor.12231. Epub 2014 Sep 18. PMID: 25231029.

Doll C, Nack C, Raguse JD, Stricker A, Duttenhoefer F, Nelson K, Nahles S. Survival analysis of dental implants and implant-retained prostheses in oral cancer patients up to 20 years. Clin Oral Investig. 2015 Jul;19(6):1347-52. doi: 10.1007/s00784-014-1359-2. Epub 2014 Nov 23. PMID: 25416636.

Hakim SG, Kimmerle H, Trenkle T, Sieg P, Jacobsen HC. Masticatory rehabilitation following upper and lower jaw reconstruction using vascularised free fibula flap and enossal implants-19 years of experience with a comprehensive concept. Clin Oral Investig. 2015 Mar;19(2):525-34. doi: 10.1007/s00784-014-1247-9. Epub 2014 May 7. PMID: 24802630.

Karayazgan-Saracoglu B, Atay A, Zulfikar H, Erpardo Y. Assessment of implant stability of patients with and without radiotherapy using resonance frequency analysis. J Oral

Implantol. 2015 Feb;41(1):30-5. doi: 10.1563/AAID-JOI-D-12-00107. Epub 2012 Dec 27. PMID: 23270595.

Jacobsen C, Kruse A, Lübbers HT, Zwahlen R, Studer S, Zemann W, Seifert B, Grätz KW. Is mandibular reconstruction using vascularized fibula flaps and dental implants a reasonable treatment? Clin Implant Dent Relat Res. 2014 Jun;16(3):419-28. doi: 10.1111/cid.12004. Epub 2012 Sep 21. PMID: 22998581.

Korfage A, Raghoebar GM, Slater JJ, Roodenburg JL, Witjes MJ, Vissink A, Reintsema H. Overdentures on primary mandibular implants in patients with oral cancer: a follow-up study over 14 years. Br J Oral Maxillofac Surg. 2014 Nov;52(9):798-805. doi: 10.1016/j.bjoms.2014.05.013. Epub 2014 Jun 17. Erratum in: Br J Oral Maxillofac Surg. 2015 Apr;53(4):e23-5. PMID: 24951245.

Gander T, Studer S, Studer G, Grätz KW, Bredell M. Medium-term outcome of Astra Tech implants in head and neck oncology patients. Int J Oral Maxillofac Surg. 2014 Nov;43(11):1381-5. doi: 10.1016/j.ijom.2014.05.005. Epub 2014 Jun 3. PMID: 24907130.

Dholam KP, Pusalkar HA, Yadav PS, Quazi GA, Somani PP. Implant-retained dental rehabilitation in head and neck cancer patients: an assessment of success and failure. Implant Dent. 2013 Dec;22(6):604-9. doi: 10.1097/ID.0b013e3182a4d7bc. PMID: 24149001.

Buurman DJ, Vaassen LA, Bockmann R, Kessler P. Prosthetic rehabilitation of head and neck cancer patients focusing on mandibular dentures in irradiated patients. Int J Prosthodont. 2013 Nov-Dec;26(6):557-62. doi: 10.11607/ijp.3496. PMID: 24179970.

Fierz J, Hallermann W, Mericske-Stern R. Patients with oral tumors. Part 1: Prosthetic rehabilitation following tumor resection. Schweiz Monatsschr Zahnmed. 2013;123(2):91-105. English, German. PMID: 23512240.

Katsoulis J, Fierz J, IIzuka T, Mericske-Stern R. Prosthetic rehabilitation, implant survival and quality of life 2 to 5 years after resection of oral tumors. Clin Implant Dent Relat Res. 2013 Feb;15(1):64-72. doi: 10.1111/j.1708-8208.2010.00328.x. Epub 2011 Mar 22. PMID: 21435159.

Mizbah K, Dings JP, Kaanders JH, van den Hoogen FJ, Koole R, Meijer GJ, Merkx MA. Interforaminal implant placement in oral cancer patients: during ablative surgery or delayed? A 5-year retrospective study. Int J Oral Maxillofac Surg. 2013 May;42(5):651-5. doi: 10.1016/j.ijom.2012.09.013. Epub 2012 Oct 24. PMID: 23102901.

Buddula A, Assad DA, Salinas TJ, Garces YI, Volz JE, Weaver AL. Survival of dental implants in irradiated head and neck cancer patients: a retrospective analysis. Clin Implant Dent Relat Res. 2012 Oct;14(5):716-22. doi: 10.1111/j.1708-8208.2010.00307.x. Epub 2010 Oct 26. PMID: 20977609.

Mancha de la Plata M, Gías LN, Díez PM, Muñoz-Guerra M, González-García R, Lee GY, Castrejón-Castrejón S, Rodríguez-Campo FJ. Osseointegrated implant rehabilitation of irradiated oral cancer patients. J Oral Maxillofac Surg. 2012 May;70(5):1052-63. doi: 10.1016/j.joms.2011.03.032. Epub 2011 Jul 22. PMID: 21778009.

Linsen SS, Martini M, Stark H. Long-term results of endosteal implants following radical oral cancer surgery with and without adjuvant radiation therapy. Clin Implant Dent Relat

Res. 2012 Apr;14(2):250-8. doi: 10.1111/j.1708-8208.2009.00248.x. Epub 2009 Oct 16. PMID: 19843104.

Fenlon MR, Lyons A, Farrell S, Bavisha K, Banerjee A, Palmer RM. Factors affecting survival and usefulness of implants placed in vascularized free composite grafts used in post-head and neck cancer reconstruction. Clin Implant Dent Relat Res. 2012 Apr;14(2):266-72. doi: 10.1111/j.1708-8208.2009.00250.x. Epub 2009 Oct 16. PMID: 19843102.

Al-Nawas B, Kämmerer PW, Morbach T, Ladwein C, Wegener J, Wagner W. Ten-year retrospective follow-up study of the TiOblast dental implant. Clin Implant Dent Relat Res. 2012 Mar;14(1):127-34. doi: 10.1111/j.1708-8208.2009.00237.x. Epub 2010 Feb 11. PMID: 20156231.

Buddula A, Assad DA, Salinas TJ, Garces YI, Volz JE, Weaver AL. Survival of turned and roughened dental implants in irradiated head and neck cancer patients: a retrospective analysis. J Prosthet Dent. 2011 Nov;106(5):290-6. doi: 10.1016/S0022-3913(11)60133-9. PMID: 22024178.

Buddula A, Assad DA, Salinas TJ, Garces YI. Survival of dental implants in native and grafted bone in irradiated head and neck cancer patients: a retrospective analysis. Indian J Dent Res. 2011 Sep-Oct;22(5):644-8. doi: 10.4103/0970-9290.93449. PMID: 22406706.

Bodard AG, Bémer J, Gourmet R, Lucas R, Coroller J, Salino S, Breton P. Dental implants and free fibula flap: 23 patients. Rev Stomatol Chir Maxillofac. 2011 Apr;112(2):e1-4. doi: 10.1016/j.stomax.2011.01.008. Epub 2011 Mar 2. PMID: 21371725.

Sammartino G, Marenzi G, Cioffi I, Teté S, Mortellaro C. Implant therapy in irradiated patients. J Craniofac Surg. 2011 Mar;22(2):443-5. doi: 10.1097/SCS.0b013e318207b59b. PMID: 21403560.

Heberer S, Kilic S, Hossamo J, Raguse JD, Nelson K. Rehabilitation of irradiated patients with modified and conventional sandblasted acid-etched implants: preliminary results of a split-mouth study. Clin Oral Implants Res. 2011 May;22(5):546-51. doi: 10.1111/j.1600-0501.2010.02050.x. Epub 2010 Dec 2. PMID: 21121960.

Barrowman RA, Wilson PR, Wiesenfeld D. Oral rehabilitation with dental implants after cancer treatment. Aust Dent J. 2011 Jun;56(2):160-5. doi: 10.1111/j.1834-7819.2011.01318.x. PMID: 21623807.

Salinas TJ, Desa VP, Katsnelson A, Miloro M. Clinical evaluation of implants in radiated fibula flaps. J Oral Maxillofac Surg. 2010 Mar;68(3):524-9. doi: 10.1016/j.joms.2009.09.104. PMID: 20171471.

Korfage A, Schoen PJ, Raghoebar GM, Roodenburg JL, Vissink A, Reintsema H. Benefits of dental implants installed during ablative tumour surgery in oral cancer patients: a prospective 5-year clinical trial. Clin Oral Implants Res. 2010 Sep;21(9):971-9. doi: 10.1111/j.1600-0501.2010.01930.x. PMID: 20701621.

Klein MO, Grötz KA, Walter C, Wegener J, Wagner W, Al-Nawas B. Functional rehabilitation of mandibular continuity defects using autologous bone and dental implants - prognostic value of bone origin, radiation therapy and implant dimensions. Eur Surg Res. 2009;43(3):269-75. doi: 10.1159/000229027. Epub 2009 Jul 23. PMID: 19628944.

Cuesta-Gil M, Ochandiano Caicoya S, Riba-García F, Duarte Ruiz B, Navarro Cuéllar C, Navarro Vila C. Oral rehabilitation with osseointegrated implants in oncologic patients. J Oral Maxillofac Surg. 2009 Nov;67(11):2485-96. doi: 10.1016/j.joms.2008.03.001. PMID: 19837322.

Schoen PJ, Raghoebar GM, Bouma J, Reintsema H, Burlage FR, Roodenburg JL, Vissink A. Prosthodontic rehabilitation of oral function in head-neck cancer patients with dental implants placed simultaneously during ablative tumour surgery: an assessment of treatment outcomes and quality of life. Int J Oral Maxillofac Surg. 2008 Jan;37(1):8-16. doi: 10.1016/j.ijom.2007.07.015. Epub 2007 Sep 4. PMID: 17766084.

Alsaadi G, Quirynen M, Komárek A, van Steenberghe D. Impact of local and systemic factors on the incidence of late oral implant loss. Clin Oral Implants Res. 2008 Jul;19(7):670-6. doi: 10.1111/j.1600-0501.2008.01534.x. Epub 2008 May 19. PMID: 18492080.

Nelson K, Heberer S, Glatzer C. Survival analysis and clinical evaluation of implant-retained prostheses in oral cancer resection patients over a mean follow-up period of 10 years. J Prosthet Dent. 2007 Nov;98(5):405-10. doi: 10.1016/S0022-3913(07)60125-5. PMID: 18021829.

Schoen PJ, Raghoebar GM, Bouma J, Reintsema H, Vissink A, Sterk W, Roodenburg JL. Rehabilitation of oral function in head and neck cancer patients after radiotherapy with implant-retained dentures: effects of hyperbaric oxygen therapy. Oral Oncol. 2007 Apr;43(4):379-88. doi: 10.1016/j.oraloncology.2006.04.009. Epub 2006 Sep 25. PMID: 16996783.

Yerit KC, Posch M, Seemann M, Hainich S, Dörtbudak O, Turhani D, Ozyuvaci H, Watzinger F, Ewers R. Implant survival in mandibles of irradiated oral cancer patients. Clin Oral Implants Res. 2006 Jun;17(3):337-44. doi: 10.1111/j.1600-0501.2005.01160.x. PMID: 16672031.

Schepers RH, Slagter AP, Kaanders JH, van den Hoogen FJ, Merkx MA. Effect of postoperative radiotherapy on the functional result of implants placed during ablative surgery for oral cancer. Int J Oral Maxillofac Surg. 2006 Sep;35(9):803-8. doi: 10.1016/j.ijom.2006.03.007. Epub 2006 May 11. PMID: 16697146.

Landes CA, Kovács AF. Comparison of early telescope loading of non-submerged ITI implants in irradiated and non-irradiated oral cancer patients. Clin Oral Implants Res. 2006 Aug;17(4):367-74. doi: 10.1111/j.1600-0501.2005.01227.x. PMID: 16907766.

Bodard AG, Gourmet R, Lucas R, Bonnet E, Breton P. Implants dentaires en territoire irradié: série de 33 patients [Dental implants in irradiated areas: a series of 33 patients]. Rev Stomatol Chir Maxillofac. 2006 Jun;107(3):137-42; discussion 143-4. French. doi: 10.1016/s0035-1768(06)77007-3. PMID: 16804478.

Chiapasco M, Biglioli F, Autelitano L, Romeo E, Brusati R. Clinical outcome of dental implants placed in fibula-free flaps used for the reconstruction of maxillo-mandibular defects following ablation for tumors or osteoradionecrosis. Clin Oral Implants Res. 2006 Apr;17(2):220-8. doi: 10.1111/j.1600-0501.2005.01212.x. PMID: 16584419.

Teoh KH, Huryn JM, Patel S, Halpern J, Tunick S, Wong HB, Zlotolow IM. Implant prosthodontic rehabilitation of fibula free-flap reconstructed mandibles: a Memorial

Sloan-Kettering Cancer Center review of prognostic factors and implant outcomes. Int J Oral Maxillofac Implants. 2005 Sep-Oct;20(5):738-46. PMID: 16274148.

Shaw RJ, Sutton AF, Cawood JI, Howell RA, Lowe D, Brown JS, Rogers SN, Vaughan ED. Oral rehabilitation after treatment for head and neck malignancy. Head Neck. 2005 Jun;27(6):459-70. doi: 10.1002/hed.20176. PMID: 15880417.

Granström G. Osseointegration in irradiated cancer patients: an analysis with respect to implant failures. J Oral Maxillofac Surg. 2005 May;63(5):579-85. doi: 10.1016/j.joms.2005.01.008. PMID: 15883929.

Granström G. Radiotherapy, osseointegration and hyperbaric oxygen therapy. Periodontol 2000. 2003;33:145-62. doi: 10.1046/j.0906-6713.2002.03312.x. PMID: 12950848.

Cao Y, Weischer T. Comparison of maxillary implant-supported prosthesis in irradiated and non-irradiated patients. J Huazhong Univ Sci Technolog Med Sci. 2003;23(2):209-12. doi: 10.1007/BF02859961. PMID: 12973954.

Schoen PJ, Raghoebar GM, Vissink A, Roodenburg JL. Mandibulotomy and implant insertion. Head Neck. 2003 Sep;25(9):748-53. doi: 10.1002/hed.10284. PMID: 12953310.

Visch LL, van Waas MA, Schmitz PI, Levendag PC. A clinical evaluation of implants in irradiated oral cancer patients. J Dent Res. 2002 Dec;81(12):856-9. doi: 10.1177/154405910208101212. PMID: 12454102.

Van Steenberghe D, Jacobs R, Desnyder M, Maffei G, Quirynen M. The relative impact of local and endogenous patient-related factors on implant failure up to the abutment stage. Clin Oral Implants Res 2002;13:617–622.

Goto M, Jin-Nouchi S, Ihara K, Katsuki T. Longitudinal follow-up of osseointegrated implants in patients with resected jaws. Int J Oral Maxillofac Implants. 2002 Mar-Apr;17(2):225-30. PMID: 11958405.

Grötz KA, Wahlmann UW, Krummenauer F, Wegener J, al-Nawas B, Kuffner HD, Wagner W. Prognose und Prognosefaktoren enossaler Implantate im bestrahlten Kiefer [Prognosis and prognostic factors of endosseous implants in the irradiated jaw]. Mund Kiefer Gesichtschir. 1999 May;3 Suppl 1:S117-24. German. doi: 10.1007/PL00014497. PMID: 10414097.

Betz T, Purps S, Pistner H, Bill J, Reuther J. Orale Rehabilitation von Tumorpatienten mit enossalen Implantaten. Implantationserfolg unter besonderer Berücksichtigung der periimplantären Gewebe [Oral rehabilitation of tumor patients with endosseous implants. Implant success with special reference to peri-implant tissue]. Mund Kiefer Gesichtschir. 1999 May;3 Suppl 1:S99-105. German. doi: 10.1007/PL00014529. PMID: 10414094.

Weischer T, Mohr C. Ten-year experience in oral implant rehabilitation of cancer patients: treatment concept and proposed criteria for success. Int J Oral Maxillofac Implants. 1999 Jul-Aug;14(4):521-8. PMID: 10453667.

Schliephake H, Neukam FW, Schmelzeisen R, Wichmann M. Long-term results of endosteal implants used for restoration of oral function after oncologic surgery. Int J Oral Maxillofac Surg. 1999 Aug;28(4):260-5. PMID: 10416892.

Werkmeister R, Szulczewski D, Walteros-Benz P, Joos U. Rehabilitation with dental implants of oral cancer patients. J Craniomaxillofac Surg. 1999 Feb;27(1):38-41. doi: 10.1016/s1010-5182(99)80008-0. PMID: 10188126.

Mericske-Stern R, Perren R, Raveh J. Life table analysis and clinical evaluation of oral implants supporting prostheses after resection of malignant tumors. Int J Oral Maxillofac Implants. 1999 Sep-Oct;14(5):673-80. PMID: 10531739.

Keller EE, Tolman DE, Eckert SE. Maxillary antral-nasal inlay autogenous bone graft reconstruction of compromised maxilla: a 12-year retrospective study. Int J Oral Maxillofac Implants. 1999 Sep-Oct;14(5):707-21. PMID: 10531743.

Granström G, Tjellström A, Brånemark PI. Osseointegrated implants in irradiated bone: a case-controlled study using adjunctive hyperbaric oxygen therapy. J Oral Maxillofac Surg. 1999 May;57(5):493-9. doi: 10.1016/s0278-2391(99)90059-9. PMID: 10319821.

Foster RD, Anthony JP, Sharma A, Pogrel MA. Vascularized bone flaps versus nonvascularized bone grafts for mandibular reconstruction: an outcome analysis of primary bony union and endosseous implant success. Head Neck. 1999 Jan;21(1):66-71. doi: 10.1002/(sici)1097-0347(199901)21:1<66::aid-hed9>3.0.co;2-z. PMID: 9890353.

Esser E, Neukirchen S, Wagner W. Vergleichende Untersuchungen von Brånemark-Implantaten im bestrahlten und nicht bestrahlten Unterkiefer [Comparative studies of Brånemark implants in the irradiated and not irradiated mandible]. Mund Kiefer Gesichtschir. 1999 May;3 Suppl 1:S125-9. German. doi: 10.1007/PL00014498. PMID: 10414098.

Niimi A, Ueda M, Keller EE, Worthington P. Experience with osseointegrated implants placed in irradiated tissues in Japan and the United States. Int J Oral Maxillofac Implants. 1998 May-Jun;13(3):407-11. PMID: 9638012.

Ihara K, Goto M, Miyahara A, Toyota J, Katsuki T. Multicenter experience with maxillary prostheses supported by Brånemark implants: a clinical report. Int J Oral Maxillofac Implants. 1998 Jul-Aug;13(4):531-8. PMID: 9714960.

Brogniez V, Lejuste P, Pecheur A, Reychler H. Dental prosthetic reconstruction of osseointegrated implants placed in irradiated bone. Int J Oral Maxillofac Implants. 1998 Jul-Aug;13(4):506-12. PMID: 9714957.

Andersson G, Andreasson L, Bjelkengren G. Oral implant rehabilitation in irradiated patients without adjunctive hyperbaric oxygen. Int J Oral Maxillofac Implants. 1998 Sep-Oct;13(5):647-54. PMID: 9796148.

August M, Bast B, Jackson M, Perrott D. Use of the fixed mandibular implant in oral cancer patients: a retrospective study. J Oral Maxillofac Surg. 1998 Mar;56(3):297-301. doi: 10.1016/s0278-2391(98)90102-1. PMID: 9496839.

Wagner W, Esser E, Ostkamp K. Osseointegration of dental implants in patients with and without radiotherapy. Acta Oncol. 1998;37(7-8):693-6. doi: 10.1080/028418698430061. PMID: 10050989.

Niimi A, Ueda M, Keller EE, Worthington P. Experience with osseointegrated implants placed in irradiated tissues in Japan and the United States. Int J Oral Maxillofac Implants. 1998 May-Jun;13(3):407-11. PMID: 9638012.

Keller EE, Tolman DE, Zuck SL, Eckert SE. Mandibular endosseous implants and autogenous bone grafting in irradiated tissue: a 10-year retrospective study. Int J Oral Maxillofac Implants. 1997 Nov-Dec;12(6):800-13. PMID: 9425761.

Marker P, Siemssen SJ, Bastholt L. Osseointegrated implants for prosthetic rehabilitation after treatment of cancer of the oral cavity. Acta Oncol. 1997;36(1):37-40. doi: 10.3109/02841869709100729. PMID: 9090963.

Chan MF, Hayter JP, Cawood JI, Howell RA. Oral rehabilitation with implant-retained prostheses following ablative surgery and reconstruction with free flaps. Int J Oral Maxillofac Implants. 1997 Nov-Dec;12(6):820-7. PMID: 9425763.

Ali A, Patton DW, el-Sharkawi AM, Davies J. Implant rehabilitation of irradiated jaws: a preliminary report. Int J Oral Maxillofac Implants. 1997 Jul-Aug;12(4):523-6. PMID: 9274081.

Esser E, Wagner W. Dental implants following radical oral cancer surgery and adjuvant radiotherapy. Int J Oral Maxillofac Implants. 1997 Jul-Aug;12(4):552-7. PMID: 9274085.

Jisander S, Grenthe B, Alberius P. Dental implant survival in the irradiated jaw: a preliminary report. Int J Oral Maxillofac Implants. 1997 Sep-Oct;12(5):643-8. PMID: 9337025.

Roumanas ED, Markowitz BL, Lorant JA, Calcaterra TC, Jones NF, Beumer J 3rd. Reconstructed mandibular defects: fibula free flaps and osseointegrated implants. Plast Reconstr Surg. 1997 Feb;99(2):356-65. doi: 10.1097/00006534-199702000-00008. PMID: 9030140.

McGhee MA, Stern SJ, Callan D, Shewmake K, Smith T. Osseointegrated implants in the head and neck cancer patient. Head Neck. 1997 Dec;19(8):659-65. doi: 10.1002/(sici)1097-0347(199712)19:8<659:aid-hed3>3.0.co;2-4. PMID: 9406744.

Watzinger F, Ewers R, Henninger A, Sudasch G, Babka A, Woelfl G. Endosteal implants in the irradiated lower jaw. J Craniomaxillofac Surg. 1996 Aug;24(4):237-44. doi: 10.1016/s1010-5182(96)80007-2. PMID: 8880450.

Weischer T, Schettler D, Mohr C. Concept of surgical and implant-supported prostheses in the rehabilitation of patients with oral cancer. Int J Oral Maxillofac Implants. 1996 Nov-Dec;11(6):775-81. PMID: 8990640.

Eckert SE, Desjardins RP, Keller EE, Tolman DE. Endosseous implants in an irradiated tissue bed. J Prosthet Dent. 1996 Jul;76(1):45-9. doi: 10.1016/s0022-3913(96)90345-5. PMID: 8814634.

Aldegheri A, Beloni D, Blanc JL, Kaplanski P, Legre R, Zanaret M. La réhabilitation dentaire par fixtures ostéo-intégrables: traitement des cancers oro-maxillo-faciaux. Etude préliminaire de 7 cas [Dental rehabilitation using osseointegrated implants: treatment of oro-maxillo-facial cancer. A preliminary study of 7 cases]. Rev Stomatol Chir Maxillofac. 1996;97(2):108-16. French. PMID: 8685617.

Franzén L, Rosenquist JB, Rosenquist KI, Gustafsson I. Oral implant rehabilitation of patients with oral malignancies treated with radiotherapy and surgery without adjunctive hyperbaric oxygen. Int J Oral Maxillofac Implants. 1995 Mar-Apr;10(2):183-7. PMID: 7744437.

Sclaroff A, Haughey B, Gay WD, Paniello R. Immediate mandibular reconstruction and placement of dental implants. At the time of ablative surgery. Oral Surg Oral Med Oral Pathol. 1994 Dec;78(6):711-7. doi: 10.1016/0030-4220(94)90085-x. PMID: 7898907.

Albrektsson T, Dahl E, Enbom L, Engevall S, Engquist B, Eriksson AR, Feldmann G, Freiberg N, Glantz PO, Kjellman O, et al. Osseointegrated oral implants. A Swedish multicenter study of 8139 consecutively inserted Nobelpharma implants. J Periodontol. 1988 May;59(5):287-96. doi: 10.1902/jop.1988.59.5.287. PMID: 3290429.

S 3 – Justification for downgrading studies evaluated by the AMSTAR 2 tool

| Question | Study downgraded | Justification |
|---|---|---|
| Q.1 | Schiegnitz et al. (2022) e Schiegnitz et al. (2014) | Inclusion of participants with odontogenic tumors and cysts - not included in the PICO acronym |
| Q.2 | Shahi et al. (2023), Schiegnitz et al. (2022), Shokouhi and Cerajewska (2022), Gupta et al. (2021), In't Veld et al. (2021), Smith Nobrega et al. (2016); Chrcanovic et al. (2016) e Shiegnitz et al. (2014), Chambrone et al. (2013) | Did not state that the review methods were established before beginning the study |
| Q.3 | In't Veld et al. (2021) | They did not explain the selection of study designs for inclusion in the review |
| Q.4 | Camolesi et al. (2023), Shahi et al. (2023), Kende et al. (2022), Schiegnitz et al. (2022), Gupta et al. (2021), In't Veld et al. (2021), Smith Nobrega et al. (2016) e Shiegnitz et al. (2014) | They carried out language restrictions without justification |
| Q.5 | Shahi et al. (2023), Schiegnitz et al. (2022), Shokouhi and Cerajewska (2022) | They did not select studies in duplicate |
| Q.6 | Shahi et al. (2023), Schiegnitz et al. (2022), Shokouhi and Cerajewska (2022), In't Veld et al. (2021), Chrcanovic et al. (2016) | They did not perform data extraction in duplicate |
| Q.7 | Camolesi et al. (2023), Shahi et al. (2023), Kende et al. (2022), Schiegnitz et al. (2022), Shokouhi and Cerajewska (2022), Gupta et al. (2021), In't Veld et al. (2021), Smith Nobrega et al. (2016), Chrcanovic et al. (2016) e Shiegnitz et al. (2014) | They did not provide a list of excluded studies, justified |
| Q.8 | Shahi et al. (2023), Kende et al. (2022) | Did not describe the included studies in detail |
| Q.9 | In't Veld et al. (2021), Smith Nobrega et al. (2016); Chrcanovic et al. (2016) e Shiegnitz et al. (2014) | They did not use an adequate technique to assess the risk of bias in primary studies |
| Q.10 | Camolesi et al. (2023), Shahi et al. (2023), Kende et al. (2022), Schiegnitz et al. (2022), Shokouhi and Cerajewska (2022), Gupta et al. (2021), In't Veld et al. (2021), Smith Nobrega et al. (2016); Chrcanovic et al. (2016) e Shiegnitz et al. (2014), Chambrone et al. (2013) | They did not inform the source of funding in primary studies |
| Q.11 | Kende et al. (2022) | Meta-analysis results are not consistent |
| Q.12 | Shahi et al. (2023), Kende et al. (2022); Shiegnitz et al. (2014) | They did not assess the potential impact of the risk of bias in primary studies on the results of the meta-analysis |
| Q.13 | Camolesi et al. (2023), Shahi et al. (2023), Kende et al. (2022); Gupta et al. (2021) | They did not take into account the risk of bias in primary studies when discussing/interpreting the review results |

| | | |
|---|---|---|
| Q.14 | Kende et al. (2022) | They did not provide an explanation for the heterogeneity observed in the review results |
| Q.15 | Shokouhi and Cerajewska (2022), Gupta et al. (2021), In't Veld et al. (2021), Chambrone et al. (2013) | Did not investigate publication bias |
| Q.16 | Camolesi et al. (2023), Smith Nobrega et al. (2016); Chrcanovic et al. (2016), Shiegnitz et al. (2014) | Did not report any sources of conflicts of interest |

Q.1. Did the research questions and inclusion criteria for the review include the components of PICO? Q.2. Did the report of the review contain an explicit statement that the review methods were established prior to the conduct of the review and did the report justify any significant deviations from the protocol? Q. 3. Did the review authors explain their selection of the study designs for inclusion in the review? Q. 4. Did the review authors use a comprehensive literature search strategy? Q. 5. Did the review authors perform study selection in duplicate? Q. 6. Did the review authors perform data extraction in duplicate? Q. 7. Did the review authors provide a list of excluded studies and justify the exclusions? Q. 8. Did the review authors describe the included studies in adequate detail? Q. 9. Did the review authors use a satisfactory technique for assessing the risk of bias (RoB) in individual studies that were included in the review? Q. 10. Did the review authors report on the sources of funding for the studies included in the review? Q. 11. If meta-analysis was performed, did the review authors use appropriate methods for statistical combination of results? Q. 12. If meta-analysis was performed, did the review authors assess the potential impact of RoB in individual studies on the results of the meta-analysis or Other evidence synthesis? Q. 13. Did the review authors account for RoB in primary studies when interpreting/discussing the results of the review? Q. 14. Did the review authors provide a satisfactory explanation for, and discussion of, any heterogeneity observed in the results of the review? Q. 15. If they performed quantitative synthesis did the review authors carry out an adequate investigation of publication bias (small study bias) and discuss its likely impact on the results of the review? Q.16. Did the review authors report any potential sources of conflict of interest, including any funding they received for conducting the review?

Figure

Click here to access/download;Figure;renamed_b84fb.png

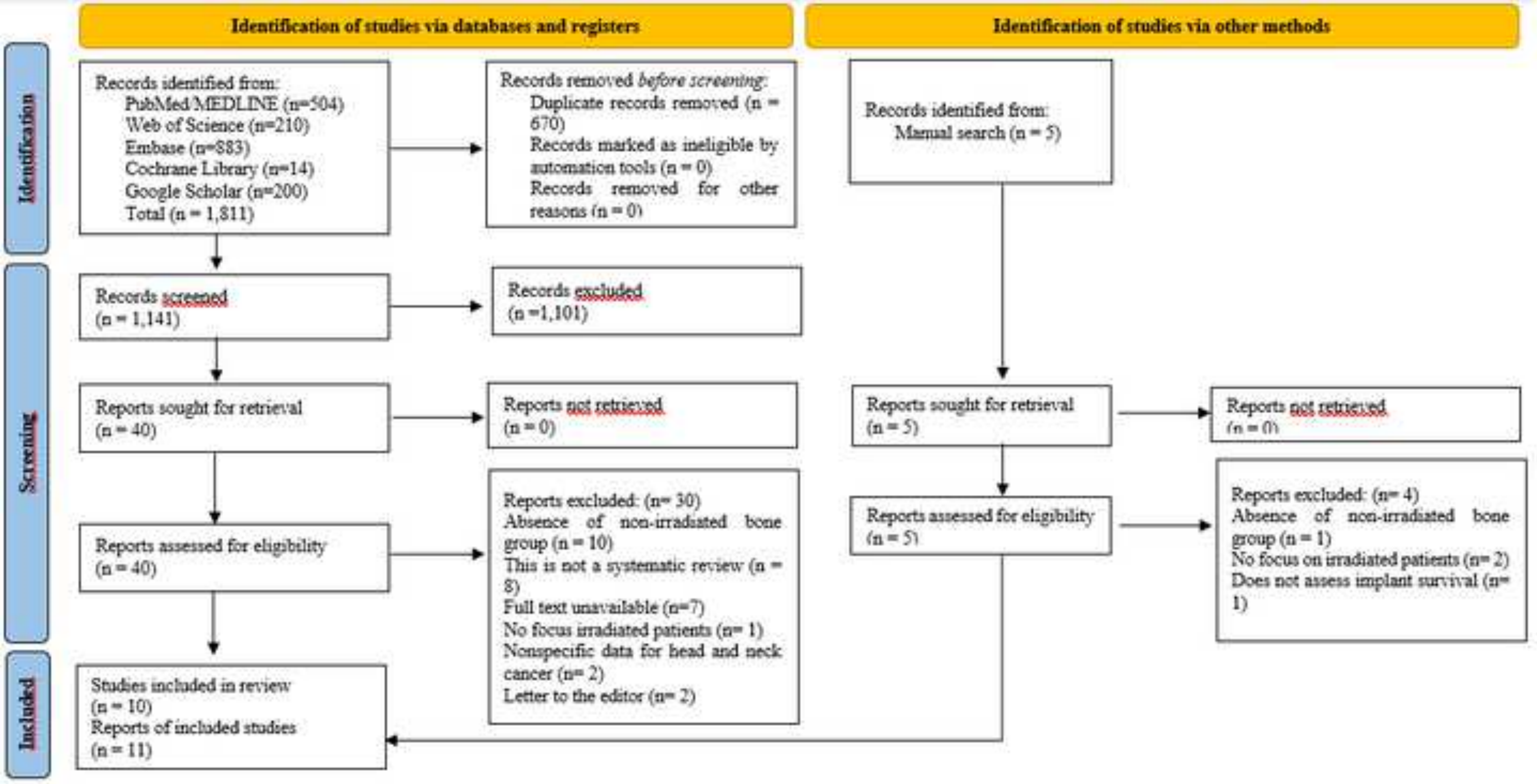

Click here to access/download
Supporting Information
renamed_99c97.docx
