# Supplementary material for: Survival of dental implants in irradiated head and neck cancer patients compared to non-irradiated patients: An umbrella review
Source: PLoS One. 2025 Sep 3;20(9):e0324388. doi: 10.1371/journal.pone.0324388 (PMC12407548; doi:10.1371/journal.pone.0324388)
Supplement: Attachment — Submitted filename: PONE-D-24-47771_R1_reviewer comments added.pdf [file pone.0324388.s004.pdf]

PLOS ONE

Survival of dental implants in irradiated head and neck cancer patients compared to non-irradiated patients: An umbrella review

--Manuscript Draft--

Manuscript Number:	PONE-D-24-47771R1
Article Type:	Research Article
Full Title:	Survival of dental implants in irradiated head and neck cancer patients compared to non-irradiated patients: An umbrella review
Short Title:	Survival of dental implants in irradiated head and neck cancer patients compared to non-irradiated patients
Corresponding Author:	Caio Fernando Teixeira Portela, Ph.D<br>Universidade Federal de Minas Gerais Escola de Engenharia<br>Belo Horizonte, BRAZIL
Keywords:	Keywords: Dental implants, radiotherapy, head and neck cancer, systematic review, umbrella review.
Abstract:	Background: There is an increasing demand for oral rehabilitation in patients undergoing irradiation for head and neck cancer treatment. Although radiotherapy appears to adversely affect implanted oral rehabilitation, the evidence provided by previous systematic reviews in the field remains controversial. Thus, this umbrella review aimed to evaluate the survival of dental implants installed before and/or after radiotherapy in patients with head and neck cancer compared to those not irradiated. Methods: A comprehensive search of electronic databases was performed in the Medline/PubMed, Cochrane Library, Embase, Web of Science, and Google Scholar databases, including manual searches, from inception to February, 2024, with no language or date restrictions. The Preferred Reporting Items for Systematic Reviews and Meta-Analyses (PRISMA) 2020 guidelines were followed. Survival percentage of dental implants was the primary outcome. A MeaSurement Tool to Assess systematic Reviews (AMSTAR) 2 was used as a critical appraisal tool for the included studies. The protocol for this review has been registered with PROSPERO (CRD42023406059). Results: Of the 1,811 articles screened, 11 articles that evaluated 73,674 implants were included. Quantitative analyses showed 2,674 failures out of 14,471 implants installed in irradiated bone and 1,825 failures out of 34,092 implants in non-irradiated bone. The survival rate was 81.52% for irradiated implants and 94.64% for non-irradiated implants. A significant difference in survival in favor of implants in non-irradiated bone is supported by 11 meta-analyses. The included systematic reviews showed critically low methodological quality. Conclusions: Despite the critically low methodological quality of the included studies, implants installed in irradiated patients had a higher percentage of failures. Further clinical studies employing improved research designs are warranted.
Order of Authors:	Caio Fernando Teixeira Portela, Ph.D
	Maria Cândida Dourado Pacheco, Doctorate
	Alexandre Henrique dos Reis Prado, Doctorate
	Tarcísio Passos Ribeiro Campos, Doctorate
	Lara Cancella de Arantes, Master
	Arno Heeren de Oliveira, Doctorate
	Danilo Viegas da Costa, Master
Response to Reviewers:	Done
Additional Information:	
Question	Response
Financial Disclosure	The author(s) received no specific funding for this work.

Enter a financial disclosure statement that describes the sources of funding for the work included in this submission. Review the submission guidelines for detailed requirements. View published research articles from *PLOS ONE* for specific examples.<br><br>This statement is required for submission and **will appear in the published article** if the submission is accepted. Please make sure it is accurate.<br><br>**Funded studies**<br>Enter a statement with the following details:<br>• Initials of the authors who received each award<br>• Grant numbers awarded to each author<br>• The full name of each funder<br>• URL of each funder website<br>• Did the sponsors or funders play any role in the study design, data collection and analysis, decision to publish, or preparation of the manuscript?<br><br>Did you receive funding for this work?	
**Competing Interests**<br><br>Use the instructions below to enter a competing interest statement for this submission. On behalf of all authors, disclose any competing interests that could be perceived to bias this work—acknowledging all financial support and any other relevant financial or non-financial competing interests.<br><br>This statement is required for submission and **will appear in the published article** if the submission is accepted. Please make sure it is accurate and that any funding sources listed in your Funding Information later in the submission form are also declared in your Financial Disclosure statement.	The authors have declared that no competing interests exist

View published research articles from *PLOS ONE* for specific examples.<br><br>**NO authors have competing interests**<br><br>Enter: *The authors have declared that no competing interests exist.*<br><br>**Authors with competing interests**<br><br>Enter competing interest details beginning with this statement:<br><br>*I have read the journal's policy and the authors of this manuscript have the following competing interests: [insert competing interests here]*<br><br>* typeset	
**Ethics Statement**<br><br>Enter an ethics statement for this submission. This statement is required if the study involved:<br><br>• Human participants<br>• Human specimens or tissue<br>• Vertebrate animals or cephalopods<br>• Vertebrate embryos or tissues<br>• Field research<br><br>Write "N/A" if the submission does not require an ethics statement.<br><br>General guidance is provided below. Consult the submission guidelines for detailed instructions. **Make sure that all information entered here is included in the Methods section of the manuscript.**	N/A

**Format for specific study types**<br><br>**Human Subject Research (involving human participants and/or tissue)**<br>• Give the name of the institutional review board or ethics committee that approved the study<br>• Include the approval number and/or a statement indicating approval of this research<br>• Indicate the form of consent obtained (written/oral) or the reason that consent was not obtained (e.g. the data were analyzed anonymously)<br><br>**Animal Research (involving vertebrate animals, embryos or tissues)**<br>• Provide the name of the Institutional Animal Care and Use Committee (IACUC) or other relevant ethics board that reviewed the study protocol, and indicate whether they approved this research or granted a formal waiver of ethical approval<br>• Include an approval number if one was obtained<br>• If the study involved *non-human primates*, add *additional details* about animal welfare and steps taken to ameliorate suffering<br>• If anesthesia, euthanasia, or any kind of animal sacrifice is part of the study, include briefly which substances and/or methods were applied<br><br>**Field Research**<br><br>Include the following details if this study involves the collection of plant, animal, or other materials from a natural setting:<br>• Field permit number<br>• Name of the institution or relevant body that granted permission	
**Data Availability**<br><br>Authors are required to make all data underlying the findings described fully available, without restriction, and from the time of publication. PLOS allows rare exceptions to address legal and ethical concerns. See the PLOS Data Policy and FAQ for detailed information.	Yes - all data are fully available without restriction

A Data Availability Statement describing where the data can be found is required at submission. Your answers to this question constitute the Data Availability Statement and **will be published in the article**, if accepted.<br><br>**Important:** Stating 'data available on request from the author' is not sufficient. If your data are only available upon request, select 'No' for the first question and explain your exceptional situation in the text box.<br><br>Do the authors confirm that all data underlying the findings described in their manuscript are fully available without restriction?	
**Describe where the data may be found in full sentences. If you are copying our sample text, replace any instances of XXX with the appropriate details.**<br><br>• If the data are **held or will be held in a public repository**, include URLs, accession numbers or DOIs. If this information will only be available after acceptance, indicate this by ticking the box below. For example: *All XXX files are available from the XXX database (accession number(s) XXX, XXX.*).<br>• If the data are all contained **within the manuscript and/or Supporting Information files**, enter the following: *All relevant data are within the manuscript and its Supporting Information files.*<br>• If neither of these applies but you are able to provide **details of access elsewhere**, with or without limitations, please do so. For example:<br><br>*Data cannot be shared publicly because of [XXX]. Data are available from the XXX Institutional Data Access / Ethics Committee (contact via XXX) for researchers who meet the criteria for access to confidential data.*<br><br>*The data underlying the results presented in the study are available from (include the name of the third party*	All relevant data are within the manuscript and its Supporting Information files.

and contact information or URL).<br>• This text is appropriate if the data are owned by a third party and authors do not have permission to share the data.<br><br>* typeset	
Additional data availability information:	Tick here if the URLs/accession numbers/DOIs will be available only after acceptance of the manuscript for publication so that we can ensure their inclusion before publication.; Tick here if your circumstances are not covered by the questions above and you need the journal's help to make your data available.

Manuscript

Click here to access/download;Manuscript;plos_latex_template_v3_6__1_

Survival of dental implants in irradiated head and neck cancer patients compared to non-irradiated patients: An umbrella review

Maria Cândida Dourado Pacheco[1◑], Caio Fernando Teixeira Portela[1◑], Alexandre Henrique dos Reis-Prado[3◑], Lara Cancella de Arantes[2◑], Danilo Viegas da Costa[4◑], Tarcísio Passos Ribeiro Campos[1◑], Arno Heeren de Oliveira[1◑]

1 Department of Nuclear Engineering, Federal University of Minas Gerais, School of Engineering, Belo Horizonte, Minas Gerais, Brazil
2 Restorative Dentistry, Federal University of Minas Gerais, School of Dentistry, Belo Horizonte, Minas Gerais, Brazil
3 Restorative Dentistry, Federal University of Minas Gerais, School of Dentistry, Belo Horizonte, Minas Gerais, Brazil
4 Dental Clinics with an emphasis on Periodontics at the Pontifical Catholic University of Minas Gerais, Belo Horizonte, Minas Gerais, Brazil

◑These authors contributed equally to this work.
‡These authors also contributed equally to this work.
¤Current Address: Dept/Program/Center, Institution Name, City, State, Country
†Deceased
¶Membership list can be found in the Acknowledgments section.
$*caiofernando_fisica@yahoo.com.br$

Abstract

Background: There is an increasing demand for oral rehabilitation in patients undergoing irradiation for head and neck cancer treatment. Although radiotherapy appears to adversely affect implanted oral rehabilitation, the evidence provided by previous systematic reviews in the field remains controversial. Thus, this umbrella review aimed to evaluate the survival of dental implants installed before and/or after radiotherapy in patients with head and neck cancer compared to those not irradiated. **Methods:** A comprehensive search of electronic databases was performed in the Medline/PubMed, Cochrane Library, Embase, Web of Science, and Google Scholar databases, including manual searches, from inception to February, 2024, with no language or date restrictions. The Preferred Reporting Items for Systematic Reviews and Meta-Analyses (PRISMA) 2020 guidelines were followed. Survival percentage of dental implants was the primary outcome. A MeaSurement Tool to Assess systematic Reviews (AMSTAR) 2 was used as a critical appraisal tool for the included studies. The protocol for this review has been registered with PROSPERO (CRD42023406059). **Results:** Of the 1,811 articles screened, 11 articles that evaluated 73,674 implants were included. Quantitative analyses showed 2,674 failures out of 14,471 implants installed in irradiated bone and 1,825 failures out of 34,092 implants in non-irradiated bone. The survival rate was 81.52% for irradiated implants and 94.64% for non-irradiated implants. A significant difference in survival in favor of implants in non-irradiated bone is supported by 11 meta-analyses. The included systematic reviews showed critically low methodological quality. **Conclusions:** Despite the critically low methodological quality of the included studies, implants installed in irradiated patients had a higher percentage of failures. Further clinical studies employing improved research designs are warranted.

Author summary

Graduated in Dentistry from the Federal School of Dentistry of Diamantina (1993). PhD in Nuclear Sciences and Techniques from UFMG (2024), with a focus on radiation sciences. Master's degree in Dentistry, specializing in implantology, from PUC-Minas (2014). Specialist in Implantology (PUC-Minas/2014). Specialist in Periodontics from FAFEOD (2000). Currently works as an administrative technician and dentist at the Federal Institute of Northern Minas Gerais - Januária campus. Has experience in the field of Dentistry, with an emphasis on implantology, periodontics, and prosthetics.

Introduction

Osseointegrated dental implants are widely used and can provide a safe treatment option for edentulous patients, demonstrating promising survival rates within a 5 to 10-year follow-up period (Jung et al., 2012) [1]. However, the risks associated with implant failures have been extensively investigated over the years (Papi et al., 2019; Albrega et al., 2020; Pieralli et al., 2021; Li et al., 2022) [2, 3, 4, 5]. Possible reasons for failure are low insertion torque of implants planned to be loaded immediately or early, inexperienced surgeons inserting the implants, insertion of the implant into the maxilla, the patient history (radiotherapy, bruxism, periodontitis and early implant loss) (Chrcanovic et al., 2014; Do et al., 2020) [6, 7].

There is an increasing demand for oral rehabilitation in patients undergoing irradiation for head and neck cancer treatment (Chrcanovic et al., 2016) [8]. Cases of head and neck cancer are significantly increasing among the population (Sung et al., 2021) [9], which is also associated with the increased frequency of dental implants installed in these patients (Shiegnitz et al., 2021) [10]. However, radiotherapy seems to adversely affect implanted oral rehabilitation (Ernst et al., 2016; Neckel et al., 2021; Wolf et al., 2021) [11, 12, 13] due to radiation-induced fibroatrophic process (Delanian and Lefaix, 2004) [14], leading to tissue hypoxia, hypocellularity, hypovascularity and reduced bone regeneration capacity (Marx; Johnson, 1987) [15].

Although several systematic reviews have investigated the survival of dental implants in head and neck cancer patients undergoing radiotherapy involving various clinical analyses, the presented results have been inconclusive (In't Veld et al., 2021; Shiegnitz et al., 2022; Kende et al., 2022). [16, 17, 18] Recently evaluated in two umbrella reviews (Marques et al., 2023; Zarzar et al., 2024) [19, 20], oral rehabilitation in patients with irradiated head and neck cancer is a valid therapy. However, due to heterogeneity and the lack of a comparator group in most of the included systematic reviews, the recommendation is that the results presented be interpreted with caution. The present umbrella review aimed to analyse the available relevant systematic reviews, comparing irradiated versus non-irradiated patients, in an attempt to determine whether there is an influence of head and neck radiotherapy on dental implant survival in patients with head and neck cancer.

Materials and methods

This study followed the guidelines of the Preferred Reporting Items for Systematic Reviews and Meta-Analyses (PRISMA) 2020 (Page et al., 2021) [21]. The research protocol was registered on the International Prospective Register of Systematic Reviews (PROSPERO) database under the number CRD42023406059.

2.1 PICO formulation

The PICO (Population; Intervention; Comparison; Outcome) strategy was used to formulate the following research question: ”What is the survival of dental implants installed before and/or after radiotherapy in patients with head and neck cancer, compared to those not exposed to radiation?” (Table 1).

2.2 Eligibility Criteria

The inclusion criterion were systematic reviews, with or without meta-analysis, that assessed the survival of dental implants installed before and/or after radiotherapy in patients with head nd neck cancer, compared to patients with head and neck cancer in whom implants were installed in non-irradiated bone. The exclusion criteria were as follows: (1) systematic reviews of animal studies, (2) non-systematic reviews and editorials, (3) studies not focusing on the PICO question, and (4) studies with control groups not consisting non-irradiated individuals, and (3) studies for which the full text was unavailable. No restrictions were applied regarding the language or year of publication of the articles.

2.3 Research Sources and Search Strategy

The electronic search was conducted by two independent reviewers (MCDP and LCA), including studies published in PubMed/MEDLINE, Cochrane Library, Embase, and Web of Science, from their inception until February 23, 2024. Additionally, grey literature search was searched in Google Scholar database. Search strategies were developed for each database using indexed terms and synonyms (Supporting Information S1). A manual search of references was performed in all included studies. In case of doubt, a third reviewer (DVC) was consulted. There were no restrictions on language or year of publication for the articles.

2.4 Study Selection Process

The selection of studies was conducted by two reviewers (MCDP and LCA) independently, in a two-stage process using the Ryyan app (Qatar Foundation, Qatar) (Ouzzani et al., 2016) [22]. Duplicate studies were excluded using reference management software (EndNote® X9; Clarivate, Philadelphia, PA, USA). In the first stage, titles and abstracts were assessed, and in the next stage, the full text selected from the titles/abstracts was read, and those that did not meet the eligibility criteria were excluded. Disagreements were resolved by a third reviewer (DVC). Contacting authors to clarify doubts was not necessary. The level of agreement between the reviewers was assessed by Cohen’s Kappa (Landis and Koch, 1977) [23].

2.5 Data Extraction and Analysis

Data were collected by two reviewers (MCDP and LCA) independently in April 2024, based on the main characteristics of the studies, in an Excel form created and customized for the current review and included: study and publication year, number of participants/age range, country of publication, number of included studies/study design, presence of meta-analysis, variation in radiation dose (Gy)/implant installation time, number of failed/installed implants, implantation site, time interval between radiotherapy and implant installation, follow-up period, implant characteristics, cancer characteristics, adjuvant therapy, bone crest stability, postoperative complications, prosthetic rehabilitation. Subsequently, a third author (DVC) reviewed the data. In cases of missing data, authors would be contacted by email twice.

2.6 Assessment of Methodological Quality

The quality of included systematic reviews was appraised by using the A MeaSurement Tool to Assess Systematic Reviews (AMSTAR 2) tool (Shea et al., 2017) [24], and it was independently assessed by two reviewers (MCDP and LCA). Disagreements were resolved by a third reviewer (DVC). The methodological quality of systematic reviews was assessed using the, which consists of 16 items, where items 2, 4, 7, 9, 11, 13, and 15 are considered critical. Questions are answered with options: yes, partial yes, no, or meta-analysis not conducted. The overall confidence rating of the review's results is categorized as high (none or one non-critical weakness), moderate (more than one non-critical weakness), low (one critical flaw with or without non-critical weaknesses), or critically low (more than one critical flaw with or without non-critical weaknesses).

2.7 Results Synthesis

A descriptive synthesis of the results of the included studies was conducted. If a study did not provide raw data on implant failure but presented percentages related to implant survival, the data were converted when necessary. The percentage of implant survival was calculated by arithmetic mean.

Results

3.1 Study Selection

The PRISMA flowchart depicting the study selection process is presented in Figure 1. The literature search strategy identified 1,811 articles. After removing 670 duplicates, the remaining 1,141 publications were evaluated by reading titles/abstracts. A total of five additional reports were identified through manual search. This resulted in 45 records selected for reading the full text. Thirty-four references were excluded because 11 did not investigate the control group - non-irradiated (Benites Condezo et al., 2021; Schimmel et al., 2018; Ravi et al., 2017; Zhang et al., 2016; Shugaa Addin et al., 2016; Zen Filho et al., 2016; Claudy et al., 2015; Esposito and Worthington, 2013; Nooh, 2013; Esposito et al., 2008; Coulthard et al., 2002) [25, 26, 27, 28, 29, 30, 31, 32, 33, 34, 35], eight were not systematic reviews (Koudougou et al., 2020; Thanya and Ganapathy, 2016; Tanaka et al., 2013; Dholam and Gurav, 2012; Javed et al., 2010; Schoen et al., 2004; Harrison et al., 2003; Marx and Morales, 1998) [36, 37, 38, 39, 40, 41, 42, 43], in 7 references the full text was unavailable (Atanásio Pitorro et al., 2022; Estafanous et al., 2022; Recani et al., 2014; Garg and Guez, 2011; Hugentobl and Guyot, 2009; Colella et al., 2007; Samouhi and Buchbinder, 2000) [44, 45, 46, 47, 48, 49, 50], three did not focus on oral rehabilitation with implants in irradiated patients (Lu et al., 2021; Kotsakis et al., 2015; Chen et al., 2013) [51, 52, 53], two included nonspecific data for head and neck cancer (Toneatti et al., 2021; Panchal et al., 2020) [54, 55]; two were editorials (Korfage et al., 2015; Schiegnitz et al., 2015) [56, 57], and one did not investigate implant survival (Barber et al., 2011) [58]. Finally, eleven systematic reviews were included for qualitative synthesis. A table of all studies identified in the literature search, including those that were excluded from the analyses with the reasons for exclusion is presented in Supporting Information S2. The inter-rater kappa coefficients indicated an almost perfect agreement level among reviewers during study selection, with values greater than 0.800 for the databases.

3.2 Main characteristics of included reviews

Tables 2 and 3 show the characteristics of the eligible systematic reviews. A total of 16,193 patients who received 73,674 dental implants were included. Of these, approximately 31,137 were installed in irradiated sites. All selected studies (Camolesi et al., 2023; Shahi et al., 2023; Kende et al., 2022; Schiegnitz et al., 2022; Shokouhi and Cerajewska, 2022; Gupta et al., 2021; In't Veld et al., 2021; Smith Nobrega et al., 2016; Chrcanovic et al., 2016; Chambrone et al., 2013) [65, 66, 24, 23, 67, 68, 22, 69, 14, 70] assessed the survival of dental implants installed after the diagnosis of head and neck cancer. One systematic review (Shiegnitz et al., 2022) [23] was an update of a previous study elaborated by the same research team (Shiegnitz et al., 2014) [71]. The age range of participants varied from 6 to 94 years, with one study not reporting the sample age (Chambrone et al., 2013) [70]. Among the included reviews, most evaluated data from retrospective studies. All included studies conducted meta-analysis.

The minimum radiation dose among the studies ranged from 10 Gy to 40 Gy and the maximum dose from 72 Gy to 145 Gy; two studies did not mention the radiation dose (Shahi et al., 2023; Chambrone et al., 2013) [66, 70]. Regarding the timing of implant installation, five articles described implants installed before and after radiotherapy (Kende et al., 2022; Schiegnitz et al., 2022; Smith Nobrega et al., 2016; Chrcanovic et al., 2016; Chambrone et al., 2013) [24, 23, 69, 14, 70], four reported implants installed after radiotherapy (Camolesi et al., 2023; Shahi et al., 2023; Shokouhi and Cerajewska, 2022; Gupta et al., 2021) [65, 66, 67, 68]; one study evaluated implant installation during panendoscopy or ablative surgery - before radiotherapy (In't Veld et al., 2021) [22]. All selected studies evaluated the survival of implants installed in the maxilla and mandible. While seven studies investigated implants installed in native and grafted bone (Camolesi et al., 2023; Kende et al., 2022; Schiegnitz et al., 2022; Gupta et al., 2021; In't Veld et al., 2021; Smith Nobrega et al., 2016; Chrcanovic et al., 2016) [65, 24, 23, 68, 22, 69, 14], two analyzed implants installed only in native bone (Shokouhi and Cerajewska, 2022; Chambrone et al., 2013) [67, 70], and one study did not mention (Shahi et al., 2023) [66]. Only one study investigated the time interval between radiotherapy and implantation (Chrcanovic et al., 2016) [4]. The follow-up time among the studies varied from one month to 23 years, with one study not mentioning the follow-up period (Shahi et al., 2023) [66]. More details regarding the study design, number of implants installed, number of failed implants, and implant survival percentage extracted from the primary articles are described in Supporting Information S3.

3.3 Synthesis of results

Survival percentage of dental implants in irradiated and non-irradiated patients. The survival rate of implants was higher among non-irradiated patients according to data from 11 meta-analyses. Conversely, three meta-analyses showed no significant difference between the irradiated and non-irradiated groups (Kende et al., 2022; In't Veld et al., 2021; Shiegnitz et al., 2014 – literature between 2007-2013). Data were reported in four time periods (1990-2021, 1990-2006, 2007-2013, and 2013-2021) [18, 16, 65] and two follow-up periods ($\geq$ 3 years of follow-up and $\geq$ 5 years of follow-up). The effect estimate and precision of each meta-analysis are presented in Table 4. Further information on the number of failed/installed implants, number of primary articles included in the meta-analysis, and the percentage of survival is also shown in Table 4. Overall, 48,563 implants were evaluated, including 14,471 with 2,674 failures in the irradiated group and 34,092 with 1,825 failures in the non-irradiated group. The survival percentage was 81.52% and 94.64%, respectively.

Survival percentage of Implants Installed in Irradiated Maxilla versus Irradiated Mandible

Two meta-analyses (Chrcanovic et al., 2016; Chambrone et al., 2013) [8, 64] showed higher implant survival installed in irradiated mandible compared to irradiated maxilla (RR: 3.16; 95% CI: 1.76-5.68; p=0.0001; I2=70%) (RR: 5.96; 95% CI: 2.71-13.12; p¡0.00001; I2=33%). A total of 3,378 implants were evaluated, including 897 with 188 failures in the irradiated maxilla group and 2,481 with 160 failures in the irradiated mandible group. The survival percentage was 79.04% and 93.55%, respectively.

In the study by Shokouhi and Cerajewska (2022) [61], implants installed in the maxilla were significantly more likely to fail than those installed in the mandible (OR: 5.03; 95% CI: 1.07-23.58; p=0.04; I2=87%). Survival of Implants Installed in Irradiated Grafted Bone versus Irradiated Native Bone Two meta-analyses (Shiegnitz et al., 2022 – literature 1990-2021) showed higher implant survival in irradiated native bone [17]. (OR: 2.06; 95% CI: 1.44-2.94; p¡0.0001; I2=0% - follow-up $\geq$ 36 months) (OR: 2.26; 95% CI: 1.50-3.40; p¡0.0001; I2=12% - follow-up $\geq$ 60 months).

Conversely, one meta-analysis (Chrcanovic et al., 2016) [8] showed no significant difference between irradiated grafted bone and irradiated native bone (RR: 1.35; 95% CI: 0.93-1.94; p=0.11; I2=48%). A total of 1,740 implants were evaluated, including 596 with 106 failures in the irradiated grafted bone group and 1,144 with 148 failures in the irradiated native bone group. The survival percentage was 82.21% and 87.06%, respectively.

Regarding the time interval between the end of radiotherapy and implant installation (up to 12 months versus after 12 months), and the evaluation of radiation dose (high doses versus low doses), there was no influence of these variables on implant failure among the evaluated groups (Chrcanovic et al., 2016) [8].

3.4 Methodological Quality of Systematic Reviews

Overall, according to the evaluated domains using the AMSTAR 2 tool, the eligible reviews exhibited critically low quality (Table 5). The justifications for downgrading the studies are detailed in the Supporting Information S4.

Discussion

The present umbrella review included eleven systematic reviews published between 2013 and 2023, assessing the survival of 14,471 dental implants installed before and/or after radiotherapy in head and neck cancer patients, compared to 34,092 implants in non-irradiated patients. Overall, the survival rate of implants installed in irradiated patients was considered inferior compared to those installed in non-irradiated patients. Implant survival in the irradiated maxilla was significantly lower compared to the irradiated mandible. Dental implants installed in irradiated grafted bone were considered more prone to failure compared to irradiated native bone. The methodological quality of the selected studies was considered critically low.

Evidence from systematic reviews supports that implant survival is influenced by the effect of radiotherapy (Panchal et al., 2020; Toneatti et al., 2021) [55, 54]. Zarzar et al., (2024) [20], in an umbrella review, demonstrated that the implant survival rate in irradiated patients was lower compared to non-irradiated patients, with follow-up ranging from 1 month to 23 years. The present umbrella review evaluated and summarized scientific evidence available in the literature, corroborating previous reviews. It is important to mention that the analyzed data had different follow-up periods, which could cause significant errors in the descriptive synthesis presented in this study.

The impact of radiation dose on the survival of dental implants installed in head and neck cancer patients depends on the specific dose at the implant site (¿50 Gy) and the parotid gland (¿30 Gy) (Wolf et al., 2021) [13]. An umbrella review reported that radiation doses lower than 50 Gy were associated with better survival rates (Marques et al., 2023) [19]. This is consistent with the results observed in this umbrella review. It is important to consider that these data compare high doses with low doses, not detailing the implantation site with the target volume of radiation. Moreover, the systematic reviews included studies that varied the maximum doses from 72 Gy to 145 Gy, which may represent a significant risk of implant failure and osteoradionecrosis (Anderson et al., 2013) [66].

Most implants evaluated in this review were installed after the completion of radiotherapy. Implants installed after the end of radiotherapy were significantly more likely to fail than those installed in non-irradiated bone (Gupta et al., 2021; Shokouhi and Cerajewka, 2021) [62, 61]. Whereas implants installed immediately during ablative surgery (pre-RT) showed no statistically significant difference between irradiated and non-irradiated groups (In't Veld et al., 2021) [16]. There is a trend to install the implant during tumor removal, significantly reducing prosthetic rehabilitation time and, on the other hand, increasing complications that may hinder implantation success, such as obtaining a well-positioned implant (Di Carlo et al., 2019; Patel et al., 2020; Albrega et al., 2020) [67, 68, 3].

The time interval between radiotherapy and implant surgery may influence implant survival. A meta-analysis showed no statistically significant difference when comparing implant failures installed before and after 12 months of radiotherapy (Chrcanovic et al., 2016) [8]. Claudy et al. (2013) [31], in a meta-analysis, evaluated the time interval between 6 and 12 months after radiotherapy versus 12 months after radiotherapy, and an increased risk of 34% was observed for implants installed between 6 and 12 months post-radiotherapy. The literature recommends implant installation at least six months after the completion of radiotherapy, the expected period for the end of the acute process in bone and soft tissues induced by radiation (Marx and Johnson, 1987) [15].

The umbrella reviews, Zarzar et al. (2024) [20] and Marques et al. (2023) [19], using the AMSTAR 2 tool, included five systematic reviews in common with this study (Chambrone et al., 2013; Shiegnitz et al., 2014; Chrcanovic et al., 2016; Smith Nobrega et al., 2016; In't Veld et al., 2021) [64, 65, 8, 63, 16]. Zarzar et al. (2024) [20] classified the systematic reviews of Shiegnitz et al. (2014) [65], Smith Nobrega et al. (2016) [63], and In't Veld et al. (2021) [16] as critically low, which is in line with the results observed in this study. In contrast, the systematic review by Chambrone et al. (2013) [64] was considered high quality and Chrcanovic et al. (2016) [8] as low quality. This difference could be explained by the complexity associated with judging the issues. Marques et al. (2023) [19] analyses combined responses to the 16 individual items and performed an overall score to assess the risk of bias of the studies, making the classification criteria different from the assessments of this study.

The present study investigated irradiated head and neck cancer patients, recommending caution in interpreting the results, despite the high survival rate demonstrated for dental implants. The evaluation of methodological quality, through the AMSTAR 2 tool, showed that although the included studies reported the use of the PRISMA guideline, only two systematic reviews reported prior registration of the research protocol (Camolesi et al., 2023; Kende et al., 2022) [59, 18]. The PRISMA guideline itself advises indicating where the review protocol can be accessed to allow readers to assess whether any deviations from the protocol compared to what was done may have introduced bias. Some limitations should be considered when interpreting the results of this umbrella review. The search strategy identified only eleven systematic reviews, all classified with critically low methodological quality. Considering the

retrospective nature of most primary studies included in the selected systematic reviews, it was observed that the reported data were notably heterogeneous, especially for questions related to implant characteristics, radiation, and follow-up period. The effect measure used in conducting the selected systematic reviews was the number of implants that failed and the number of implants installed, not including details about the patient or radiation to answer the research question. Additionally, implant surface properties are confounding factors to be considered. In this sense, some studies (Chambrone et al., 2013; Chrcanovic et al., 2016; Smith Nobrega et al., 2016; In't Veld et al., 2021; Gupta et al., 2021) [64, 8, 63, 16, 62] sought to evaluate the geometry and surface treatment of the implant. However, it was not possible to reach a consensus on the risk of implant failures based on their characteristics because most individual studies did not report these data.

The main finding of this study was that, for patients after the diagnosis of head and neck cancer, the survival of implants that were irradiated before and/or after implantation was lower than that of implants installed in non-irradiated patients. We recommend that future randomized controlled trials be conducted on the topic. On the other hand, we consider the difficulty involved in conducting controlled and randomized experiments in this population. Therefore, new prospective and retrospective observational studies are needed. The literature is underexplored regarding the influence of radiotherapy on implants installed before the diagnosis of head and neck cancer, future research is also indicated in this regard.

Conclusion

Despite weak evidence on dental implant failure in irradiated patients, it is important to evaluate the survival of implants installed before and/or after radiotherapy in head and neck cancer patients, which may improve the quality of life for these patients. Radiotherapy was associated with a higher percentage of dental implant losses, especially for implants installed in the maxilla and grafted bone. There are many biases in the selected systematic reviews, and the strength of evidence for analysis is weak. Therefore, the results of this umbrella review are not reliable to provide security in clinical decision-making. New systematic reviews with high methodological quality are necessary to increase the power of evidence in the success of implant installation in irradiated patients with head and neck cancer.

Supporting information

Registration and Protocol

A detailed protocol was carried out before the beginning of this study and registered in the International Prospective Register of Systematic Reviews (PROSPERO) database under the identification number CRD42023406059.

Conflict of Interest

The authors have no competing interests.

References

1. Jung RE, Zembic A, Pjetursson BE, Zwahlen M, Thoma DS. Systematic review of the survival rate and the incidence of biological, technical, and aesthetic

complications of single crowns on implants reported in longitudinal studies with a mean follow-up of 5 years. Clin Oral Implants Res. 2012 Oct;23(6):2-21.

2. Papi P, Brauner E, Di Carlo S, Musio D, Tombolini M, De Angelis F, Valentini V, Tombolini V, Polimeni A, Pompa G. Crestal bone loss around dental implants placed in head and neck cancer patients treated with different radiotherapy techniques: a prospective cohort study. Int J Oral Maxillofac Surg: 2019 May;48(5):691-696; 2019.
3. Alberga JM, Korfage A, Bonnema I, Witjes MJH, Vissink A, Raghoebar GM. Mandibular dental implant placement immediately after teeth removal in head and neck cancer patients. Support Care Cancer: Dec;28(12):5911-5918; 2020.
4. Pieralli S, Spies BC, Schweppe F, Preissner S, Nelson K, Heiland M, Nahles S. Retrospective long-term clinical evaluation of implant-prosthetic rehabilitations after head and neck cancer therapy. Clin Oral Implants Res: Apr;32(12):470-486; 2021.
5. Li J, Feng K, Ye L, Liu Y, Sun Y, Wu Y. Influence of radiotherapy on dental implants placed in individuals before diagnosed with head and neck cancer: focus on implant-bed-specific radiation dosage. Clin Oral Investig: Sep;26(9):5915-5922; 2022.
6. Chrcanovic BR, Albrektsson T, Wennerberg A. Reasons for failures of oral implants. J Oral Rehabil: Jun;41(6):443-76; 2014.
7. Do TA, Le HS, Shen YW, Huang HL, Fuh LJ. Risk Factors related to Late Failure of Dental Implant-A Systematic Review of Recent Studies. Int J Environ Res Public Health: Jun 2;17(11):3931-3942; 2020.
8. Chrcanovic BR, Albrektsson T, Wennerberg A. Dental implants in irradiated versus nonirradiated patients: A meta-analysis. Head Neck: Mar;38(3):448-81; 2016.
9. Sung H, Ferlay J, Siegel RL, Laversanne M, Soerjomataram I, Jemal A, Bray F. Global Cancer Statistics 2020: GLOBOCAN Estimates of Incidence and Mortality Worldwide for 36 Cancers in 185 Countries. CA Cancer J Clin: May;71(3):209-249; 2021.
10. Schiegnitz E, Müller LK, Sagheb K, Theis L, Cagiran V, Kämmerer PW, Wegener J, Wagner W, Al-Nawas B. Clinical long-term and patient-reported outcomes of dental implants in oral cancer patients. Int J Implant Dent: Jul 13;7(1):209-249; 2021.
11. Schiegnitz E, Müller LK, Sagheb K, Theis L, Cagiran V, Kämmerer PW, Wegener J, Wagner W, Al-Nawas B. Changes in Peri-Implant Bone Level and Effect of Potential Influential Factors on Dental Implants in Irradiated and Nonirradiated Patients Following Multimodal Therapy Due to Head and Neck Cancer: A Retrospective Study. J Oral Maxillofac Surg: Oct;74(10):1965-73; 2016.
12. Neckel N, Wagendorf P, Sachse C, Stromberger C, Vach K, Heiland M, Nahles S. Influence of implant-specific radiation doses on peri-implant hard and soft tissue: An observational pilot study. Clin Oral Implants Res: Feb;32(2):249-261; 2021.
13. Wolf F, Spoerl S, Gottsauner M, Klingelhöffer C, Spanier G, Kolbeck C, Reichert TE, Hautmann MG, Ettl T. Significance of site-specific radiation dose and technique for success of implant-based prosthetic rehabilitation in irradiated head

and neck cancer patients-A cohort study. Clin Implant Dent Relat Res: Jun;23(3):444-455; 2021.

14. Delanian S, Lefaix JL. The radiation-induced fibroatrophic process: therapeutic perspective via the antioxidant pathway. Radiother Oncol: Nov;73(2):119-31; 2004.

15. Marx RE, Johnson RP. Studies in the radiobiology of osteoradionecrosis and their clinical significance. Oral Surg Oral Med Oral Pathol: Oct;64(4):379-90; 1987.

16. In 't Veld M, Schulten EAJM, Leusink FKJ. Immediate dental implant placement and restoration in the edentulous mandible in head and neck cancer patients: a systematic review and meta-analysis. Curr Opin Otolaryngol Head Neck Surg: Apr 1;29(2):126-137; 2021.

17. Schiegnitz E, Reinicke K, Sagheb K, König J, Al-Nawas B, Grötz KA. Dental implants in patients with head and neck cancer-A systematic review and meta-analysis of the influence of radiotherapy on implant survival. Clin Oral Implants Res: Oct;33(10):967-999; 2022.

18. Kende PP, Ranganath S, Landge JS, Sarda A, Wadewale M, Patil A, Singhavi HR. J Maxillofac Oral Surg. Clin Oral Implants Res: Sep;21(3):787-795; 2022.

19. Marques NP, Pérez-de-Oliveira ME, Normando AGC, Marques NCT, Epstein JB, Migliorati CA, Martelli-Júnior H, Ribeiro ACP, Rocha AC, Brandão TB, Sánchez FGV, Gueiros LAM, Lopes MA, Santos-Silva AR. Corrigendum to 'Clinical outcomes of dental implants in head and neck cancer patients: An overview'. Oral Surg Oral Med Oral Pathol Oral Radiol: Jul;136(1):42-53; 2023.

20. Zarzar AM, Sales PHDH, Barros AWP, Arreguy IMS, Carvalho AAT, Leão JC. Effectiveness of dental implants in patients undergoing radiotherapy for head and neck cancer: An umbrella review. Spec Care Dentist: Jan-Feb;44(1):40-56; 2024.

21. Page MJ, Moher D, Bossuyt PM, Boutron I, Hoffmann TC, Mulrow CD, Shamseer L, Tetzlaff JM, Akl EA, Brennan SE, Chou R, Glanville J, Grimshaw JM, Hróbjartsson A, Lalu MM, Li T, Loder EW, Mayo-Wilson E, McDonald S, McGuinness LA, Stewart LA, Thomas J, Tricco AC, Welch VA, Whiting P, McKenzie JE. PRISMA 2020 explanation and elaboration: updated guidance and exemplars for reporting systematic reviews. BMJ: Mar 29;372:n160; 2021.

22. Ouzzani M, Hammady H, Fedorowicz Z, Elmagarmid A. Rayyan-a web and mobile app for systematic reviews. Syst Rev: Dec 5;5(1):210; 2016.

23. Landis JR, Koch GG. The measurement of observer agreement for categorical data.. Biometrics: Mar;33(1):159-74; 1977.

24. Shea BJ, Reeves BC, Wells G, Thuku M, Hamel C, Moran J, Moher D, Tugwell P, Welch V, Kristjansson E, Henry DA. AMSTAR 2: a critical appraisal tool for systematic reviews that include randomised or non-randomised studies of healthcare interventions, or both. BMJ: Sep 21;358:j4008; 2017.

25. Benites Condezo AF, Araujo RZ, Koga DH, Curi MM, Cardoso CL. Hyperbaric oxygen therapy for the placement of dental implants in irradiated patients: systematic review and meta-analysis. Br J Oral Maxillofac Surg: Jul;59(6):625-632; 2021.

26. Schimmel M, Srinivasan M, McKenna G, Müller F. Effect of advanced age and/or systemic medical conditions on dental implant survival: A systematic review and meta-analysis. Clin Oral Implants Res: Oct;29 Suppl 16:311-330; 2018.

27. Ravi P, Vaishnavi D, Gnanam A, Krishnakumar Raja VB. The role of hyperbaric oxygen therapy in the prevention and management of radiation-induced complications of the head and neck - a systematic review of literature. J Stomatol Oral Maxillofac Surg: Dec;118(6):359-362; 2017.

28. Zhang L, Ding Q, Liu C, Sun Y, Xie Q, Zhou Y. Survival, Function, and Complications of Oral Implants Placed in Bone Flaps in Jaw Rehabilitation: A Systematic Review. Int J Prosthodont: Mar-Apr;29(2):115-25; 2016.

29. Shugaa-Addin B, Al-Shamiri HM, Al-Maweri S, Tarakji B. The effect of radiotherapy on survival of dental implants in head and neck cancer patients. J Clin Exp Dent: Apr 1;8(2):e194-200; 2016.

30. Zen Filho EV, Tolentino Ede S, Santos PS. Viability of dental implants in head and neck irradiated patients: A systematic review. Head Neck: Apr;38 Suppl 1:E2229-40; 2016.

31. Claudy MP, Miguens SA Jr, Celeste RK, Camara Parente R, Hernandez PA, da Silva AN Jr. Time interval after radiotherapy and dental implant failure: systematic review of observational studies and meta-analysis. Clin Implant Dent Relat Res: Apr;17(2):402-11; 2015.

32. Esposito M, Worthington HV. Interventions for replacing missing teeth: hyperbaric oxygen therapy for irradiated patients who require dental implants. Cochrane Database Syst Rev: Sep 30;2013(9):CD003603; 2013.

33. Nooh N. Dental implant survival in irradiated oral cancer patients: a systematic review of the literature. Int J Oral Maxillofac Implants: Sep-Oct;28(5):1233-42; 2013.

34. Esposito M, Grusovin MG, Patel S, Worthington HV, Coulthard P. Interventions for replacing missing teeth: hyperbaric oxygen therapy for irradiated patients who require dental implants. Cochrane Database Syst Rev: Jan 23;(1):CD003603; 2008.

35. Coulthard P, Esposito M, Worthington HV, Jokstad A. Interventions for replacing missing teeth: hyperbaric oxygen therapy for irradiated patients who require dental implants. Cochrane Database Syst Rev: Jan 23;(1):CD003603; 2002.

36. Koudougou C, Bertin H, Lecaplain B, Badran Z, Longis J, Corre P, Hoornaert A. Postimplantation radiation therapy in head and neck cancer patients: Literature review. Head Neck: Apr;42(4):794-802; 2020.

37. Thanya K, Ganapathy D. A review on dental implants in irradiated bones. Journal of Pharmaceutical Sciences and Research: Apr;8(7):635-637; 2016.

38. Tanaka TI, Chan HL, Tindle DI, Maceachern M, Oh TJ. Updated clinical considerations for dental implant therapy in irradiated head and neck cancer patients. J Prosthodont: Aug;22(6):432-8; 2013.

39. Dholam KP, Gurav SV. Dental implants in irradiated jaws: a literature review. J Cancer Res Ther: Jan;8 Suppl 1:S85-93; 2012.

40. Javed F, Al-Hezaimi K, Al-Rasheed A, Almas K, Romanos GE. Implant survival rate after oral cancer therapy: a review. Oral Oncol: Dec;46(12):854-9; 2010.

41. Schoen PJ, Reintsema H, Raghoebar GM, Vissink A, Roodenburg JL. The use of implant retained mandibular prostheses in the oral rehabilitation of head and neck cancer patients. A review and rationale for treatment planning. Oral Oncol: Oct;40(9):862-71; 2004.

42. Harrison JS, Stratemann S, Redding SW. Dental implants for patients who have had radiation treatment for head and neck cancer. Spec Care Dentist: Nov-Dec;23(6):223-9; 2003.

43. Marx RE, Morales MJ. The use of implants in the reconstruction of oral cancer patients. Dent Clin North Am: Jan;42(1):177-202; 1998.

44. Atanásio Pitorro TE, de Araújo Reis NT, Paranhos LR, Ferreira Soares PB. Survival of Dental Implants Placed Pre-Radiotherapy Versus Post-Radiotherapy in Native Bone: A Systematic Review. Int J Oral Maxillofac Implants: Nov-Dec;37(6):1100-1109; 2022.

45. Estafanous E, Osswald M, Ellingsen J-E, Oates TW, Chvartszaid D, Stanford CM. Dental implant success: systematic reviews and clinical outcomes. International Journal of oral and Maxillofacial Implants: Nov-Dec;37(2):231-234; 2022.

46. Recani B, Dulcic N, Catic A, Cimic S. Influence of systemic diseases on the dental implants success rate: Review article. Research Journal of Pharmaceutical, Biological and Chemical Sciences: Nov-Dec;5(4):1188-1193; 2014.

47. Garg A, Guez G. Head and neck cancer, dental implants, and dental oncology. Dent Implantol Update: Jan;22(1):1-8; 2011.

48. Hugentobler M, Guyot JP. Implants dentaires et radiothérapie [Dental implants and radiotherapy]. Rev Med Suisse: Sep 30;5(219):1936-9; 2009.

49. Colella G, Cannavale R, Pentenero M, Gandolfo S. Oral implants in radiated patients: a systematic review. Int J Oral Maxillofac Implants: Jul-Aug;22(4):616-22; 2007.

50. Samouhi P.; Buchbinder D. Rehabilitation of oral cancer patients with dental implants. Current Opinion in Otolaryngology and Head and Neck Surgery: Jul-Aug;8(4):305-313; 2000.

51. Lu B, Zhang X, Liu B. A systematic review and meta-analysis on influencing factors of failure of oral implant restoration treatment. Ann Palliat Med: Dec;10(12):12664-12677; 2021.

52. Kotsakis GA, Ioannou AL, Hinrichs JE, Romanos GE. A systematic review of observational studies evaluating implant placement in the maxillary jaws of medically compromised patients. Clin Implant Dent Relat Res: Jun;17(3):598-609; 2015.

53. Chen H, Liu N, Xu X, Qu X, Lu E. Smoking, radiotherapy, diabetes and osteoporosis as risk factors for dental implant failure: a meta-analysis. PLoS One: Aug 5;8(8):e71955; 2013.

54. Toneatti DJ, Graf RR, Burkhard JP, Schaller B. Survival of dental implants and occurrence of osteoradionecrosis in irradiated head and neck cancer patients: a systematic review and meta-analysis. Clin Oral Investig: Oct;25(10):5579-5593; 2021.

55. Panchal H, Shamsunder MG, Petrovic I, Rosen EB, Allen RJ Jr, Hernandez M, Ganly I, Boyle JO, Matros E, Nelson JA. Dental Implant Survival in Vascularized Bone Flaps: A Systematic Review and Meta-Analysis. Plast Reconstr Surg: Sep;146(3):637-648; 2020.

56. Korfage A, Dijkstra PU, Roodenburg JL, Reintsema H, Raghoebar GM, Vissink A. Dental implants in irradiated patients: which factors influence implant survival?. Clin Oral Investig: Sep;19(7):1689-90; 2015.

57. Schiegnitz E, Al-Nawas B, Kämmerer PW, Grötz KA. Dental implants in irradiated patients: which factors influence implant survival?. Clin Oral Investig: ep;19(7):1691-2; 2015.

58. Barber AJ, Butterworth CJ, Rogers SN. Systematic review of primary osseointegrated dental implants in head and neck oncology. Br J Oral Maxillofac Surg: Jan;49(1):29-36; 2011.

59. Camolesi GCV, Veronese HRM, Celestino MA, Blum DFC, Márquez-Zambrano JA, Carmona-Pérez FA, Jara-Venegas TA, Pellizzon ACA, Bernaola-Paredes WE. Survival of osseointegrated implants in head and neck cancer patients submitted to multimodal treatment: a systematic review and meta-analysis. Support Care Cancer: Oct 18;31(12):641; 2023.

60. Shahi A, Jebeli ZST, Yousefi H, Fazeli F, Fotovat F. Evaluation of the Success Rate of Dental Implants in Oral Cavity Cancers: A Systematic Review and Meta-analysis. Academic Journal of Health Sciences: Mar;38(2):54-59; 2023.

61. Shokouhi B, Cerajewska T. Radiotherapy and the survival of dental implants: a systematic review. Br J Oral Maxillofac Surg: May;60(4):422-429; 2022.

62. Gupta S, Mortellaro C, Panda S, Rovati M, Giacomello MS, Colletti L, Greco Lucchina A, Del Fabbro M. Dental implant survival rate in irradiated and non-radiated patients: a systematic review and meta-analysis. J Biol Regul Homeost Agents: Mar-Apr;35(2 Suppl. 1):53-65; 2021.

63. Smith Nobrega A, Santiago JF Jr, de Faria Almeida DA, Dos Santos DM, Pellizzer EP, Goiato MC. Irradiated patients and survival rate of dental implants: A systematic review and meta-analysis. J Prosthet Dent: Dec;116(6):858-866; 2016.

64. Chambrone L, Mandia J Jr, Shibli JA, Romito GA, Abrahao M. Dental implants installed in irradiated jaws: a systematic review. J Dent Res: Dec;92(12 Suppl):119S-30S; 2013.

65. Schiegnitz E, Al-Nawas B, Kämmerer PW, Grötz KA. Oral rehabilitation with dental implants in irradiated patients: a meta-analysis on implant survival. Clin Oral Investig: Apr;18(3):687-98; 2014.

66. Anderson L, Meraw S, Al-Hezaimi K, Wang HL. The influence of radiation therapy on dental implantology. Implant Dent: Feb;22(1):31-8; 2013.

67. Di Carlo S, De Angelis F, Ciolfi A, Quarato A, Piccoli L, Pompa G, Brauner E. Timing for implant placement in patients treated with radiotherapy of head and neck. Clin Ter: Sep-Oct;170(5):e345-e351; 2019.

68. Patel J, Antov H, Nixon P. Implant-supported oral rehabilitation in oncology patients: a retrospective cohort study. Br J Oral Maxillofac Surg: Oct;58(8):1003-1007; 2020.

Click here to access/download
Supporting Information
cor.art plosone2.pdf

Survival of dental implants in irradiated head and neck cancer patients compared to non-irradiated patients: An umbrella review

Maria Cândida Dourado Pacheco[1◑], Caio Fernando Teixeira Portela[1◑], Alexandre Henrique dos Reis-Prado[3◑], Lara Cancella de Arantes[2◑], Danilo Viegas da Costa[4◑], Tarcísio Passos Ribeiro Campos[1◑], Arno Heeren de Oliveira[1◑]

1 Department of Nuclear Engineering, Federal University of Minas Gerais, School of Engineering, Belo Horizonte, Minas Gerais, Brazil
2 Restorative Dentistry, Federal University of Minas Gerais, School of Dentistry, Belo Horizonte, Minas Gerais, Brazil
3 Restorative Dentistry, Federal University of Minas Gerais, School of Dentistry, Belo Horizonte, Minas Gerais, Brazil
4 Dental Clinics with an emphasis on Periodontics at the Pontifical Catholic University of Minas Gerais, Belo Horizonte, Minas Gerais, Brazil

◑These authors contributed equally to this work.
‡These authors also contributed equally to this work.
¤Current Address: Dept/Program/Center, Institution Name, City, State, Country
†Deceased
¶Membership list can be found in the Acknowledgments section.
*$caiofernando_fisica$@yahoo.com.br

Abstract

Background: There is an increasing demand for oral rehabilitation in patients undergoing irradiation for head and neck cancer treatment. Although radiotherapy appears to adversely affect implanted oral rehabilitation, the evidence provided by previous systematic reviews in the field remains controversial. Thus, this umbrella review aimed to evaluate the survival of dental implants installed before and/or after radiotherapy in patients with head and neck cancer compared to those not irradiated. **Methods:** A comprehensive search of electronic databases was performed in the Medline/PubMed, Cochrane Library, Embase, Web of Science, and Google Scholar databases, including manual searches, from inception to February, 2024, with no language or date restrictions. The Preferred Reporting Items for Systematic Reviews and Meta-Analyses (PRISMA) 2020 guidelines were followed. Survival percentage of dental implants was the primary outcome. A MeaSurement Tool to Assess systematic Reviews (AMSTAR) 2 was used as a critical appraisal tool for the included studies. The protocol for this review has been registered with PROSPERO (CRD42023406059). **Results:** Of the 1,811 articles screened, 11 articles that evaluated 73,674 implants were included. Quantitative analyses showed 2,674 failures out of 14,471 implants installed in irradiated bone and 1,825 failures out of 34,092 implants in non-irradiated bone. The survival rate was 81.52% for irradiated implants and 94.64% for non-irradiated implants. A significant difference in survival in favor of implants in non-irradiated bone is supported by 11 meta-analyses. The included systematic reviews showed critically low methodological quality. **Conclusions:** Despite the critically low methodological quality of the included studies, implants installed in irradiated patients had a higher percentage of failures. Further clinical studies employing improved research designs are warranted.

Author summary

Graduated in Dentistry from the Federal School of Dentistry of Diamantina (1993). PhD in Nuclear Sciences and Techniques from UFMG (2024), with a focus on radiation sciences. Master's degree in Dentistry, specializing in implantology, from PUC-Minas (2014). Specialist in Implantology (PUC-Minas/2014). Specialist in Periodontics from FAFEOD (2000). Currently works as an administrative technician and dentist at the Federal Institute of Northern Minas Gerais - Januária campus. Has experience in the field of Dentistry, with an emphasis on implantology, periodontics, and prosthetics.

Introduction

Osseointegrated dental implants are widely used and can provide a safe treatment option for edentulous patients, demonstrating promising survival rates within a 5 to 10-year follow-up period (Jung et al., 2012) [1]. However, the risks associated with implant failures have been extensively investigated over the years (Papi et al., 2019; Albrega et al., 2020; Pieralli et al., 2021; Li et al., 2022) [2, 3, 4, 5]. Possible reasons for failure are low insertion torque of implants planned to be loaded immediately or early, inexperienced surgeons inserting the implants, insertion of the implant into the maxilla, the patient history (radiotherapy, bruxism, periodontitis and early implant loss) (Chrcanovic et al., 2014; Do et al., 2020) [6, 7].

There is an increasing demand for oral rehabilitation in patients undergoing irradiation for head and neck cancer treatment (Chrcanovic et al., 2016) [8]. Cases of head and neck cancer are significantly increasing among the population (Sung et al., 2021) [9], which is also associated with the increased frequency of dental implants installed in these patients (Shiegnitz et al., 2021) [10]. However, radiotherapy seems to adversely affect implanted oral rehabilitation (Ernst et al., 2016; Neckel et al., 2021; Wolf et al., 2021) [11, 12, 13] due to radiation-induced fibroatrophic process (Delanian and Lefaix, 2004) [14], leading to tissue hypoxia, hypocellularity, hypovascularity and reduced bone regeneration capacity (Marx; Johnson, 1987) [15].

Although several systematic reviews have investigated the survival of dental implants in head and neck cancer patients undergoing radiotherapy involving various clinical analyses, the presented results have been inconclusive (In't Veld et al., 2021; Shiegnitz et al., 2022; Kende et al., 2022). [16, 17, 18] Recently evaluated in two umbrella reviews (Marques et al., 2023; Zarzar et al., 2024) [19, 20], oral rehabilitation in patients with irradiated head and neck cancer is a valid therapy. However, due to heterogeneity and the lack of a comparator group in most of the included systematic reviews, the recommendation is that the results presented be interpreted with caution. The present umbrella review aimed to analyse the available relevant systematic reviews, comparing irradiated versus non-irradiated patients, in an attempt to determine whether there is an influence of head and neck radiotherapy on dental implant survival in patients with head and neck cancer.

Materials and methods

This study followed the guidelines of the Preferred Reporting Items for Systematic Reviews and Meta-Analyses (PRISMA) 2020 (Page et al., 2021) [21]. The research protocol was registered on the International Prospective Register of Systematic Reviews (PROSPERO) database under the number CRD42023406059.

2.1 PICO formulation

The PICO (Population; Intervention; Comparison; Outcome) strategy was used to formulate the following research question: ”What is the survival of dental implants installed before and/or after radiotherapy in patients with head and neck cancer, compared to those not exposed to radiation?” (Table 1).

2.2 Eligibility Criteria

The inclusion criterion were systematic reviews, with or without meta-analysis, that assessed the survival of dental implants installed before and/or after radiotherapy in patients with head nd neck cancer, compared to patients with head and neck cancer in whom implants were installed in non-irradiated bone. The exclusion criteria were as follows: (1) systematic reviews of animal studies, (2) non-systematic reviews and editorials, (3) studies not focusing on the PICO question, and (4) studies with control groups not consisting non-irradiated individuals, and (3) studies for which the full text was unavailable. No restrictions were applied regarding the language or year of publication of the articles.

2.3 Research Sources and Search Strategy

The electronic search was conducted by two independent reviewers (MCDP and LCA), including studies published in PubMed/MEDLINE, Cochrane Library, Embase, and Web of Science, from their inception until February 23, 2024. Additionally, grey literature search was searched in Google Scholar database. Search strategies were developed for each database using indexed terms and synonyms (Supporting Information S1). A manual search of references was performed in all included studies. In case of doubt, a third reviewer (DVC) was consulted. There were no restrictions on language or year of publication for the articles.

2.4 Study Selection Process

The selection of studies was conducted by two reviewers (MCDP and LCA) independently, in a two-stage process using the Ryyan app (Qatar Foundation, Qatar) (Ouzzani et al., 2016) [22]. Duplicate studies were excluded using reference management software (EndNote® X9; Clarivate, Philadelphia, PA, USA). In the first stage, titles and abstracts were assessed, and in the next stage, the full text selected from the titles/abstracts was read, and those that did not meet the eligibility criteria were excluded. Disagreements were resolved by a third reviewer (DVC). Contacting authors to clarify doubts was not necessary. The level of agreement between the reviewers was assessed by Cohen's Kappa (Landis and Koch, 1977) [23].

2.5 Data Extraction and Analysis

Data were collected by two reviewers (MCDP and LCA) independently in April 2024, based on the main characteristics of the studies, in an Excel form created and customized for the current review and included: study and publication year, number of participants/age range, country of publication, number of included studies/study design, presence of meta-analysis, variation in radiation dose (Gy)/implant installation time, number of failed/installed implants, implantation site, time interval between radiotherapy and implant installation, follow-up period, implant characteristics, cancer characteristics, adjuvant therapy, bone crest stability, postoperative complications, prosthetic rehabilitation. Subsequently, a third author (DVC) reviewed the data. In cases of missing data, authors would be contacted by email twice.

2.6 Assessment of Methodological Quality

The quality of included systematic reviews was appraised by using the A MeaSurement Tool to Assess Systematic Reviews (AMSTAR 2) tool (Shea et al., 2017) [24], and it was independently assessed by two reviewers (MCDP and LCA). Disagreements were resolved by a third reviewer (DVC). The methodological quality of systematic reviews was assessed using the, which consists of 16 items, where items 2, 4, 7, 9, 11, 13, and 15 are considered critical. Questions are answered with options: yes, partial yes, no, or meta-analysis not conducted. The overall confidence rating of the review's results is categorized as high (none or one non-critical weakness), moderate (more than one non-critical weakness), low (one critical flaw with or without non-critical weaknesses), or critically low (more than one critical flaw with or without non-critical weaknesses).

2.7 Results Synthesis

A descriptive synthesis of the results of the included studies was conducted. If a study did not provide raw data on implant failure but presented percentages related to implant survival, the data were converted when necessary. The percentage of implant survival was calculated by arithmetic mean.

Results

3.1 Study Selection

The PRISMA flowchart depicting the study selection process is presented in Figure 1. The literature search strategy identified 1,811 articles. After removing 670 duplicates, the remaining 1,141 publications were evaluated by reading titles/abstracts. A total of five additional reports were identified through manual search. This resulted in 45 records selected for reading the full text. Thirty-four references were excluded because 11 did not investigate the control group - non-irradiated (Benites Condezo et al., 2021; Schimmel et al., 2018; Ravi et al., 2017; Zhang et al., 2016; Shugaa Addin et al., 2016; Zen Filho et al., 2016; Claudy et al., 2015; Esposito and Worthington, 2013; Nooh, 2013; Esposito et al., 2008; Coulthard et al., 2002) [25, 26, 27, 28, 29, 30, 31, 32, 33, 34, 35], eight were not systematic reviews (Koudougou et al., 2020; Thanya and Ganapathy, 2016; Tanaka et al., 2013; Dholam and Gurav, 2012; Javed et al., 2010; Schoen et al., 2004; Harrison et al., 2003; Marx and Morales, 1998) [36, 37, 38, 39, 40, 41, 42, 43], in 7 references the full text was unavailable (Atanásio Pitorro et al., 2022; Estafanous et al., 2022; Recani et al., 2014; Garg and Guez, 2011; Hugentobl and Guyot, 2009; Colella et al., 2007; Samouhi and Buchbinder, 2000) [44, 45, 46, 47, 48, 49, 50], three did not focus on oral rehabilitation with implants in irradiated patients (Lu et al., 2021; Kotsakis et al., 2015; Chen et al., 2013) [51, 52, 53], two included nonspecific data for head and neck cancer (Toneatti et al., 2021; Panchal et al., 2020) [54, 55]; two were editorials (Korfage et al., 2015; Schiegnitz et al., 2015) [56, 57], and one did not investigate implant survival (Barber et al., 2011) [58]. Finally, eleven systematic reviews were included for qualitative synthesis. A table of all studies identified in the literature search, including those that were excluded from the analyses with the reasons for exclusion is presented in Supporting Information S2. The inter-rater kappa coefficients indicated an almost perfect agreement level among reviewers during study selection, with values greater than 0.800 for the databases.

3.2 Main characteristics of included reviews

Tables 2 and 3 show the characteristics of the eligible systematic reviews. A total of 16,193 patients who received 73,674 dental implants were included. Of these, approximately 31,137 were installed in irradiated sites. All selected studies (Camolesi et al., 2023; Shahi et al., 2023; Kende et al., 2022; Schiegnitz et al., 2022; Shokouhi and Cerajewska, 2022; Gupta et al., 2021; In't Veld et al., 2021; Smith Nobrega et al., 2016; Chrcanovic et al., 2016; Chambrone et al., 2013) [65, 66, 24, 23, 67, 68, 22, 69, 14, 70] assessed the survival of dental implants installed after the diagnosis of head and neck cancer. One systematic review (Shiegnitz et al., 2022) [23] was an update of a previous study elaborated by the same research team (Shiegnitz et al., 2014) [71]. The age range of participants varied from 6 to 94 years, with one study not reporting the sample age (Chambrone et al., 2013) [70]. Among the included reviews, most evaluated data from retrospective studies. All included studies conducted meta-analysis.

The minimum radiation dose among the studies ranged from 10 Gy to 40 Gy and the maximum dose from 72 Gy to 145 Gy; two studies did not mention the radiation dose (Shahi et al., 2023; Chambrone et al., 2013) [66, 70]. Regarding the timing of implant installation, five articles described implants installed before and after radiotherapy (Kende et al., 2022; Schiegnitz et al., 2022; Smith Nobrega et al., 2016; Chrcanovic et al., 2016; Chambrone et al., 2013) [24, 23, 69, 14, 70], four reported implants installed after radiotherapy (Camolesi et al., 2023; Shahi et al., 2023; Shokouhi and Cerajewska, 2022; Gupta et al., 2021) [65, 66, 67, 68]; one study evaluated implant installation during panendoscopy or ablative surgery - before radiotherapy (In't Veld et al., 2021) [22]. All selected studies evaluated the survival of implants installed in the maxilla and mandible. While seven studies investigated implants installed in native and grafted bone (Camolesi et al., 2023; Kende et al., 2022; Schiegnitz et al., 2022; Gupta et al., 2021; In't Veld et al., 2021; Smith Nobrega et al., 2016; Chrcanovic et al., 2016) [65, 24, 23, 68, 22, 69, 14], two analyzed implants installed only in native bone (Shokouhi and Cerajewska, 2022; Chambrone et al., 2013) [67, 70], and one study did not mention (Shahi et al., 2023) [66]. Only one study investigated the time interval between radiotherapy and implantation (Chrcanovic et al., 2016) [4]. The follow-up time among the studies varied from one month to 23 years, with one study not mentioning the follow-up period (Shahi et al., 2023) [66]. More details regarding the study design, number of implants installed, number of failed implants, and implant survival percentage extracted from the primary articles are described in Supporting Information S3.

3.3 Synthesis of results

Survival percentage of dental implants in irradiated and non-irradiated patients. The survival rate of implants was higher among non-irradiated patients according to data from 11 meta-analyses. Conversely, three meta-analyses showed no significant difference between the irradiated and non-irradiated groups (Kende et al., 2022; In't Veld et al., 2021; Shiegnitz et al., 2014 – literature between 2007-2013). Data were reported in four time periods (1990-2021, 1990-2006, 2007-2013, and 2013-2021) [18, 16, 65] and two follow-up periods ($\geq$ 3 years of follow-up and $\geq$ 5 years of follow-up). The effect estimate and precision of each meta-analysis are presented in Table 4. Further information on the number of failed/installed implants, number of primary articles included in the meta-analysis, and the percentage of survival is also shown in Table 4. Overall, 48,563 implants were evaluated, including 14,471 with 2,674 failures in the irradiated group and 34,092 with 1,825 failures in the non-irradiated group. The survival percentage was 81.52% and 94.64%, respectively.

Survival percentage of Implants Installed in Irradiated Maxilla versus Irradiated Mandible

Two meta-analyses (Chrcanovic et al., 2016; Chambrone et al., 2013) [8, 64] showed higher implant survival installed in irradiated mandible compared to irradiated maxilla (RR: 3.16; 95% CI: 1.76-5.68; p=0.0001; I2=70%) (RR: 5.96; 95% CI: 2.71-13.12; p¡0.00001; I2=33%). A total of 3,378 implants were evaluated, including 897 with 188 failures in the irradiated maxilla group and 2,481 with 160 failures in the irradiated mandible group. The survival percentage was 79.04% and 93.55%, respectively.

In the study by Shokouhi and Cerajewska (2022) [61], implants installed in the maxilla were significantly more likely to fail than those installed in the mandible (OR: 5.03; 95% CI: 1.07-23.58; p=0.04; I2=87%). Survival of Implants Installed in Irradiated Grafted Bone versus Irradiated Native Bone Two meta-analyses (Shiegnitz et al., 2022 – literature 1990-2021) showed higher implant survival in irradiated native bone [17]. (OR: 2.06; 95% CI: 1.44-2.94; p¡0.0001; I2=0% - follow-up $\geq$ 36 months) (OR: 2.26; 95% CI: 1.50-3.40; p¡0.0001; I2=12% - follow-up $\geq$ 60 months).

Conversely, one meta-analysis (Chrcanovic et al., 2016) [8] showed no significant difference between irradiated grafted bone and irradiated native bone (RR: 1.35; 95% CI: 0.93-1.94; p=0.11; I2=48%). A total of 1,740 implants were evaluated, including 596 with 106 failures in the irradiated grafted bone group and 1,144 with 148 failures in the irradiated native bone group. The survival percentage was 82.21% and 87.06%, respectively.

Regarding the time interval between the end of radiotherapy and implant installation (up to 12 months versus after 12 months), and the evaluation of radiation dose (high doses versus low doses), there was no influence of these variables on implant failure among the evaluated groups (Chrcanovic et al., 2016) [8].

3.4 Methodological Quality of Systematic Reviews

Overall, according to the evaluated domains using the AMSTAR 2 tool, the eligible reviews exhibited critically low quality (Table 5). The justifications for downgrading the studies are detailed in the Supporting Information S4.

Discussion

The present umbrella review included eleven systematic reviews published between 2013 and 2023, assessing the survival of 14,471 dental implants installed before and/or after radiotherapy in head and neck cancer patients, compared to 34,092 implants in non-irradiated patients. Overall, the survival rate of implants installed in irradiated patients was considered inferior compared to those installed in non-irradiated patients. Implant survival in the irradiated maxilla was significantly lower compared to the irradiated mandible. Dental implants installed in irradiated grafted bone were considered more prone to failure compared to irradiated native bone. The methodological quality of the selected studies was considered critically low.

Evidence from systematic reviews supports that implant survival is influenced by the effect of radiotherapy (Panchal et al., 2020; Toneatti et al., 2021) [55, 54]. Zarzar et al., (2024) [20], in an umbrella review, demonstrated that the implant survival rate in irradiated patients was lower compared to non-irradiated patients, with follow-up ranging from 1 month to 23 years. The present umbrella review evaluated and summarized scientific evidence available in the literature, corroborating previous reviews. It is important to mention that the analyzed data had different follow-up periods, which could cause significant errors in the descriptive synthesis presented in this study.

The impact of radiation dose on the survival of dental implants installed in head and neck cancer patients depends on the specific dose at the implant site (¿50 Gy) and the parotid gland (¿30 Gy) (Wolf et al., 2021) [13]. An umbrella review reported that radiation doses lower than 50 Gy were associated with better survival rates (Marques et al., 2023) [19]. This is consistent with the results observed in this umbrella review. It is important to consider that these data compare high doses with low doses, not detailing the implantation site with the target volume of radiation. Moreover, the systematic reviews included studies that varied the maximum doses from 72 Gy to 145 Gy, which may represent a significant risk of implant failure and osteoradionecrosis (Anderson et al., 2013) [66].

Most implants evaluated in this review were installed after the completion of radiotherapy. Implants installed after the end of radiotherapy were significantly more likely to fail than those installed in non-irradiated bone (Gupta et al., 2021; Shokouhi and Cerajewka, 2021) [62, 61]. Whereas implants installed immediately during ablative surgery (pre-RT) showed no statistically significant difference between irradiated and non-irradiated groups (In't Veld et al., 2021) [16]. There is a trend to install the implant during tumor removal, significantly reducing prosthetic rehabilitation time and, on the other hand, increasing complications that may hinder implantation success, such as obtaining a well-positioned implant (Di Carlo et al., 2019; Patel et al., 2020; Albrega et al., 2020) [67, 68, 3].

The time interval between radiotherapy and implant surgery may influence implant survival. A meta-analysis showed no statistically significant difference when comparing implant failures installed before and after 12 months of radiotherapy (Chrcanovic et al., 2016) [8]. Claudy et al. (2013) [31], in a meta-analysis, evaluated the time interval between 6 and 12 months after radiotherapy versus 12 months after radiotherapy, and an increased risk of 34% was observed for implants installed between 6 and 12 months post-radiotherapy. The literature recommends implant installation at least six months after the completion of radiotherapy, the expected period for the end of the acute process in bone and soft tissues induced by radiation (Marx and Johnson, 1987) [15].

The umbrella reviews, Zarzar et al. (2024) [20] and Marques et al. (2023) [19], using the AMSTAR 2 tool, included five systematic reviews in common with this study (Chambrone et al., 2013; Shiegnitz et al., 2014; Chrcanovic et al., 2016; Smith Nobrega et al., 2016; In't Veld et al., 2021) [64, 65, 8, 63, 16]. Zarzar et al. (2024) [20] classified the systematic reviews of Shiegnitz et al. (2014) [65], Smith Nobrega et al. (2016) [63], and In't Veld et al. (2021) [16] as critically low, which is in line with the results observed in this study. In contrast, the systematic review by Chambrone et al. (2013) [64] was considered high quality and Chrcanovic et al. (2016) [8] as low quality. This difference could be explained by the complexity associated with judging the issues. Marques et al. (2023) [19] analyses combined responses to the 16 individual items and performed an overall score to assess the risk of bias of the studies, making the classification criteria different from the assessments of this study.

The present study investigated irradiated head and neck cancer patients, recommending caution in interpreting the results, despite the high survival rate demonstrated for dental implants. The evaluation of methodological quality, through the AMSTAR 2 tool, showed that although the included studies reported the use of the PRISMA guideline, only two systematic reviews reported prior registration of the research protocol (Camolesi et al., 2023; Kende et al., 2022) [59, 18]. The PRISMA guideline itself advises indicating where the review protocol can be accessed to allow readers to assess whether any deviations from the protocol compared to what was done may have introduced bias. Some limitations should be considered when interpreting the results of this umbrella review. The search strategy identified only eleven systematic reviews, all classified with critically low methodological quality. Considering the

retrospective nature of most primary studies included in the selected systematic reviews, it was observed that the reported data were notably heterogeneous, especially for questions related to implant characteristics, radiation, and follow-up period. The effect measure used in conducting the selected systematic reviews was the number of implants that failed and the number of implants installed, not including details about the patient or radiation to answer the research question. Additionally, implant surface properties are confounding factors to be considered. In this sense, some studies (Chambrone et al., 2013; Chrcanovic et al., 2016; Smith Nobrega et al., 2016; In't Veld et al., 2021; Gupta et al., 2021) [64, 8, 63, 16, 62] sought to evaluate the geometry and surface treatment of the implant. However, it was not possible to reach a consensus on the risk of implant failures based on their characteristics because most individual studies did not report these data.

The main finding of this study was that, for patients after the diagnosis of head and neck cancer, the survival of implants that were irradiated before and/or after implantation was lower than that of implants installed in non-irradiated patients. We recommend that future randomized controlled trials be conducted on the topic. On the other hand, we consider the difficulty involved in conducting controlled and randomized experiments in this population. Therefore, new prospective and retrospective observational studies are needed. The literature is underexplored regarding the influence of radiotherapy on implants installed before the diagnosis of head and neck cancer, future research is also indicated in this regard.

Conclusion

Despite weak evidence on dental implant failure in irradiated patients, it is important to evaluate the survival of implants installed before and/or after radiotherapy in head and neck cancer patients, which may improve the quality of life for these patients. Radiotherapy was associated with a higher percentage of dental implant losses, especially for implants installed in the maxilla and grafted bone. There are many biases in the selected systematic reviews, and the strength of evidence for analysis is weak. Therefore, the results of this umbrella review are not reliable to provide security in clinical decision-making. New systematic reviews with high methodological quality are necessary to increase the power of evidence in the success of implant installation in irradiated patients with head and neck cancer.

Supporting information

Registration and Protocol

A detailed protocol was carried out before the beginning of this study and registered in the International Prospective Register of Systematic Reviews (PROSPERO) database under the identification number CRD42023406059.

Conflict of Interest

The authors have no competing interests.

References

1. Jung RE, Zembic A, Pjetursson BE, Zwahlen M, Thoma DS. Systematic review of the survival rate and the incidence of biological, technical, and aesthetic

complications of single crowns on implants reported in longitudinal studies with a mean follow-up of 5 years. Clin Oral Implants Res. 2012 Oct;23(6):2-21.

2. Papi P, Brauner E, Di Carlo S, Musio D, Tombolini M, De Angelis F, Valentini V, Tombolini V, Polimeni A, Pompa G. Crestal bone loss around dental implants placed in head and neck cancer patients treated with different radiotherapy techniques: a prospective cohort study. Int J Oral Maxillofac Surg: 2019 May;48(5):691-696; 2019.

3. Alberga JM, Korfage A, Bonnema I, Witjes MJH, Vissink A, Raghoebar GM. Mandibular dental implant placement immediately after teeth removal in head and neck cancer patients. Support Care Cancer: Dec;28(12):5911-5918; 2020.

4. Pieralli S, Spies BC, Schweppe F, Preissner S, Nelson K, Heiland M, Nahles S. Retrospective long-term clinical evaluation of implant-prosthetic rehabilitations after head and neck cancer therapy. Clin Oral Implants Res: Apr;32(12):470-486; 2021.

5. Li J, Feng K, Ye L, Liu Y, Sun Y, Wu Y. Influence of radiotherapy on dental implants placed in individuals before diagnosed with head and neck cancer: focus on implant-bed-specific radiation dosage. Clin Oral Investig: Sep;26(9):5915-5922; 2022.

6. Chrcanovic BR, Albrektsson T, Wennerberg A. Reasons for failures of oral implants. J Oral Rehabil: Jun;41(6):443-76; 2014.

7. Do TA, Le HS, Shen YW, Huang HL, Fuh LJ. Risk Factors related to Late Failure of Dental Implant-A Systematic Review of Recent Studies. Int J Environ Res Public Health: Jun 2;17(11):3931-3942; 2020.

8. Chrcanovic BR, Albrektsson T, Wennerberg A. Dental implants in irradiated versus nonirradiated patients: A meta-analysis. Head Neck: Mar;38(3):448-81; 2016.

9. Sung H, Ferlay J, Siegel RL, Laversanne M, Soerjomataram I, Jemal A, Bray F. Global Cancer Statistics 2020: GLOBOCAN Estimates of Incidence and Mortality Worldwide for 36 Cancers in 185 Countries. CA Cancer J Clin: May;71(3):209-249; 2021.

10. Schiegnitz E, Müller LK, Sagheb K, Theis L, Cagiran V, Kämmerer PW, Wegener J, Wagner W, Al-Nawas B. Clinical long-term and patient-reported outcomes of dental implants in oral cancer patients. Int J Implant Dent: Jul 13;7(1):209-249; 2021.

11. Schiegnitz E, Müller LK, Sagheb K, Theis L, Cagiran V, Kämmerer PW, Wegener J, Wagner W, Al-Nawas B. Changes in Peri-Implant Bone Level and Effect of Potential Influential Factors on Dental Implants in Irradiated and Nonirradiated Patients Following Multimodal Therapy Due to Head and Neck Cancer: A Retrospective Study. J Oral Maxillofac Surg: Oct;74(10):1965-73; 2016.

12. Neckel N, Wagendorf P, Sachse C, Stromberger C, Vach K, Heiland M, Nahles S. Influence of implant-specific radiation doses on peri-implant hard and soft tissue: An observational pilot study. Clin Oral Implants Res: Feb;32(2):249-261; 2021.

13. Wolf F, Spoerl S, Gottsauner M, Klingelhöffer C, Spanier G, Kolbeck C, Reichert TE, Hautmann MG, Ettl T. Significance of site-specific radiation dose and technique for success of implant-based prosthetic rehabilitation in irradiated head

and neck cancer patients-A cohort study. Clin Implant Dent Relat Res: Jun;23(3):444-455; 2021.

14. Delanian S, Lefaix JL. The radiation-induced fibroatrophic process: therapeutic perspective via the antioxidant pathway. Radiother Oncol: Nov;73(2):119-31; 2004.
15. Marx RE, Johnson RP. Studies in the radiobiology of osteoradionecrosis and their clinical significance. Oral Surg Oral Med Oral Pathol: Oct;64(4):379-90; 1987.
16. In 't Veld M, Schulten EAJM, Leusink FKJ. Immediate dental implant placement and restoration in the edentulous mandible in head and neck cancer patients: a systematic review and meta-analysis. Curr Opin Otolaryngol Head Neck Surg: Apr 1;29(2):126-137; 2021.
17. Schiegnitz E, Reinicke K, Sagheb K, König J, Al-Nawas B, Grötz KA. Dental implants in patients with head and neck cancer-A systematic review and meta-analysis of the influence of radiotherapy on implant survival. Clin Oral Implants Res: Oct;33(10):967-999; 2022.
18. Kende PP, Ranganath S, Landge JS, Sarda A, Wadewale M, Patil A, Singhavi HR. J Maxillofac Oral Surg. Clin Oral Implants Res: Sep;21(3):787-795; 2022.
19. Marques NP, Pérez-de-Oliveira ME, Normando AGC, Marques NCT, Epstein JB, Migliorati CA, Martelli-Júnior H, Ribeiro ACP, Rocha AC, Brandão TB, Sánchez FGV, Gueiros LAM, Lopes MA, Santos-Silva AR. Corrigendum to 'Clinical outcomes of dental implants in head and neck cancer patients: An overview'. Oral Surg Oral Med Oral Pathol Oral Radiol: Jul;136(1):42-53; 2023.
20. Zarzar AM, Sales PHDH, Barros AWP, Arreguy IMS, Carvalho AAT, Leão JC. Effectiveness of dental implants in patients undergoing radiotherapy for head and neck cancer: An umbrella review. Spec Care Dentist: Jan-Feb;44(1):40-56; 2024.
21. Page MJ, Moher D, Bossuyt PM, Boutron I, Hoffmann TC, Mulrow CD, Shamseer L, Tetzlaff JM, Akl EA, Brennan SE, Chou R, Glanville J, Grimshaw JM, Hróbjartsson A, Lalu MM, Li T, Loder EW, Mayo-Wilson E, McDonald S, McGuinness LA, Stewart LA, Thomas J, Tricco AC, Welch VA, Whiting P, McKenzie JE. PRISMA 2020 explanation and elaboration: updated guidance and exemplars for reporting systematic reviews. BMJ: Mar 29;372:n160; 2021.
22. Ouzzani M, Hammady H, Fedorowicz Z, Elmagarmid A. Rayyan-a web and mobile app for systematic reviews. Syst Rev: Dec 5;5(1):210; 2016.
23. Landis JR, Koch GG. The measurement of observer agreement for categorical data.. Biometrics: Mar;33(1):159-74; 1977.
24. Shea BJ, Reeves BC, Wells G, Thuku M, Hamel C, Moran J, Moher D, Tugwell P, Welch V, Kristjansson E, Henry DA. AMSTAR 2: a critical appraisal tool for systematic reviews that include randomised or non-randomised studies of healthcare interventions, or both. BMJ: Sep 21;358:j4008; 2017.
25. Benites Condezo AF, Araujo RZ, Koga DH, Curi MM, Cardoso CL. Hyperbaric oxygen therapy for the placement of dental implants in irradiated patients: systematic review and meta-analysis. Br J Oral Maxillofac Surg: Jul;59(6):625-632; 2021.

26. Schimmel M, Srinivasan M, McKenna G, Müller F. Effect of advanced age and/or systemic medical conditions on dental implant survival: A systematic review and meta-analysis. Clin Oral Implants Res: Oct;29 Suppl 16:311-330; 2018.

27. Ravi P, Vaishnavi D, Gnanam A, Krishnakumar Raja VB. The role of hyperbaric oxygen therapy in the prevention and management of radiation-induced complications of the head and neck - a systematic review of literature. J Stomatol Oral Maxillofac Surg: Dec;118(6):359-362; 2017.

28. Zhang L, Ding Q, Liu C, Sun Y, Xie Q, Zhou Y. Survival, Function, and Complications of Oral Implants Placed in Bone Flaps in Jaw Rehabilitation: A Systematic Review. Int J Prosthodont: Mar-Apr;29(2):115-25; 2016.

29. Shugaa-Addin B, Al-Shamiri HM, Al-Maweri S, Tarakji B. The effect of radiotherapy on survival of dental implants in head and neck cancer patients. J Clin Exp Dent: Apr 1;8(2):e194-200; 2016.

30. Zen Filho EV, Tolentino Ede S, Santos PS. Viability of dental implants in head and neck irradiated patients: A systematic review. Head Neck: Apr;38 Suppl 1:E2229-40; 2016.

31. Claudy MP, Miguens SA Jr, Celeste RK, Camara Parente R, Hernandez PA, da Silva AN Jr. Time interval after radiotherapy and dental implant failure: systematic review of observational studies and meta-analysis. Clin Implant Dent Relat Res: Apr;17(2):402-11; 2015.

32. Esposito M, Worthington HV. Interventions for replacing missing teeth: hyperbaric oxygen therapy for irradiated patients who require dental implants. Cochrane Database Syst Rev: Sep 30;2013(9):CD003603; 2013.

33. Nooh N. Dental implant survival in irradiated oral cancer patients: a systematic review of the literature. Int J Oral Maxillofac Implants: Sep-Oct;28(5):1233-42; 2013.

34. Esposito M, Grusovin MG, Patel S, Worthington HV, Coulthard P. Interventions for replacing missing teeth: hyperbaric oxygen therapy for irradiated patients who require dental implants. Cochrane Database Syst Rev: Jan 23;(1):CD003603; 2008.

35. Coulthard P, Esposito M, Worthington HV, Jokstad A. Interventions for replacing missing teeth: hyperbaric oxygen therapy for irradiated patients who require dental implants. Cochrane Database Syst Rev: Jan 23;(1):CD003603; 2002.

36. Koudougou C, Bertin H, Lecaplain B, Badran Z, Longis J, Corre P, Hoornaert A. Postimplantation radiation therapy in head and neck cancer patients: Literature review. Head Neck: Apr;42(4):794-802; 2020.

37. Thanya K, Ganapathy D. A review on dental implants in irradiated bones. Journal of Pharmaceutical Sciences and Research: Apr;8(7):635-637; 2016.

38. Tanaka TI, Chan HL, Tindle DI, Maceachern M, Oh TJ. Updated clinical considerations for dental implant therapy in irradiated head and neck cancer patients. J Prosthodont: Aug;22(6):432-8; 2013.

39. Dholam KP, Gurav SV. Dental implants in irradiated jaws: a literature review. J Cancer Res Ther: Jan;8 Suppl 1:S85-93; 2012.

40. Javed F, Al-Hezaimi K, Al-Rasheed A, Almas K, Romanos GE. Implant survival rate after oral cancer therapy: a review. Oral Oncol: Dec;46(12):854-9; 2010.

41. Schoen PJ, Reintsema H, Raghoebar GM, Vissink A, Roodenburg JL. The use of implant retained mandibular prostheses in the oral rehabilitation of head and neck cancer patients. A review and rationale for treatment planning. Oral Oncol: Oct;40(9):862-71; 2004.

42. Harrison JS, Stratemann S, Redding SW. Dental implants for patients who have had radiation treatment for head and neck cancer. Spec Care Dentist: Nov-Dec;23(6):223-9; 2003.

43. Marx RE, Morales MJ. The use of implants in the reconstruction of oral cancer patients. Dent Clin North Am: Jan;42(1):177-202; 1998.

44. Atanásio Pitorro TE, de Araújo Reis NT, Paranhos LR, Ferreira Soares PB. Survival of Dental Implants Placed Pre-Radiotherapy Versus Post-Radiotherapy in Native Bone: A Systematic Review. Int J Oral Maxillofac Implants: Nov-Dec;37(6):1100-1109; 2022.

45. Estafanous E, Osswald M, Ellingsen J-E, Oates TW, Chvartszaid D, Stanford CM. Dental implant success: systematic reviews and clinical outcomes. International Journal of oral and Maxillofacial Implants: Nov-Dec;37(2):231-234; 2022.

46. Recani B, Dulcic N, Catic A, Cimic S. Influence of systemic diseases on the dental implants success rate: Review article. Research Journal of Pharmaceutical, Biological and Chemical Sciences: Nov-Dec;5(4):1188-1193; 2014.

47. Garg A, Guez G. Head and neck cancer, dental implants, and dental oncology. Dent Implantol Update: Jan;22(1):1-8; 2011.

48. Hugentobler M, Guyot JP. Implants dentaires et radiothérapie [Dental implants and radiotherapy]. Rev Med Suisse: Sep 30;5(219):1936-9; 2009.

49. Colella G, Cannavale R, Pentenero M, Gandolfo S. Oral implants in radiated patients: a systematic review. Int J Oral Maxillofac Implants: Jul-Aug;22(4):616-22; 2007.

50. Samouhi P.; Buchbinder D. Rehabilitation of oral cancer patients with dental implants. Current Opinion in Otolaryngology and Head and Neck Surgery: Jul-Aug;8(4):305-313; 2000.

51. Lu B, Zhang X, Liu B. A systematic review and meta-analysis on influencing factors of failure of oral implant restoration treatment. Ann Palliat Med: Dec;10(12):12664-12677; 2021.

52. Kotsakis GA, Ioannou AL, Hinrichs JE, Romanos GE. A systematic review of observational studies evaluating implant placement in the maxillary jaws of medically compromised patients. Clin Implant Dent Relat Res: Jun;17(3):598-609; 2015.

53. Chen H, Liu N, Xu X, Qu X, Lu E. Smoking, radiotherapy, diabetes and osteoporosis as risk factors for dental implant failure: a meta-analysis. PLoS One: Aug 5;8(8):e71955; 2013.

54. Toneatti DJ, Graf RR, Burkhard JP, Schaller B. Survival of dental implants and occurrence of osteoradionecrosis in irradiated head and neck cancer patients: a systematic review and meta-analysis. Clin Oral Investig: Oct;25(10):5579-5593; 2021.

55. Panchal H, Shamsunder MG, Petrovic I, Rosen EB, Allen RJ Jr, Hernandez M, Ganly I, Boyle JO, Matros E, Nelson JA. Dental Implant Survival in Vascularized Bone Flaps: A Systematic Review and Meta-Analysis. Plast Reconstr Surg: Sep;146(3):637-648; 2020.

56. Korfage A, Dijkstra PU, Roodenburg JL, Reintsema H, Raghoebar GM, Vissink A. Dental implants in irradiated patients: which factors influence implant survival?. Clin Oral Investig: Sep;19(7):1689-90; 2015.

57. Schiegnitz E, Al-Nawas B, Kämmerer PW, Grötz KA. Dental implants in irradiated patients: which factors influence implant survival?. Clin Oral Investig: ep;19(7):1691-2; 2015.

58. Barber AJ, Butterworth CJ, Rogers SN. Systematic review of primary osseointegrated dental implants in head and neck oncology. Br J Oral Maxillofac Surg: Jan;49(1):29-36; 2011.

59. Camolesi GCV, Veronese HRM, Celestino MA, Blum DFC, Márquez-Zambrano JA, Carmona-Pérez FA, Jara-Venegas TA, Pellizzon ACA, Bernaola-Paredes WE. Survival of osseointegrated implants in head and neck cancer patients submitted to multimodal treatment: a systematic review and meta-analysis. Support Care Cancer: Oct 18;31(12):641; 2023.

60. Shahi A, Jebeli ZST, Yousefi H, Fazeli F, Fotovat F. Evaluation of the Success Rate of Dental Implants in Oral Cavity Cancers: A Systematic Review and Meta-analysis. Academic Journal of Health Sciences: Mar;38(2):54-59; 2023.

61. Shokouhi B, Cerajewska T. Radiotherapy and the survival of dental implants: a systematic review. Br J Oral Maxillofac Surg: May;60(4):422-429; 2022.

62. Gupta S, Mortellaro C, Panda S, Rovati M, Giacomello MS, Colletti L, Greco Lucchina A, Del Fabbro M. Dental implant survival rate in irradiated and non-radiated patients: a systematic review and meta-analysis. J Biol Regul Homeost Agents: Mar-Apr;35(2 Suppl. 1):53-65; 2021.

63. Smith Nobrega A, Santiago JF Jr, de Faria Almeida DA, Dos Santos DM, Pellizzer EP, Goiato MC. Irradiated patients and survival rate of dental implants: A systematic review and meta-analysis. J Prosthet Dent: Dec;116(6):858-866; 2016.

64. Chambrone L, Mandia J Jr, Shibli JA, Romito GA, Abrahao M. Dental implants installed in irradiated jaws: a systematic review. J Dent Res: Dec;92(12 Suppl):119S-30S; 2013.

65. Schiegnitz E, Al-Nawas B, Kämmerer PW, Grötz KA. Oral rehabilitation with dental implants in irradiated patients: a meta-analysis on implant survival. Clin Oral Investig: Apr;18(3):687-98; 2014.

66. Anderson L, Meraw S, Al-Hezaimi K, Wang HL. The influence of radiation therapy on dental implantology. Implant Dent: Feb;22(1):31-8; 2013.

67. Di Carlo S, De Angelis F, Ciolfi A, Quarato A, Piccoli L, Pompa G, Brauner E. Timing for implant placement in patients treated with radiotherapy of head and neck. Clin Ter: Sep-Oct;170(5):e345-e351; 2019.

68. Patel J, Antov H, Nixon P. Implant-supported oral rehabilitation in oncology patients: a retrospective cohort study. Br J Oral Maxillofac Surg: Oct;58(8):1003-1007; 2020.
