# Supplementary material for: Survival of dental implants in irradiated head and neck cancer patients compared to non-irradiated patients: An umbrella review
Source: PLoS One. 2025 Sep 3;20(9):e0324388. doi: 10.1371/journal.pone.0324388 (PMC12407548; doi:10.1371/journal.pone.0324388)
Supplement: Attachment — Submitted filename: Response to Reviewers Amitha Basheer N.pdf [file pone.0324388.s005.pdf]

Reviewer #3: Amitha Basheer N

Thank you very much for your help and correction of the article. We hope we were able to correct.

Reviewer #3: The manuscript addresses an important topic and follows a rigorous methodology. However, the introduction and discussion need improved readability, with a clearer focus on clinical implications. The inclusion of grey literature requires justification, and tables should be refined for clarity and alignment. Balance the critique of low-quality studies with practical relevance and actionable recommendations for future research. Ensure consistent referencing and formatting throughout.

Click on the highlighted portions in the manuscript to see detailed comments in the notes. Comments: The manuscript can be accepted after few minor grammatical corrections.

The suggestion is implemented and structured in the other corrections requested by other reviewers as well. The necessary changes and corrections requested have been sent in the article, we hope we have been able to live up to the occasion.

Authors' concluding remarks: Endnote was used to remove all duplicate references at once. For item 2.7 Results Synthesis, an excerpt from the text was removed due to the use of the arithmetic mean of the raw data to find the percentage.
