# Supplementary material for: Survival of dental implants in irradiated head and neck cancer patients compared to non-irradiated patients: An umbrella review
Source: PLoS One. 2025 Sep 3;20(9):e0324388. doi: 10.1371/journal.pone.0324388 (PMC12407548; doi:10.1371/journal.pone.0324388)
Supplement: Attachment — Submitted filename: Comment.docx [file pone.0324388.s003.docx]

Comments: The manuscript can be accepted after few minor grammatical corrections.
